# Supplementary material for: Generation of left ventricle-like cardiomyocytes with improved structural, functional, and metabolic maturity from human pluripotent stem cells
Source: Cell Rep Methods. 2023 Apr 24;3(4):100456. doi: 10.1016/j.crmeth.2023.100456 (PMC10163040; doi:10.1016/j.crmeth.2023.100456)
Supplement: Document S2. Article plus supplemental information [file mmc11.pdf]

Cell Reports
Methods

Generation of left ventricle-like cardiomyocytes with improved structural, functional, and metabolic maturity from human pluripotent stem cells

Graphical abstract

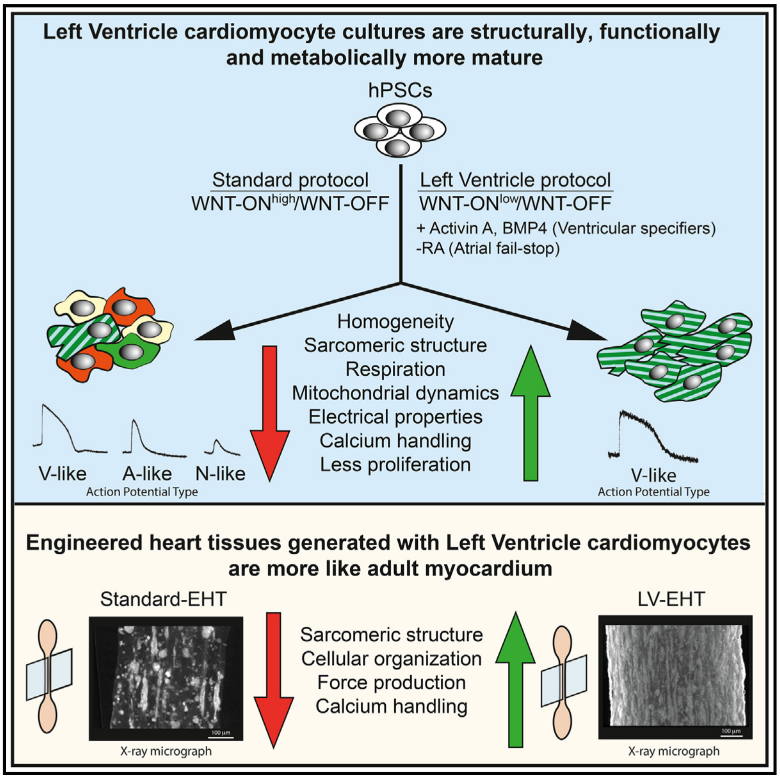

Highlights

- Left ventricle (LV) mesoderm precursors retain atrial and right ventricle potential
- Blocking retinoic acid signaling prevents atrial escapee emergence
- LV cardiomyocytes quickly attain functional, structural, and metabolic maturity
- Mechanical load-induced maturity is additive rather than transformative

Authors

Nicola Dark, Marie-Victoire Cosson, Lorenza I. Tsansizi, ..., Sian E. Harding, James C. Smith, Andreia S. Bernardo

Correspondence

andreia.bernardo@crick.ac.uk

In brief

Dark et al. report that near-homogeneous human left ventricle cardiomyocytes can be rapidly derived from human pluripotent stem cells. These cells exhibit enhanced maturity compared with other age-matched cardiomyocyte cultures. The homogeneity and maturity of these cells renders them a suitable model of left ventricle development and disease and should enable more faithful cardiotoxicity screens.

Dark et al., 2023, Cell Reports Methods *3*, 100456
April 24, 2023 Crown Copyright © 2023
https://doi.org/10.1016/j.crmeth.2023.100456

CellPress

Cell Reports Methods

CellPress
OPEN ACCESS

Article

Generation of left ventricle-like cardiomyocytes with improved structural, functional, and metabolic maturity from human pluripotent stem cells

Nicola Dark,[1] Marie-Victoire Cosson,[1,5] Lorenza I. Tsansizi,[1,2,5] Thomas J. Owen,[2] Elisa Ferraro,[1] Alice J. Francis,[2] Selina Tsai,[1] Camille Bouissou,[1] Anne Weston,[1] Lucy Collinson,[1] Najah Abi-Gerges,[3] Paul E. Miller,[3] Kenneth T. MacLeod,[2] Elisabeth Ehler,[4] Richard Mitter,[1] Sian E. Harding,[2] James C. Smith,[1] and Andreia S. Bernardo[1,2,6,*]

[1]The Francis Crick Institute, London, UK
[2]NHLI, Imperial College London, London, UK
[3]AnaBios, San Diego, CA, USA
[4]Kings College London, London, UK
[5]These authors contributed equally
[6]Lead contact
*Correspondence: andreia.bernardo@crick.ac.uk
https://doi.org/10.1016/j.crmeth.2023.100456

MOTIVATION Impaired LV-cardiomyocyte function caused by myocardial infarction (MI), ventricular arrhythmias, or drug-induced cardiotoxicity are among the most common and debilitating cardiovascular diseases, often leading to organ dysfunction and failure.[86,87] *In vitro* models of LV cardiomyocytes are, therefore, highly desirable for modeling LV cardiovascular diseases and for developing novel therapies to treat them. Here, we developed a method to differentiate rapidly and efficiently near-homogeneous populations of LV cardiomyocytes from human pluripotent stem cells (hPSCs). These cardiomyocytes are also more mature than those generated with the standard WNT-ON/WNT-OFF protocol, thus making them an attractive model.

SUMMARY

Decreased left ventricle (LV) function caused by genetic mutations or injury often leads to debilitating and fatal cardiovascular disease. LV cardiomyocytes are, therefore, a potentially valuable therapeutical target. Human pluripotent stem cell-derived cardiomyocytes (hPSC-CMs) are neither homogeneous nor functionally mature, which reduces their utility. Here, we exploit cardiac development knowledge to instruct differentiation of hPSCs specifically toward LV cardiomyocytes. Correct mesoderm patterning and retinoic acid pathway blocking are essential to generate near-homogenous LV-specific hPSC-CMs (hPSC-LV-CMs). These cells transit via first heart field progenitors and display typical ventricular action potentials. Importantly, hPSC-LV-CMs exhibit increased metabolism, reduced proliferation, and improved cytoarchitecture and functional maturity compared with age-matched cardiomyocytes generated using the standard WNT-ON/WNT-OFF protocol. Similarly, engineered heart tissues made from hPSC-LV-CMs are better organized, produce higher force, and beat more slowly but can be paced to physiological levels. Together, we show that functionally matured hPSC-LV-CMs can be obtained rapidly without exposure to current maturation regimes.

INTRODUCTION

Human pluripotent stem cells (HPSCs) hold the potential to produce an unlimited supply of human cardiomyocytes and offer the scalability needed for pharmaceutical applications.[1–3] However, the most widely used hPSC-derived cardiomyocyte (hPSC-CM) preparations typically contain a proportion of hPSC-CMs with ventricular, atrial, or nodal phenotypes, and generally lack maturity.[4–7] Even those preparations with an enriched ventricular phenotype have not been identified as right, left, or a mix of ventricle cardiomyocytes.[8] These cardiomyocyte populations typically resemble fetal-like cardiomyocytes and display low sarcomere organization, low sarcoplasmic reticulum and mitochondria content, underdeveloped calcium handling capacity, and poor mitochondrial oxidative capacity.[9,10] The use of mixed and immature populations poses challenges because cellular

Crown Copyright © 2023
This is an open access article under the CC BY license (http://creativecommons.org/licenses/by/4.0/).

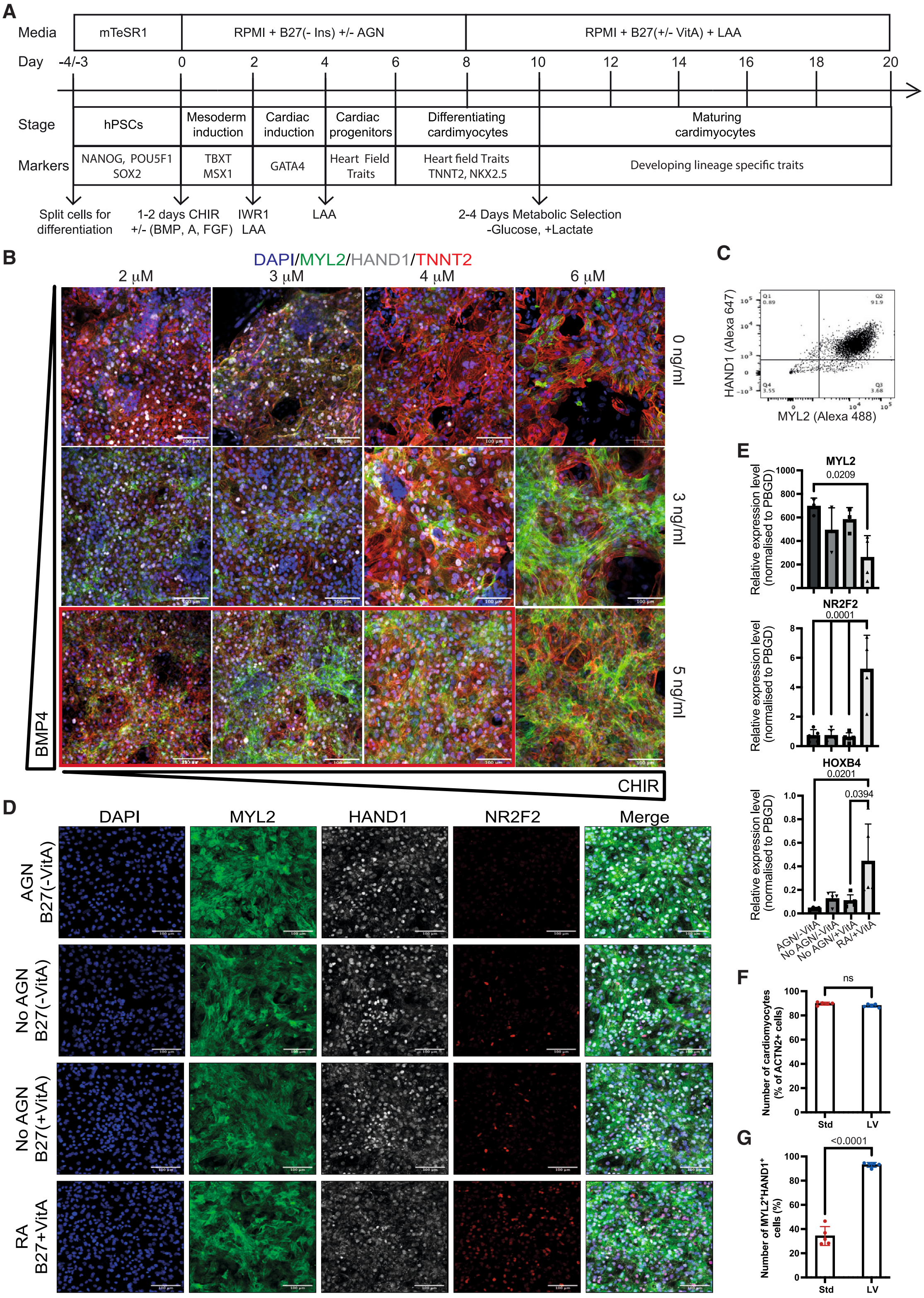

Figure 1. Generation of hPSC-LV-CMs using a two-step approach

(A) Depiction of the standard WNT-ON/WNT-OFF protocol; modifications to this protocol (±) and critical feeding steps are indicated. CHIR, CHIR99021; BMP, BMP4; A, activin A; FGF, FGF2; LAA, L-ascorbic acid; IWR1, WNT pathway inhibitor; AGN, AGN193109, a pan-retinoic acid (RA) inhibitor; VitA, vitamin A.

(legend continued on next page)

heterogeneity/immaturity can confound study outcomes or render cells suboptimal for downstream cell therapy approaches. The ability to produce mature left ventricle (LV)-specific hPSC-CMs (hPSC-LV-CMs) would circumvent many barriers, allowing a more reliable and extensive use of this technology to model and treat LV disorders.

The heart is a mesodermal derivative that develops from cells that migrate out of the primitive streak during gastrulation to give rise to the cardiac crescent.[11] Cardiac lineage specification occurs during the initial stages of gastrulation as shown by several lineage-tracing studies and validated *in vitro* using hPSCs.[8,12–15] A seminal hPSC study revealed the importance of manipulating the first stage of the differentiation process to produce specifically atrial or ventricular cardiomyocytes and further demonstrated the need to include retinoic acid (RA) in the medium to promote more efficient atrial differentiation.[8] This was in keeping with the known role of RA as a key regulator of cardiovascular cell fate; high RA has been linked to enlarged atria and smaller ventricles, while RA inhibition has been shown to promote the opposite effect.[16–19]

Atrial and ventricular cardiomyocytes are significantly different cell types, while right ventricle and LV cardiomyocytes are more similar. Despite these similarities, LV and right ventricle cardiomyocytes arise from different progenitors and display structural, electrophysiological, metabolic, and calcium handling differences.[11,20,21] Of note, the LV develops first and faster from progenitors coming from the mid streak, which coalesce in the first heart field, a cardiac region known to express TBX5, HCN4, and NKX2.5. On the other hand, the right ventricle develops later from progenitors coming from the late streak, which end up in the secondary heart field, a cardiac region known to express ISL1.[11,15,22] Single-cell RNA sequencing has further suggested that there are transcriptome dissimilarities between LV and right ventricle cardiomyocytes earlier in development.[21,23] By performing RNA sequencing on microdissected right ventricle and LV as well as atrial progenitors collected from the emerging mouse heart at embryonic day (E) 8–8.5, our lab has further shown that these chambers have unique gene signatures at the onset of heart development (unpublished data).

In this study, we revisited the idea that cardiomyocyte identity is dictated by the mesoderm differentiation regime, with the aim of optimizing a LV-specific differentiation protocol from hPSCs. We further tested the hypothesis that blocking the RA pathway would increase the percentage of ventricular cells. Overall, in just 20 days, we were able to generate near-homogeneous populations of LV-like cardiomyocytes. To our surprise, these were more mature than cardiomyocytes generated according to the standard protocol,[4,5] even without exposure to maturation factors. The speed at which our LV cardiomyocytes differentiate mimics the faster rate of maturity observed *in vivo* for LV cardiomyocytes.

RESULTS

Two-step approach to specify LV cardiomyocyte progenitors

Canonical WNT activation is the key first step of protocols to generate cardiomyocytes, by promoting the formation of mesoderm progenitors.[4,5] The modulation of activin/BMP signaling is critical in directing the fate of these progenitors toward atrial or ventricular descendants.[8] However, the interplay between WNT and the BMP pathway has not been evaluated, despite the fact that supplementation with BMP is known to activate the WNT pathway[24] and that these pathways interact during *in vivo* mesoderm development.[25–27] We hypothesized that if we kept activin A levels at a low and constant level (5 ng/mL) while varying the amount of BMP and WNT signaling, we would identify the signaling microenvironments able to mimic the mid-to-late streak where Foxa2 expression starts. As this is the region from which ventricular progenitors emerge,[14,15] we predicted that our strategy would allow us to fine-tune the emergence of LV and right ventricle cardiomyocyte progenitors (Figure 1A).

To modulate the WNT pathway, we used CHIR (CHIR99021), a GSK3β inhibitor that promotes β-catenin stabilization and thus increases canonical WNT signaling. Post-mesoderm induction, the cells were subject to WNT inhibition (IWR1) as per the original protocol.[4,5] We first confirmed that our hPSCs were pluripotent and karyotypically normal (Figure S1). Cells were grown for 20 days and immunostained for the pan-cardiac marker troponin T (TNNT2), the pan-ventricular marker myosin light chain 2 ventricle isoform (MLC2V or MYL2), and the LV marker heart and neural crest derivatives expressed 1 (HAND1) (Figure 1B).[28–30] Results showed that BMP4 addition promoted the upregulation of MYL2, especially at higher doses. However, CHIR levels restrained the expression of HAND1, with lower doses enabling its expression, while at higher doses, HAND1 expression was lower or absent. To confirm the purity of the cells obtained in the optimal LV conditions, we performed flow cytometry. This showed that around 90% of the cells generated were LV-like cardiomyocytes as per MYL2 and HAND1 co-expression (Figure 1C). This suggested that LV-like cardiomyocytes can be efficiently generated using low activin A, 5 ng/mL BMP4, and low CHIR (2–3 μM). Similar results were obtained for other lines, although the optimal concentrations of BMP4 and CHIR varied (Figures S2A and S2B).

(B) Immunostaining micrographs of day 20 hPSC-CMs generated from mesoderm cells exposed to activin A, FGF2, and varying amounts of BMP4 and CHIR, demonstrating the cardiac corridor for making LV-like cardiomyocytes (red box). Scale bar: 100 μm.
(C) Flow cytometry plot of day 20 hPSC-LV-CM cultures.
(D and E) Day 20 hPSC-CMs generated in the presence or absence of RA signaling (±AGN, ±vitamin A, ±RA).
(D) Immunostaining micrographs; scale bar: 100 μm.
(E) qRT-PCR analysis.
(F and G) Cardiomyocyte quantification in day 20 hPSC-CM cultures differentiated using the standard (Std) or LV protocols. Cells were co-stained with ACTN2, MYL2, and HAND1. DAPI was used to normalize the data.
See also Figure S2.

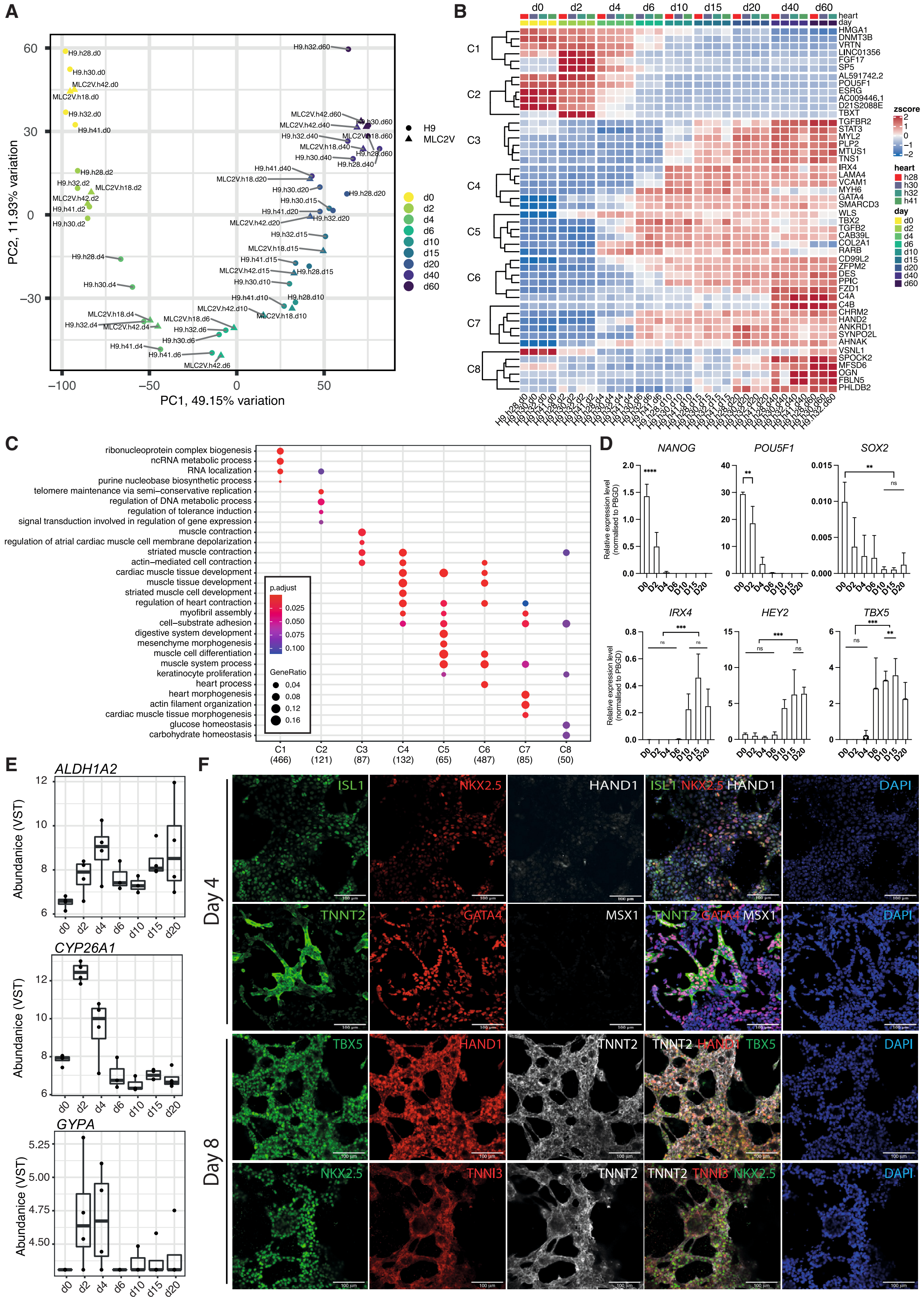

(legend on next page)

Given that the medium supplement used for the first stages of the differentiation (B27-insulin; Figure 1A) contains vitamin A, which can be converted into RA, a known atrial specifier, we next wanted to address the role of RA in cardiomyocyte fate specification. Since LV cardiomyocytes *in vivo* express low levels of RA target genes (Figure S2C), we hypothesized that RA inhibition using a pan-RA receptor inhibitor (AGN193109 [AGN]) would promote a stable differentiation and increased yield of LV cardiomyocytes. To identify atrial cells, we stained them for NR2F2 (also known as COUP-TFII), a marker essential for atrial development.[31] Interestingly, RA addition was unable to convert the entire population to an atrial fate and instead lead to a great proportion of the cells becoming right ventricle-like with 51% of the cells expressing MYL2 but not HAND1 (Figures 1D and S2D). On the other hand, addition of a pan-RA inhibitor or removing vitamin A from the second part of the protocol did not lead to significant changes in the proportion of LV cardiomyocytes (Figures 1D and S2D). However, we detected consistently small numbers of atrial descendants in cultures where RA was not inhibited even if no vitamin A was present in the medium during the second phase of the protocol (Figure 1D). Expression analysis further confirmed the +AGN/−vitamin A (VitA) condition had a significant increase in MYL2 expression and a decrease in both NR2F2 and HOXB4 (an atrial marker usually downstream of RA signaling) compared with RA-treated samples (Figure 1E). These data suggest that this mesoderm regime generates cells primed to become LV cardiomyocytes but which are not fully committed, as they can still form atrial and right ventricular cells if exposed to RA or when cultured in VitA.

To further understand the cells' RA response, we performed an ALDH assay. Neither condition (±AGN/±VitA) had elevated levels of ALDH activity within cardiac mesoderm populations, further demonstrating that the mesoderm induction gave rise to cells with little capacity to convert retinol into RA (Figure S2E). This result is in keeping with the poor atrial differentiation seen in RA-treated cultures (Figure 1D) and suggests that blocking the RA pathway with AGN prevents $ALDH^+$ escapees from becoming atrial cardiomyocytes.

To evaluate LV properties in our cardiomyocytes and in those generated using the most widely used ("standard") differentiation protocol,[4,5] we performed side-by-side immunostaining. Our protocol readily generated LV-like cardiomyocytes, while the standard protocol led to the generation of a more heterogeneous population (Figure S2F). Quantification confirmed that while there was no difference in the percentage of cardiomyocytes produced by both protocols, the percentage of LV-like cells was significantly different (Figures 1F and 1G).

Next, we performed RNA sequencing of our LV cardiomyocytes from 2 different cell lines and compared the expression of genes previously shown to be enriched in human fetal LV cardiomyocytes[23] across our samples and cardiomyocytes generated using other protocols[6,32–34] (Figures S2G and S2H). While these genes are expressed across all cardiomyocytes tested, our LV cardiomyocytes express higher levels of most of the LV-associated genes as demonstrated by the heatmap and confirmed via the boxplot showing the mean variance-stabilized abundancies for the LV genes. Cardiomyocytes within the Giacomelli dataset were the closest to our LV cardiomyocytes. However, they expressed, on average, lower or the same mean levels of the overall LV-associated genes. On the other hand, cardiomyocytes generated using the Kuppusamy or standard protocol (Branco and Cyganek.V datasets) expressed lower mean levels of these markers. Of note, the lowest mean expression of LV-specific genes was observed in RA-treated cells (atrial dataset, Cyganek.A), as would be expected for an atrial population. These results corroborate the transcriptional signature of our LV cardiomyocytes.

To evaluate how our LV cardiomyocytes compared with human *in vivo* cardiomyocytes, we grew cultures up to day 60 and compared them with human adult heart tissue samples or isolated cardiomyocyte samples (Figure S2I). Principal-component analysis confirmed that the *in vivo* and *in vitro* samples are distinct (principal component 1 [PC1], 53% variation), but it also demonstrated that the LV cardiomyocytes align better with the ventricular tissues (PC2/PC3), in keeping with the ventricular identity of the samples as defined by staining (Figures 1B–1D). As expected, we were not able to distinguish between adult LV and right ventricle samples,[23,35] and our samples aligned with both adult ventricular samples.

Together, these findings suggest homogeneous LV-like cardiomyocyte differentiation from hPSCs relies on signaling environment manipulation at the mesoderm stage and that blocking RA acts as a fail stop to prevent atrial differentiation in the cultures. We thus refer to these cells hereon as hPSC-LV-CMs.

hPSC-LV-CMs arise from first heart field progenitors

To ascertain if the hPSC-LV-CMs followed the expected lineage-specification trajectory, we next performed a time course RNA sequencing (RNA-seq) analysis covering samples from days 0 to 60 of differentiation. PC analysis (PCA) demonstrated that most of the sample variation (PC1, 49.15%/PC2, 11.93%) explains the lineage trajectory (Figure 2A). Poisson dissimilarity scores demonstrated that there is a high transcriptional similarity between all samples past day 10/15 of differentiation (Figure S3A). Heatmap of pairwise comparisons (Wald test) between consecutive time points further confirmed this finding (Figure S3B).

Figure 2. hPSC-LV-CMs arise from first heart field progenitors
(A) Principal-component analysis (PCA) of hPSC-LV-CM differentiation time course; days are indicated (d).
(B) Heatmap of the top 6 genes enriched in each cluster (C).
(C) GO-term analysis showing the top 4 terms in each cluster.
(D) qRT-PCR analysis for the indicated days (D). $^{**}p \leq 0.01$ $^{***}p \leq 0.001$, $^{****}p \leq 0.0001$.
(E) Expression levels as variance-stabilizing counts (VSTs).
(F) Immunostaining micrographs of day 4 and 8 cultures generated using the hPSC-LV-CM protocol. Scale bar: 100 μm.
See also Figure S3 and Table S1.

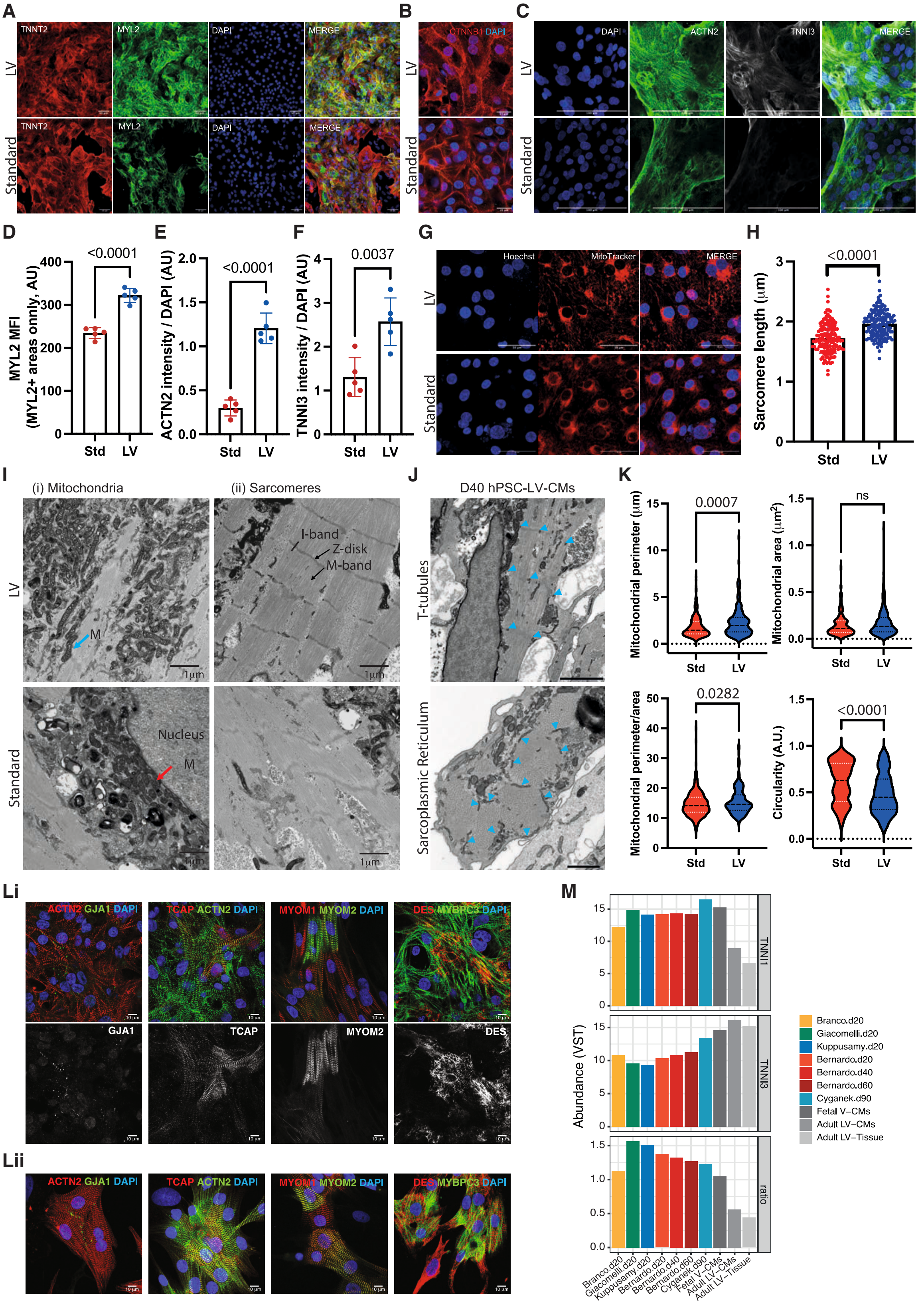

(legend on next page)

Next, a likelihood ratio test (LRT) was used to find genes that change in expression across replicate groups, i.e., time points. Divisive hierarchical clustering was then applied to the top 2,000 most significant genes to identify clusters of genes showing similar expression profiles across time, and each cluster was subject to Gene Ontology (GO) analysis (Figures 2B, 2C, and S3C; Table S1). The gene clusters showed a distinct transcriptional behavior over time that followed either a descending trajectory from day 2 (clusters 1 and 2) or an ascending trajectory from days 2–10 (clusters 3, 4, 5, 6, and 7) or from day 20 (cluster 8) (Figures S3C and 2B). For example, pluripotency genes (e.g., *SOX2*, *NANOG*, *POU5F1*) could be found in clusters 1 and 2, in keeping with the fact that differentiating cells are moving away from pluripotency. Cluster 2 was also enriched for blastocyst differentiation genes (e.g., *TBXT*, *CER1*), which are enriched during mesoderm differentiation at day 2 but are downregulated later. Genes involved in cardiac muscle development (e.g., *MEIS1*, *GATA5*, *GATA4*, *SMARCD3*, *HEY2*, *NKX2*.5, *TBX5*, and *IRX4*) were found in clusters 4, 5, and/or 6, which consisted of genes that plateaued by day 10. Of note was the presence of the ventricular-associated transcription factors *HEY2*[36] and *IRX4*,[37] the LV-specific progenitor marker *TBX5*,[38] and *MEIS1*, a critical regulator of cardiomyocyte cell cycle.[39] Moreover, genes involved in heart/cardiac muscle contraction were enriched in clusters 3–8 and included the pan-cardiac markers *TNNT2*, *TNNI3*, *TCAP*, and *MYH6* and the ventricular markers *MYH7* and *MYL2*, all of which are components of the sarcomeres.[40,41] Also associated with these clusters was *TIMP2*, a metalloproteinase inhibitor gene highly expressed in myocardium and whose mutant phenotype includes severe LV dysfunction.[42] We also noted that cluster 8, the only cluster consisting of genes still increasing past day 20, was enriched for glucose and carbohydrate homeostasis genes such as *FOXO1*, which is crucial for sustaining cardiomyocyte metabolism and cell survival,[43] and *WFS1*, which is involved in muscle mitochondrial and calcium metabolism.[44,45] We confirmed some of these gene trends using real time RT-PCR analysis (Figures 3D and S3D).

A previous study had suggested that ventricular cardiomyocytes arise from cells that express CYP26A1 and CD35a (GYPA) but lack ALDH1A2.[8] Our data confirmed the presence of *CYP26A1* at day 2 as well as the low expression of *ALDH1A2* throughout, in keeping with the ALDH flow cytometry results (Figures 2E and S2E). However, we were only able to detect very low levels of *GYPA* in some (but not all) replicates (Figure 2E), suggesting that GYPA is either very transient or it may not be a marker of LV progenitors.

We next used immunostaining to further validate the gene trends at the protein level (Figures 2F and S3E). At the mesoderm stage (day 2), most of the cells expressed TBXT, EOMES, and MSX1, and a high percentage also expressed GATA4. However, only a small percentage of the cells expressed FOXA2 (a trend seen also at day 1; data not shown), suggesting that not all the ventricular cells arise from $FOXA2^+$ progenitors, which would be in keeping with the reported low contribution of $Foxa2^+$ primitive streak cells to the LV (about 9.5% of LV area[15]). At day 4, most cells expressed GATA4 and ISL1 but had lost MSX1 expression as expected.[46] A proportion had also started to express HAND1 and NKX2.5, and, notably, a few cells were already expressing TNNT2. By day 8, cells had lost ISL1 expression, were expressing higher NKX2.5 and TNNI3, and were co-expressing HAND1, TBX5, and TNNT2. This is also the time frame when HCN4, another first heart field marker,[22] started to be expressed (Figure S3D). TBX5, HAND1, and HCN4 expression at this stage is consistent with first heart field-like specification, the region of the heart from where LV cardiomyocytes arise *in vivo*.[22,38,47]

To explore further if hPSC-LV-CMs transit via a first heart field-like stage, we compared our day 6/10 cardiomyocytes with isolated fractions of day 7/8 cardiomyocytes.[48] These fractions consisted of either $NKX2.5^+$ (G^+R^-), $TBX5^+$ (G^-R^+), $NKX5^+$/$TBX5^+$ (G^+R^+), or $NKX2.5^-$/$TBX5^-$ (G^-R^-) cells. PCA showed that our cells grouped closest to the samples expressing NKX2.5 alone or both TBX5 and NKX2.5, which were previously identified as secondary and primary heart field progenitors, respectively, and were only distinguished by TBX5 expression (Figure S3F). Since our cells co-express TBX5 and NKX2.5 (Figure 2F), we concluded that our day 6–10 cardiomyocytes are a first heart field progenitor population.

Together, these data indicate our protocol directs cells via a mesoderm population, which progress to become first heart field progenitors, thus following the expected developmental trajectory of LV cardiomyocytes.

hPSC-LV-CMs mature fast *in vitro*

Given that at day 8, our LV cardiomyocytes already expressed TNNI3 (Figure 2F), a marker of mature cardiomyocytes,[40] we next wanted to assess whether this protocol was promoting a faster differentiation/maturation *in vitro*. To this end, we

Figure 3. hPSC-LV-CMs mature fast *in vitro*

Cytoarchitecture characterization of day 20 hPSC-CMs differentiated using the Std or LV differentiation protocols.

(A–C) Immunostaining micrographs. Scale bars: 50 μm (A), 21 μm (B), and 100 μm (C).

(D–F) Fluorescence intensity quantification.

(G) Micrographs showing hPSC-CMs where mitochondria were stained with MitoTracker. Scale bar: 50 μm.

(H) Sarcomere length quantification.

(I) Transmission electron micrographs. (i) Examples of mitochondria (M) next to sarcomeres (blue arrow) or nucleus (red arrow) are indicated. (ii) Sarcomere structures are indicated: Z-disk, I-band, and M-line. Scale bar: 1 μm.

(J) Transmission electron micrographs of day (D) 40 hPSC-LV-CMs showing T-tubules and sarcoplasmic reticulum networks (blue arrow heads). Scale bars: 1 μm (top) or 500 nm (bottom).

(K) Analysis of mitochondria based on TEM images.

(L) Immunostaining micrographs. Top panel (i) is hPSC-LV-CMs, and bottom panel (ii) is rat neonatal CMs. Scale bar: 10 μm.

(M) Expression levels (VSTs) and *TNNI1*/*TNNI3* ratio within different RNA-seq datasets.

See also Figure S4.

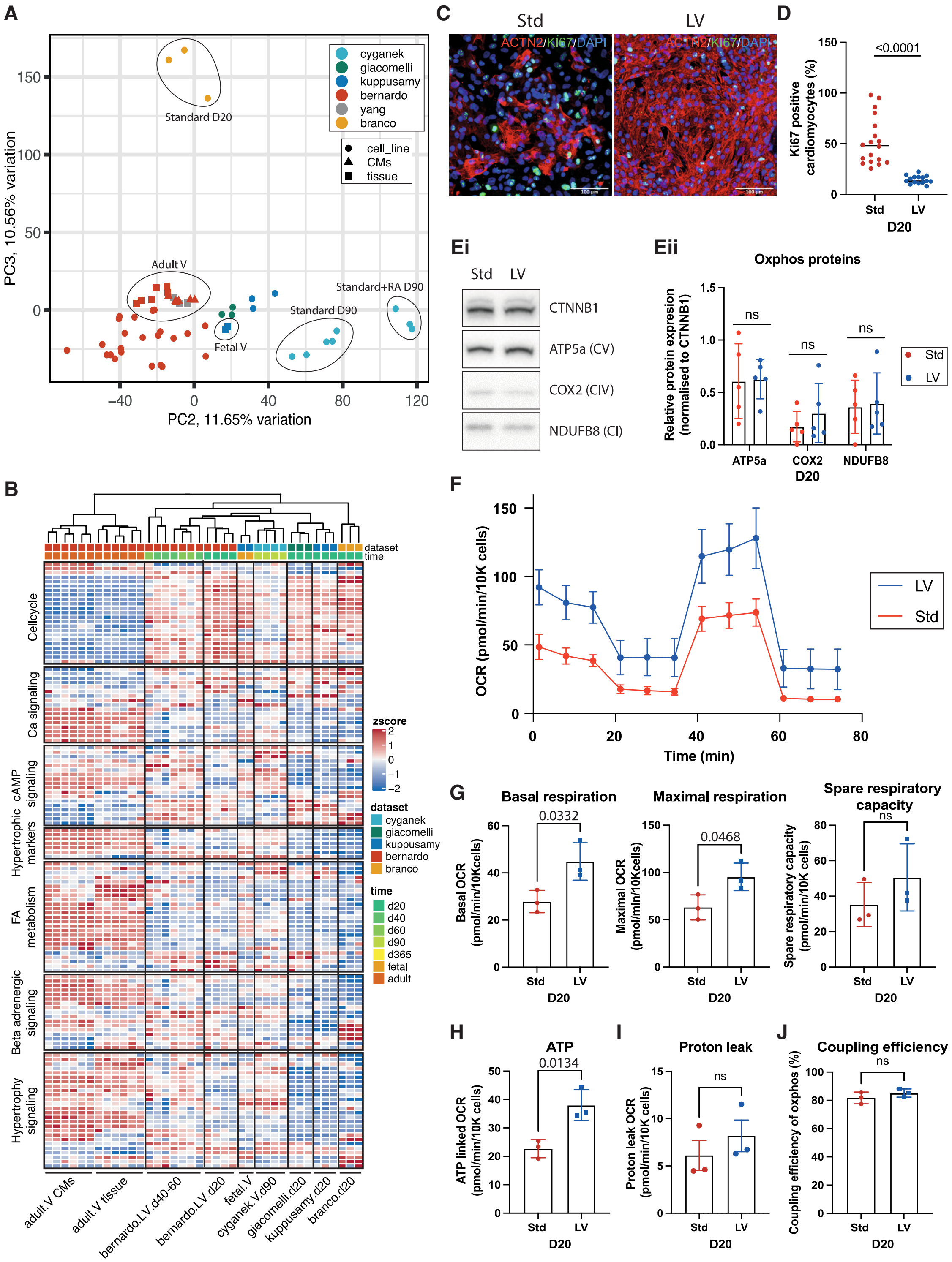

Figure 4. hPSC-LV-CMs are more mature than time-matched cardiomyocytes generated using alternative protocols

(A) PCA of human adult ventricular tissues, human adult isolated ventricular cardiomyocytes, human fetal ventricular tissues, and hPSC-CMs of various origins. *Ex vivo* samples were from the right (R), left (L), or both ventricles (V).

(B) Heatmap showing expression levels of a selection of genes involved in the pathways indicated.

(C) Immunostaining micrographs of day 20 hPSC-CMs (standard [Std] and LV). Scale bar: 100 μm.

(legend continued on next page)

compared hPSC-LV-CMs with cardiomyocytes generated following the standard differentiation protocol (hPSC-Std-CMs).[4,5] At day 20, hPSC-LV-CMs consisted of a near-homogeneous population of LV-like cardiomyocytes as opposed to the heterogeneous cultures obtained with the Std-cardiomyocyte protocol (Figures S2F and 3A). We also noted that hPSC-LV-CMs appeared to display well-defined sarcomeres (Video S1).

Thus, we next decided to investigate the cytoarchitecture of our cells. LV cardiomyocytes were more elongated, expressed higher levels of MYL2, ACTN2 (cardiac muscle α actinin), and TNNI3 (cardiac troponin I isoform 3), and had more mitochondria distributed along the myofibers than hPSC-Std-CMs (Figures 3B–3G). Moreover, hPSC-LV-CMs had longer sarcomeres (Figure 3H), in line with the length of more mature cardiomyocytes.[49,50] Electron microscopy further confirmed that hPSC-LV-CMs have better-organized sarcomeres with detectable Z-disks and I and A bands and some with detectable M bands. The presence of desmosomes or adherens junctions was also noted (Figures 3I and S4A). Better sarcomere definition was seen in day 40 cultures (Figure S4B), which was not surprising given that time in culture is known to promote cardiomyocyte maturation.[32,50] Likewise, we were able to detect evidence of transverse tubule-like structures (T-tubules) in day 40 hPSC-LV-CMs (Figure 3J, top). A tubular network of sarcoplasmic reticulum surrounding myofibrils was also noted in transverse sections, which is probably supporting the developing T-tubules (Figure 3J, bottom). Moreover, we confirmed that hPSC-LV-CMs have mitochondria often localizing between myofibers, which differed from the more perinuclear mitochondrial distribution seen in hPSC-Std-CMs (Figures 3G and 3I) and resembled the mitochondrial distribution of neonatal cardiomyocytes.[51] We further noted hPSC-LV-CMs have more elongated mitochondria than hPSC-Std-CMs as demonstrated by increased perimeter and perimeter/area and smaller circularity. The overall mitochondrial area was, however, unchanged, suggesting that the mitochondrial mass was the same in both cardiomyocyte cultures (Figures 3G and 3K). No obvious differences in mitochondrial development were seen between days 20 and 60 of differentiation (Figure S4C), in keeping with the similar mitochondrial DNA content observed (Figure S4D).

We next confirmed that some LV cardiomyocytes expressed the mature Z-disk marker TCAP (telethonin) and the mature M-band marker MYOM2 (myomesin 2, or M-protein), which are seen in a proportion of cells equivalent to that of rat neonatal cardiomyocytes (Figure 3L). We also confirmed the presence of gap junctions by staining cells for the mature gap junction marker GJA1 (Figure 3L). This was reassuring since these proteins are known not just for their mechanical function but also for their role in cardiomyocyte signaling.[52,53] Time in culture increased the sarcomere length, but it did not seem to increase the proportion of cells expressing these markers (Figure S4E; data not shown). Moreover, the cells started showing signs of stress from day 40, as reflected by the expression of the stress fiber marker ACTA2 (smooth muscle actin) (Figure S4F).

Expression analysis of the various RNA-seq datasets previously used for meta-analysis showed that the hPSC-LV-CMs expressed significantly higher levels of TNNI3 than other day 20 cultures grown in activin and BMP4 but lower than the standard day 90 dataset (Cyganek) or the fetal and adult cardiomyocytes. The levels of TNNI1 across cardiomyocyte cultures were, however, similar to those of fetal cardiomyocytes except for the Cyganek dataset, which expressed higher levels, or the Branco dataset, which expressed lower levels of this gene. It was therefore not surprising that the ratio of TNNI1 and TNNI3, as a measure of maturity, was lowest in adult cardiomyocytes but equivalent across the hPSC-LV-CM and the day 90 Cyganek datasets (Figure 3M), further validating the higher maturity of hPSC-LV-CMs compared with other day 20 cultures. Of note, the Branco dataset (generated from hPSC-Std-CMs), expressing the lowest level of TNNI1, displayed a low TNNI1/3 ratio, which highlights a limitation of this approach.

Overall, these findings suggest that hPSC-LV-CMs mature faster *in vitro* than hPSC-Std-CMs, in keeping with the faster rate of maturity seen *in vivo* for LV-cardiomyocytes.

hPSC-LV-CMs are more mature than time-matched cardiomyocytes generated using alternative protocols

To take a broader look at cellular maturity, we next compared the transcriptomic profile of the hPSC-LV-CMs, cardiomyocytes generated using other protocols,[6,32–34] and fetal and adult human cardiomyocytes (Figures 4A and S5A). PCA demonstrated that most of the sample variation (PC1, 38.02%) explains the difference between adult and hPSC-derived or fetal cardiomyocytes. Within PC1, fetal samples grouped closest with the hPSC-CMs kept in culture the longest (Cyganek dataset) and furthest from day 20 hPSC-CMs generated in the absence of exogenous activin and BMP4 supplementation (Branco dataset). This is in line with reports suggesting that time in culture promotes cardiomyocyte maturation[32] and further shows that activin and BMP supplementation during the mesoderm specification stage promotes higher cellular maturity.

Interestingly, PC2 and PC3 analysis placed the Giacomelli dataset closest to the fetal ventricle samples and our hPSC-LV-CMs closer to adult ventricle samples, while datasets generated in the absence of activin and BMP group the furthest from *in vivo* ventricular tissues/cardiomyocytes. This may reflect the cellular identity of the cells given that cardiomyocytes generated in the presence of activin and BMP express higher levels of ventricular markers (Figure S2H) and are therefore more likely to group closer to ventricular tissues. On the other hand, cardiomyocytes generated with the standard WNT-ON/WNT-OFF protocol group furthest away from ventricular samples likely because of their heterogeneous cell nature.[4]

(D) Graph showing the quantification of Ki67^{+} cardiomyocytes in day 20 hPSC-CMs.

(E) (i)Western blot analysis of oxphos proteins in day 20 hPSC-CMs. (ii)Quantification of oxphos protein expression in hPSC-CM cultures.

(F) Oxygen consumption rate (OCR) graph for day 20 hPSC-CMs (Std and LV).

(G–J) Graphs showing the respiration analysis (G), ATP production (H), proton leak (I), and coupling efficiency (J) of hPSC-CMs (Std and LV) based on OCR. See also Figure S5.

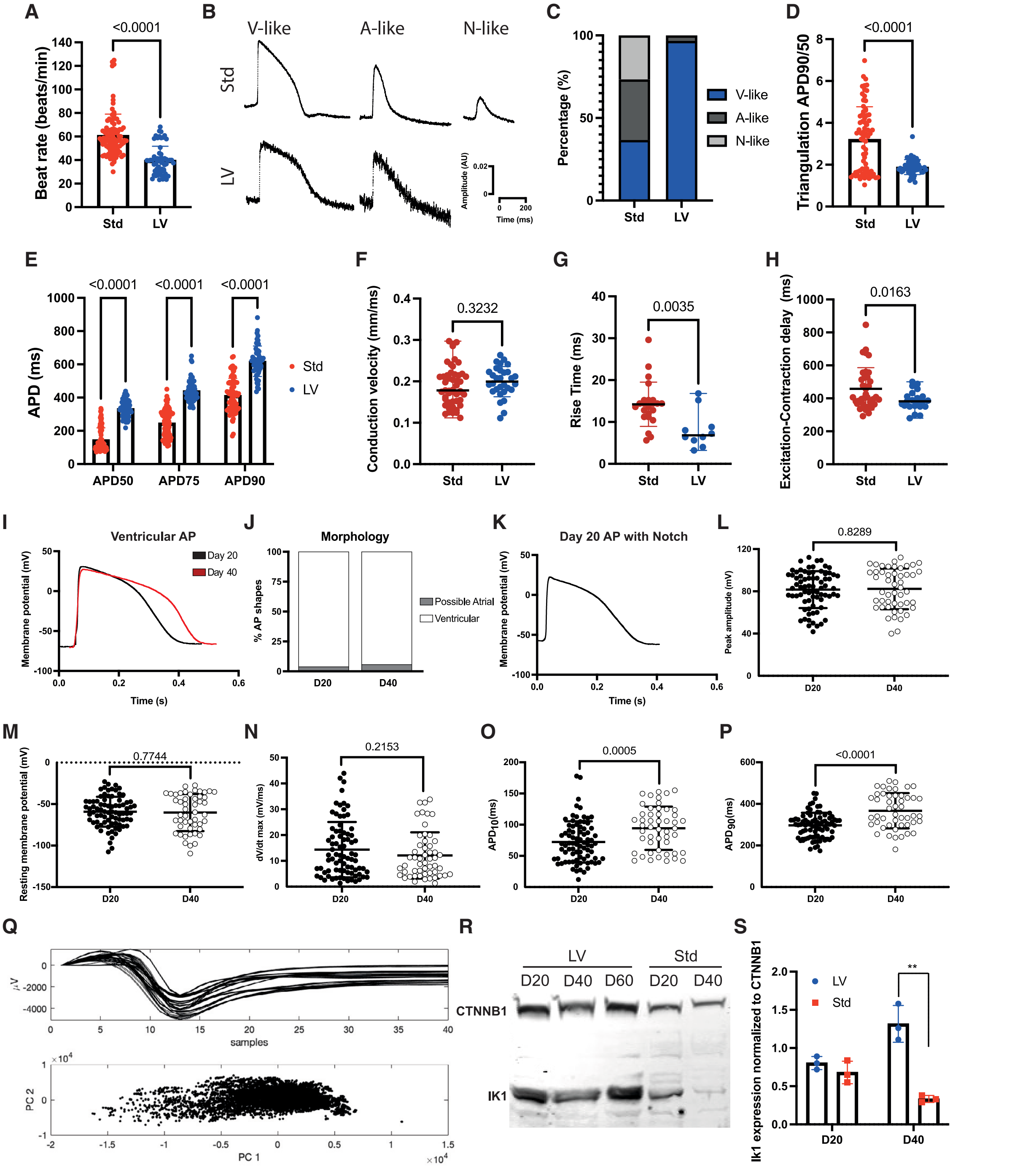

Figure 5. hPSC-LV-CMs display ventricular action potentials with hallmarks of electrophysiology maturity

Electrophysiology characterization of day 20 hPSC-CMs differentiated using the standard (Std) or LV differentiation protocols (A–H) using optical mapping (A–E) or microelectrode arrays (MEA) (F–H).

(A) Transient rate.

(B) Representative action potential (AP) shapes; V, ventricular; A, atrial; N, nodal.

(C) AP shape morphology assessment.

(D) Triangulation results.

(E) AP duration (APD) at different repolarization times (50%, 75%, or 90% to baseline).

(F) Conduction velocity.

(G) AP rise time.

(legend continued on next page)

Since a striking difference between the cytoarchitecture maturity of hPSC-LV-CMs and hPSC-Std-CMs was noted (Figure 3), we next focused our meta-analysis on various cardiac maturation markers as previously selected by Kuppusamy et al.[32] (Figure 4B). This analysis verified that all hPSC-CM samples analyzed group furthest from human adult cardiomyocytes and closer to the human fetal cardiomyocytes. Moreover, it showed that the older hPSC-CMs (Cyganek dataset) grouped the closest to fetal cardiomyocytes and that our day 20 hPSC-LV-CMs grouped closer to day 90 and fetal cardiomyocytes than any of the other day 20 cardiomyocyte populations analyzed, confirming the faster pace of maturity of hPSC-LV-CMs. Interestingly, there was a high similarity between the day 90 Cyganek samples and the day 40 hPSC-LV-CM samples, further reinforcing that our protocol leads to the generation of cells that mature faster *in vitro*.

One of the maturity classes of genes analyzed included a variety of cell cycle genes. In keeping with what is expected for more mature cardiomyocytes,[54] adult ventricular cardiomyocytes expressed low levels of these genes, while the younger hPSC-CMs expressed the highest levels (Figure 4B). Immunostaining for MKI67 (Ki-67), a marker of cells in active cell cycle, in day 20 cultures confirmed that cardiomyocytes within hPSC-LV-CM cultures express less Ki-67 than hPSC-Std-CMs (Figures 4C and 4D).

With regards to β-adrenergic signaling, cAMP signaling, and hypertrophy, there were two classes of genes: those expressed highest and those expressed lowest in adult samples. Fetal cardiomyocytes and older hPSC-CMs (including ours) expressed generally moderate levels of both (Figure 4B). We confirmed that time in culture increased (*CAMK2B*, *PDEC1*, *CAV3*, *KCNJ2*) or decreased (*PDE3B*, *NFATC2*, *HCN4*) the expression of some of these markers, bringing them to levels closer to those of adult cardiomyocytes (Figure S5B).

Lastly, we explored the expression of additional metabolism markers (Figure S5C). Except for the day 20 samples grown in the absence of activin and BMP4 (Branco dataset), we observed very few differences in the hPSC-CM data, which aligned well with the fetal samples. We noted specifically that in hPSC-LV-CMs, some fatty acid oxidation or synthesis genes (e.g., *FABP3*, *CPT1A*, *ACAT1*, *ACACB*) were increasing over time in culture, while some glycolysis genes (e.g., *PDK1*, *PGAM1*, *SLC2A3*) were decreasing (Figure S5C). It was, however, unsurprising that the hPSC-LV-CMs were not more metabolically active given that the medium in which they were cultured on was not enriched in fatty acids.

Importantly, we confirmed that while there was no overall difference in mitochondrial mass (Figure 4E), hPSC-LV-CMs had a better respiration capacity than hPSC-Std-CMs as demonstrated by their higher basal and maximal respiration rates and their higher ATP production (Figures 4F–4H and S5E). This may be explained by the differences in mitochondrial shape (Figure 4I).

To understand if the respiration capacity differences could be explained by the smaller amount of LV cardiomyocytes within Std cultures, we sorted MYL2-GFP$^+$ cells from both cultures for analysis. In keeping with the population results, ventricular cardiomyocytes generated using the LV protocol respired better (Figures S5D–S5H), suggesting that either ventricular cardiomyocytes matured faster or emerged earlier and had more time to mature in the LV protocol than in Std cultures.

Together, these data showed that day 20 hPSC-LV-CMs have maturity hallmarks close to those of aged hPSC-Std-CMs (day 90 Cyganek dataset), proliferate less, and have a better respiration capacity than age-matched hPSC-Std-CMs but, overall, still resemble fetal cardiomyocytes.

HPSC-LV-CMs display ventricular action potentials with hallmarks of electrophysiology maturity

To assess whether the hPSC-LV-CMs were also functionally more mature than hPSC-Std-CMs, we investigated their electrophysiological properties. By manually counting beats or using optical mapping, we noted that hPSC-LV-CMs had slower spontaneous beating than hPSC-Std-CMs (Figures 5A and S5A). Action potential (AP) analysis revealed striking differences in morphology and duration (AP duration [APD]) at various repolarization times, e.g., hPSC-LV-CMs had less triangulation and slower times to 90% repolarization (APD90) (Figures 5B–5E). In keeping with the ventricular nature of the hPSC-LV-CMs, 97% of these displayed ventricular AP morphologies, with fast upstrokes and plateau periods (Figures 5B and 5C). Conversely, in hPSC-Std-CM cultures, a mix of ventricular, atrial, and nodal APs were detected as expected (Figures 5B and 5C).[4] Moreover, hPSC-LV-CMs exhibited a faster rise time than hPSC-Std-CMs but similar conduction velocity, suggesting that they have some increased mature electrophysiological properties (Figures 5F and 5G). They also displayed a shorter excitation-contraction

(H) Excitation-contraction delay.
(I–P) Current-clamping analysis of day (D) 20 or 40 hPSC-LV-CMs.
(I) Representative AP traces.
(J) AP shape morphology assessment.
(K) Example of an AP with a notch.
(L) Peak AP amplitude.
(M) Resting membrane potential.
(N) dV/dt max.
(O) ADP at 10% repolarization.
(P) ADP at 90% repolarization.
(Q) Spike sorting analysis of field potentials from day 40 hPSC-LV-CMs. Top panel shows the field potentials as determined using MEA. Bottom panel shows PC1 and PC2 from the spike sorting analysis.
(R) Western blot analysis showing I_{K1} expression in hPSC-CMs.
(S) Quantification of I_{K1} expression.
See also Figure S6.

delay, demonstrating that hPSC-LV-CMs exhibit faster excitation-contraction (E-C) coupling (Figure 5H).

To further study the hPSC-LV-CMs, we performed microelectrode recordings, which confirmed that even at day 20, the APs of these cells have many of the hallmarks of ventricular cardiomyocytes (Figure 5I). In line with the optical mapping, only a few possible atrial-like APs (<5%) were detected in the cultures, and even these could correspond to more immature ventricular APs (Figures 5J and S6B). Interestingly, we identified multiple cells that displayed a notch, albeit small, confirming that some of these cells display evidence of transient outward current development even at day 20 (Figure 5K). We also noted that the AP amplitude of the cells corresponded to that of adult cardiomyocytes (~80–120 mV), with most cells displaying an amplitude above 80 mV upon stimulation (Figure 5L). The resting membrane potential of the cells was, on average, low (−60 to 70 mV), with some cells displaying a membrane potential comparable to that of adult ventricular cardiomyocytes, i.e., ~−75 to 90 mV (Figure 5M). However, the maximum depolarization velocity of the cells (dV/dt) was more like that of fetal cardiomyocytes (Figure 5N), which usually have a dV/dt_{max} <20 mV/ms.[55,56] Of note, in terms of electrophysiology maturity, the day 40 cardiomyocytes only outperformed the day 20 cardiomyocytes by the APD and AP area, which were longer/bigger in the older cells (Figures 5L–5P and S6B).

Given this evidence, we next used spike sorting of field potentials to evaluate the electrophysiology homogeneity of hPSC-LV-CMs and noted that PCA only distinguish one population of cells, confirming that these cultures were homogeneous (Figure 5Q). Next, we wanted to evaluate if higher KCNJ2 expression contributed to the higher maturity seen in hPSCs-LV-CMs since this gene is responsible for the inward rectifier current (I_{K1}) required for the stabilization of ventricular cells in a low resting membrane potential. As expected, hPSC-LV-CMs expressed higher levels of KCNJ2 than hPSC-Std-CMs but only at day 40 (Figures 5R and 5S).

Collectively, at an electrophysiological level, hPSC-LV-CMs display many phenotypic features ascribed as more developed than hPSC-Std-CMs, some of which were enhanced with time in culture. However, the electrophysiology of hPSC-LV-CMs was still immature compared to adult ventricular cardiomyocytes (reviewed in Kane and Terracciano[57]).

HPSC-LV-CMs have more adult-like calcium transients

To characterize the hPSC-LV-CMs functionally, we carried out calcium transient analysis using Fluo-4-AM and optical mapping. hPSC-LV-CMs had a slower transient rate (Figure S6D) in keeping with previous assessments (Figures 5A and S6A). Higher calcium transient (CaT) amplitude was seen in hPSC-LV-CMs, but these displayed overall slower calcium dynamics at day 20 compared with hPSC-Std-CMs (Figures 6A–6D). These results suggested the presence of functional internal calcium stores within both hPSC-CMs but with more immature calcium release mechanisms in hPSC-LV-CMs. This was, however, contradictory to the higher CaT amplitude (Figure 6B) and the increased expression of genes encoding several calcium handling and auxiliary proteins (Figure S6E) within hPSC-LV-CMs and could reflect the different beating rates of the cells.

We noted specifically that hPSC-LV-CMs upregulated cardiac ryanodine receptors (RYR2), a class of calcium channels located at the sarcoplasmic reticulum (SR) responsible for calcium release into the cytosol (Figures 6E and S6E). To confirm that these receptors were functional and to evaluate the dependency of cell contraction on SR Ca^{2+} release, a mechanism known as calcium-induced calcium release (CICR), we blocked them using ryanodine. Ryanodine treatment slowed the CaT of both hPSC-CM cultures, demonstrating that both use the SR to regulate calcium flux through the cell (Figures 6Fi and 6Fii). However, the cellular response to ryanodine was very different in these cultures: 1–5 μM ryanodine induced the opening of RYRs in hPSC-Std-CMs and only inhibited them at 10–20 μM concentrations, which contrasted with the strong inhibitory action of ryanodine at all concentrations tested in hPSC-LV-CMs (Figure 6Fiii). It has been demonstrated previously that in adult cardiomyocytes, ryanodine locks RYRs in an open subconductance state only at nanomole concentrations.[58] Thus, hPSC-LV-CMs are more sensitive to ryanodine, suggesting they have more SR-dependent CaTs.

Next, we evaluated if cells were equipped to remove calcium from the cytosol by measuring the expression of phospholamban (PLN) and SR/endoplasmic reticulum Ca^{2+}-ATPase (SERCA2, ATP2A2). The ATPase is responsible for the transport of calcium from the cytosol into the SR, and it is regulated by PLN. While the genes encoding these proteins were elevated in hPSC-LV-CMs (Figure S6E), only PLN was expressed at higher levels in hPSC-LV-CMs (Figures 6G and 6H). Yet, when we blocked SERCA2 with thapsigargin, hPSC-LV-CMs were more sensitive to the drug (Figure 6I), indicating a greater dependency of contraction on the SR. Both cultures had, however, slower CaT when exposed to thapsigargin, with a pronounced extended time to baseline suggesting that calcium reuptake by SERCA2 is required for calcium homeostasis within both CM cultures (Figures 6Ii and 6Iii).

We noted that time in culture had a minimal effect on most calcium genes studied, the exception being *RYR2*, ASPH, and *TRDN*, which were upregulated at the transcript level with time (Figures 6G, 6H, and S6F). Thus, we next determined if time improved the cells' calcium handling ability. Day 40 hPSC-LV-CMs had increased Ca^{2+} amplitude but minimal changes in CaTs when measured at spontaneous beat rate (Figures S6G–S6J). Compared with hPSC-Std-CMs, the amplitude was also the only obvious difference between the CaTs of these cells (higher in hPSC-LV-CMs), with hPSC-Std-CMs remaining marginally faster at a spontaneous beat rate (Figures S6K–S6N).

Collectively, day 20 hPSC-LV-CMs appear to have more functional SR Ca^{2+} stores and more effective CICR, albeit exhibiting slower CaTs, providing evidence that the more mature cytoarchitecture and electrophysiology enabled more mature function. Time in culture had a more marked effect on hPSC-Std-CMs, in line with the fact hPSC-LV-CMs mature earlier.

HPSC-LV-CMs generate more functional engineered heart tissues

To determine if the more mature phenotype of hPSC-LV-CMs would be reflected in the maturity of engineered heart tissues (EHTs), we made EHTs from both hPSC-LV-CMs (LV-EHTs)

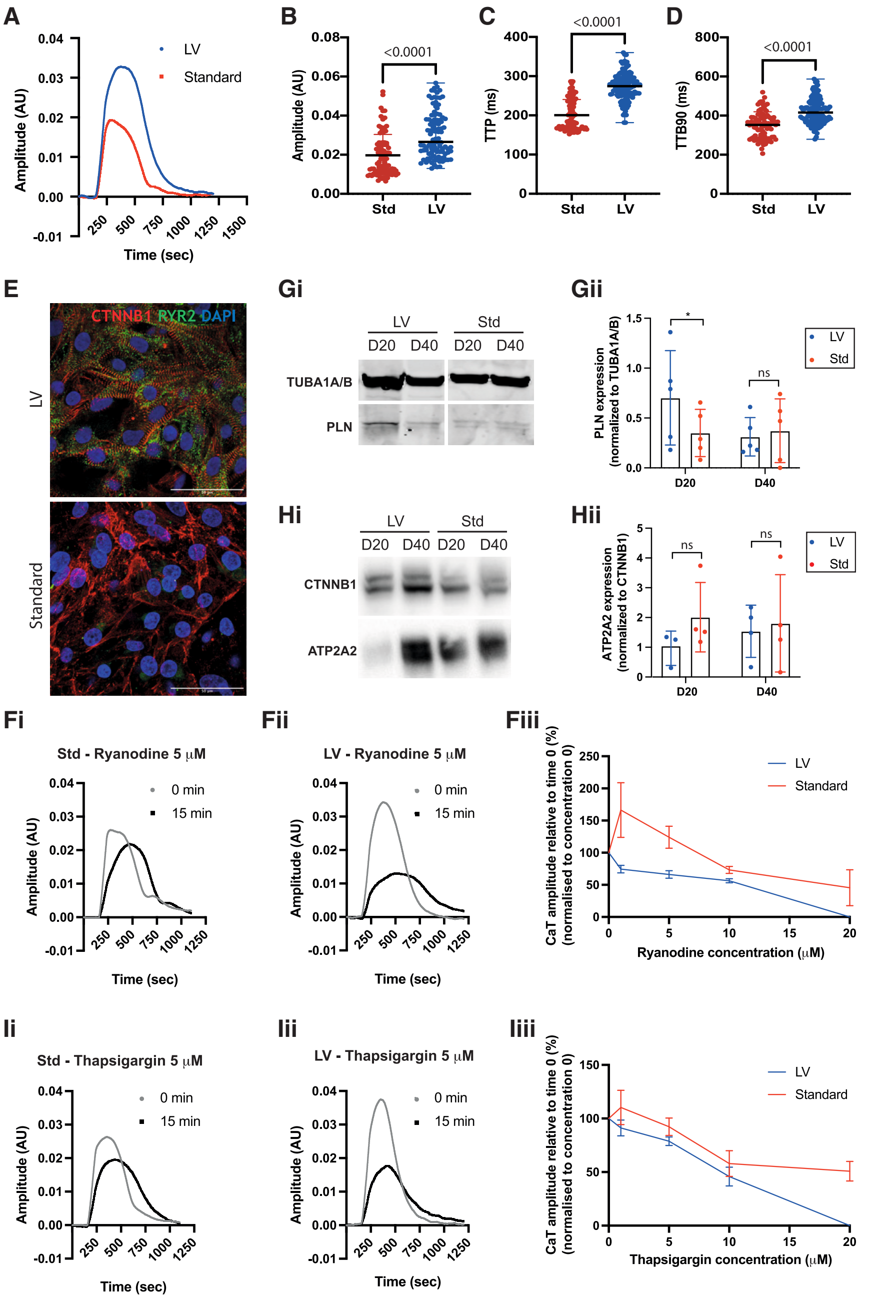

Figure 6. hPSC-LV-CMs have more adult-like calcium transients

Calcium function characterization of day 20 hPSC-CMs differentiated using the standard (Std) or LV differentiation protocols. Data were analyzed using optical mapping.

(A) Representative calcium transients (CaTs).

(B) CaT amplitude.

(C) CaT time to peak (TTP).

(D) CaT time to baseline 90 (TTB90).

(E) Immunostaining micrographs of hPSC-CMs. Scale bar: 50 μm.

(F) Study of RYR inhibition using ryanodine. (i and ii) Representative CaTs after exposure to 5 μM ryanodine. (iii) CaT amplitude after 15 min exposure to different ryanodine concentrations.

(G) (i) Western blot analysis showing PLN expression. (ii) Quantification of PLN expression.

(H) (i) Western blot analysis showing ATPA2 expression. (ii) Quantification of ATPA2 expression.

(I) Study of ATPA2 inhibition using thapsigargin. (i and ii) Representative CaTs after exposure to 5 μM thapsigargin. (iii) CaT amplitude after 15 min exposure to different thapsigargin concentrations.

See also Figure S6.

and hPSC-Std-CMs (Std-EHTs). Std-EHTs and LV-EHTs had a similar length and were both populated with cardiomyocytes throughout, but X-ray microscopy revealed significant morphological differences. Std-EHTs had a more irregular width and were thinner around day 15 (Figures 7A and 7B; Videos S2, S3, S4, S5, S6, and S7). We also noted that while in LV-EHTs, the cardiomyocytes organized themselves in interconnected bundles, and long stretches of cells could be seen aligned longitudinally throughout along the length of the EHT, in Std-EHTs, most cardiomyocytes did not form these bundles, and instead clumps of different sizes could be seen along the EHTs (Figure 7B; Videos S4, S5, S6, and S7).

Immunostaining confirmed this phenotype (Figures 7C and 7D), and transmission electron microscopy (TEM) showed that at a structural level, LV-EHTs exhibit T-tubules and tubular structures (likely SR) surrounding myofibrils (Figure 7E), further highlighting that LV-EHTs are developing appropriate cardiomyocyte architecture. It is unclear at this stage, however, why Std-EHTs did not have the ability to consistently form cardiomyocyte bundles and generated EHTs with a more uneven cellular distribution. Given that there is a reduction in the cell number per field of view in Std-EHTs (Figure S7D), despite similar numbers of cells used in their manufacture, it is possible that increased cell death may have contributed to the lower performance of Std-EHTs. This cannot, however, explain why LV-EHTs express increased levels of *HAND1*, *TNNI1*, *TNNI3*, and *KCNJ2* (Figure S7E) or why LV-EHTs have longer sarcomeres (Figure 7F).

The superior cytoarchitecture of LV-EHTs went hand in hand with slower beating rate and better function (Figures 7G–7M; Videos S2, S3, S4, S5, S6, and S7). Even those LV-EHTs that were no longer beating consistently could be paced (Figure S7A), suggesting that LV-EHTs are losing the pacemaker potentials typical of immature cardiomyocytes. Of note, some Std-EHTs struggled to follow the pace when stimulated, while, under the same standard pacing conditions, LV-EHTs followed steadily the pacing they were subjected to (Figure S7C). Functionally, LV-EHTs exhibited faster CaTs (Figures 7H, 7I, and S7B), faster

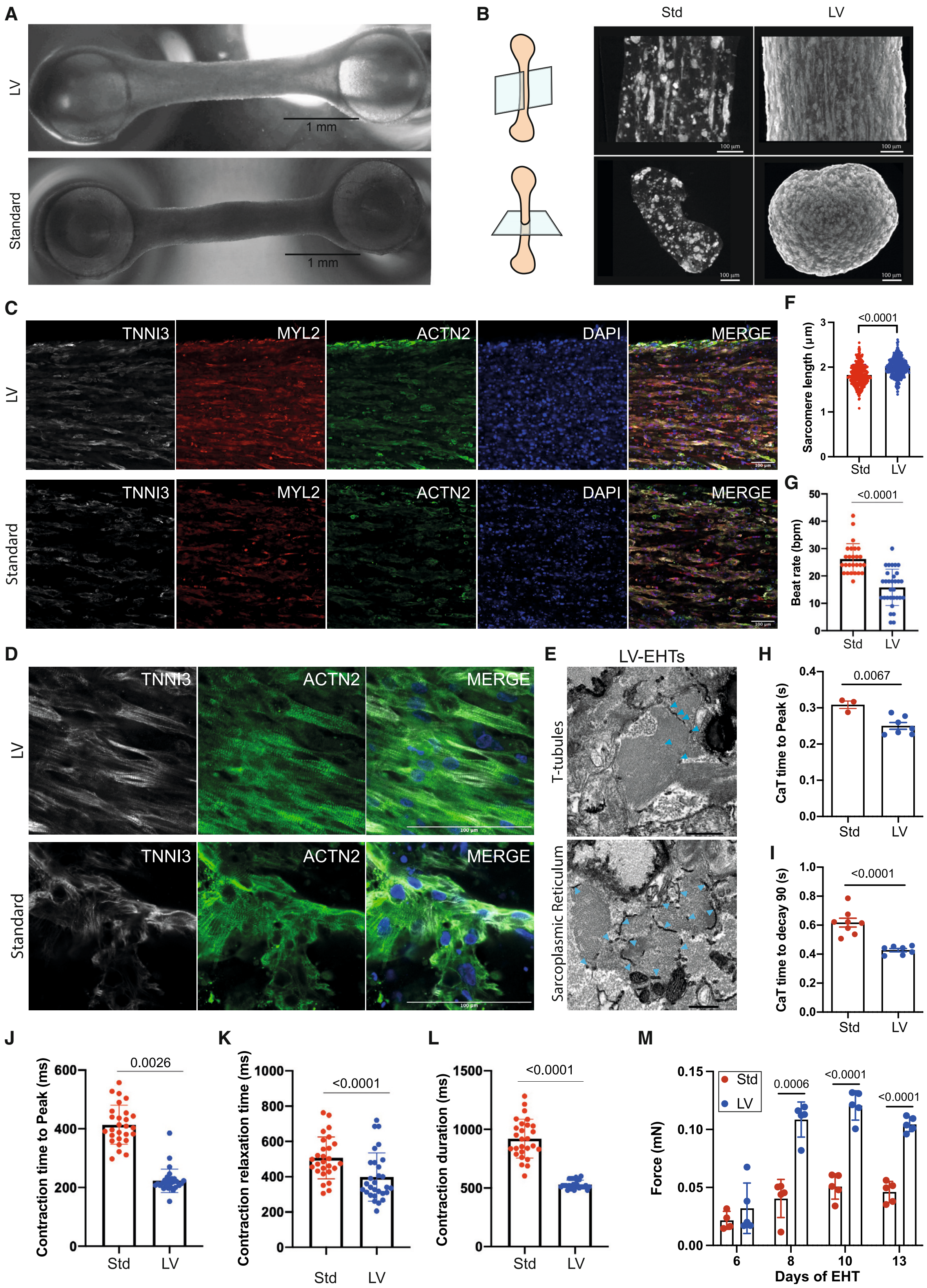

(legend on next page)

contraction dynamics (Figures 7J–7L), and enhanced contraction forces (Figure 7M). A decreased beating rate would be expected to slow contraction and relaxation in cardiac preparations. It is thus noteworthy that the LV-EHTs retained their rapid contraction and relaxation despite their markedly slowed beating rate.

Taken together, these data demonstrate the generation of EHTs, per se, is not able to rescue the lag in maturation present in the hPSC-Std-CMs, providing evidence that the enhanced maturity of hPSC-LV-CMs correlates with the higher quality/functionality of the LV-EHTs.

DISCUSSION

Here, we described a two-step approach for the rapid generation of near-homogenous LV-cardiomyocyte cultures with increased holistic mature properties. Our findings support the theory that LV cardiomyocytes arise from a unique mesoderm population, further extending the findings of Lee et al.[8] to demonstrate that appropriate mesoderm induction is critical for obtaining LV-specific cardiomyocytes. Modulating the WNT (via the GSK3b inhibition Chir) and BMP pathways was critical for the identification of an LV-progenitor corridor. The interplay between BMP and WNT pathways has been previously demonstrated during mesoderm specification.[24,59] Our results suggest further that *in vitro* LV cardiomyocyte progenitor specification relies on lower WNT and the presence of BMP.

Progenitor fine-tuning was not enough to completely abolish the presence of atrial NR2F2$^+$ cells from the cultures, but we circumvented this problem by the addition of a pan-RA inhibitor (AGN). RA signaling is necessary for the efficient generation of atrial cardiomyocytes from hPSCs.[8,60–62] However, the role RA inhibition may play to induce ventricular differentiation is unclear.[61,62] Our study is consistent with these reports and aligns with a model where LV progenitors are still able to generate right ventricular and atrial cardiomyocytes and where the absence of RA signaling prevents residual atrial differentiation. It further demonstrates that right ventricular differentiation requires RA exposure, in keeping with Yang et al.[63] *In vivo*, this RA signaling balance is coordinated by a combination of high CYP26A1 and low ALDH1A2 expression levels.[64] We demonstrate that while LV progenitors express CYP26A1 and have low ALDH activity, slightly elevated levels of ALDH1A2 expression at day 4 probably contribute to residual atrial differentiation. Thus, RA inhibition is linked to the inhibition of atrial differentiation in ventricle-primed cultures. While the RA inhibition role appears to be relatively minor, it enables the generation of cleaner ventricular cultures, which is of relevance for translating the potential of hPSCs-CMs for pharma- and therapeutical applications.

Monitoring cells over time revealed that LV progenitors transit via a ISL1/GATA4/NKX2.5 expressing stage prior to upregulating the first heart field and ventricular markers TBX5, HAND1, *HCN4*, *IRX4*, and *HEY2*.[22,29,38,47] ISL1-expressing cells contribute predominantly to secondary heart field descendants, but they also contribute to a substantial number of LV descendants including the trabecula and cells of the LV wall.[65] Our results are in line with the ISL1 lineage tracing and further suggest that ISL1 progenitors contribute to the first heart field and not directly to the LV. It is, however, unclear why apparently all ISL1$^+$ progenitors adopt an LV fate in our cultures. One plausible explanation is that ISL1 could be a broader cardiomyocyte progenitor in humans expressed in all cells before heart field progenitor specification. This would be in keeping with the fact human gastrula embryos express *ISL1* in both caudal and rostral mesoderm while *TBX5* is only expressed in rostral mesoderm.[66]

An unanticipated finding of our study was that hPSC-LV-CMs are more mature than cardiomyocytes generated using the standard cardiomyocyte protocol[4,5] in all indices of maturity measured (Table S2). It was particularly interesting to see that hPSC-LV-CMs were more sensitive to calcium blockers than hPSC-Std-CMs, implying that hPSC-LV-CMs could have more faithful pharmacological responses. Our results further indicate that day 20 hPSC-LV-CMs are closer to day 90 hPSC-Std-CMs than to other age-matched cardiomyocytes[32–34] in terms of maturity, suggesting that higher/faster maturity is an intrinsic result of cardiomyocyte progenitor patterning.

Studies aimed at maturing hPSC-CMs in monolayer cultures have used fatty acid-enriched medium and claim that this improves cardiomyocyte cytoarchitecture, respiration, force production, calcium kinetics, and AP upstroke velocity.[67,68] Despite the lack of fatty acids in the media, hPSC-LV-CMs exhibited similar improved features compared with hPSC-Std-CMs (Table S2), albeit it is difficult to directly compare results across studies. Time in culture has also previously been shown to improve hPSC-CM maturity.[32,69] Functionally, we noted that extended time in culture helped hPSC-LV-CMs develop: they had a more mature AP shape and duration, a higher CaT amplitude, and a longer sarcomere length. We also found evidence of developing T-tubules and an associated SR network in day 40 hPSC-LV-CMs. However, time in culture came at the expense

Figure 7. hPSC-LV-CMs generate more functional engineered heart tissues (EHTs)

Characterization of EHTs generated from day 40 hPSC-CMs differentiated using the standard (Std) or LV protocols.

(A) Light microscopy micrographs showing the overall structure of EHTs. Scale bar: 1 mm.

(B) Micrographs showing the ultrastructure of EHTs as obtained by X-ray microscopy. Top panels show longitudinal images. Bottom panels show transverse images. Scale bar: 100 μm.

(C and D) Immunostaining micrographs of EHTs. Images represent a single focal plane. Zoomed view (D). Scale bar: 100 μm.

(E) Transmission electron micrographs of LV-EHTs showing T-tubules (top) and sarcoplasmic reticulum networks (bottom) (blue arrow heads). Scale bar: 500 nm.

(F and G) Graphs showing the sarcomere length (F) and beat rate (G).

(H and I) Calcium transient (CaT) analysis of day 14/15 EHTs; CaT time to peak (H) and CaT time to decay 90 (I).

(J–L) Graphs showing contraction time to peak (J), contraction relaxation time (K), and contraction duration (L) of day 14/15 EHTs.

(M) Force generated at the indicated days post-EHT generation.

See also Figure S7.

of culture viability, with cells showing signs of stress and becoming increasingly difficult to lift even at day 40 (Figure S4F; data not shown). Primary adult cardiomyocyte cultures are known to detach and rapidly lose physiological function,[70–72] with surviving cells typically assuming a fetal phenotype.[70] Thus, it was reassuring to see higher maturity within EHTs, which provide a more natural 3D environment than monolayers in long-term 2D cultures.

Multiple 3D approaches have been used to promote cardiomyocyte maturity, with some relying on embryoid body-like multilineage cultures[33] and others on more sophisticated 3D cultures such as EHTs,[73,74] biowires,[75] or sustained pacing.[76] In this study, we chose to use EHTs to understand if a 3D environment could even out the maturity of hPSC-LV-CMs and hPSC-Std-CMs. Auxotonic contraction against a mechanical load, as promoted by EHTs, is known to improve the morphological, functional, and metabolic maturity of hPSC-CMs.[73,74,77–79] However, our data show that the mechanical load exerted by the EHTs was additive to the maturity of the cells of origin rather than transformative.

Interestingly, we also observed a slower beat rate within hPSC-LV-CMs and LV-EHTs. hPSC-CM spontaneous beating is due to the presence of the pacemaking current I_f and reduced densities of the hyperpolarizing current I_{K1}, which are lacking or are more prevalent in adult cardiomyocytes, respectively (reviewed in Goversen et al.[80]). These phenotypes are responsible for the proarrhythmic traits associated with these cells.[81] Elevated levels of I_{K1} in our cultures are likely an important factor in enabling loss of spontaneous beating in the hPSC-LV-CMs. This reduction of automaticity is an important feature of our cells as it reflects the higher electrophysiology maturity of the cells and will probably enable more faithful drug responses, bypassing some of the issues associated with the use of hPSC-CMs for *in vitro* drug screens. Likewise, the homogeneity and higher maturity of these LV cultures may facilitate their use in cell replacement therapy approaches, as they could potentially limit the transient post-transplant ventricular tachycardia seen in large-animal model transplantation studies using other hPSC-CMs.[82–85]

In conclusion, these results demonstrate that hPSC-LV-CMs are a suitable model to study LV development and disease and will likely enable more faithful LV-specific drug cardiotoxicity screens. Moreover, this work opens the possibility of like-for-like cell replacement therapy becoming an accessible treatment for patients with heart failure.

Limitations of the study

A question that will need to be addressed in the future is if hPSC-Std-CM cultures are less mature because they contain fewer LV cardiomyocytes. The different respiration capacity of isolated-ventricular cardiomyocytes from LV and Std populations (higher in LV) suggest that hPSC-LV-CMs are more mature, but we cannot rule out if the differences observed reflect right ventricle/LV maturity biases. Moreover, whether the same is true for other maturity hallmarks remains to be assessed.

HPSC-LV-CMs will also need to be tested further to (1) ascertain if they are more sensitive and/or produce more accurate readouts for drugs known to impact ventricular function and (2) determine if their physiology is approximate to that of bone fide adult ventricular cardiomyocytes.

In addition, to obtain maximal maturation, and thus maximal translation potential, it may be beneficial to further combine this hPSC-LV-CM differentiation approach with previously described maturation regimes.

STAR★METHODS

Detailed methods are provided in the online version of this paper and include the following:

- KEY RESOURCES TABLE
- RESOURCE AVAILABILITY
  - Lead contact
  - Materials availability
  - Data and code availability
- EXPERIMENTAL MODEL AND SUBJECT DETAILS
  - Human pluripotent stem cell lines' origin, characterization and maintenance
  - Donor heart procurement
- METHOD DETAILS
  - Directed differentiation of human pluripotent stem cell lines toward cardiomyocytes
  - Cardiomyocyte replating
  - Engineered heart tissues
  - Human adult heart tissue and isolated cardiomyocytes
  - Immunohistochemistry
  - Antibody table
  - Image analysis
  - Aldefluor assay
  - Flow cytometry
  - Western blot
  - RNA extraction, cDNA synthesis and RT-qPCR
  - Primer table
  - Transmission electron microscopy
  - MicroCT/X-ray microscope imaging
  - Mitochondrial respiration
  - Optical mapping
  - Intracellular (sharp electrode) electrophysiology
- QUANTIFICATION AND STATISTICAL ANALYSIS
  - Non-RNA-seq data
  - RNA-seq read alignment and quantification
  - Data exploration
  - Differential expression
  - GO enrichment analysis
  - Gene expression heatmaps

SUPPLEMENTAL INFORMATION

Supplemental information can be found online at https://doi.org/10.1016/j.crmeth.2023.100456.

ACKNOWLEDGMENTS

We thank the Smith and Turner labs (The Francis Crick Institute, UK) and the Harding and Gorelik labs (Imperial College London, UK) for experimental advice and/or critical comments on data. We especially thank Thusharika Kodagoda and Prag Pandey (Harding lab). We thank the following scientific

platforms/units at the Francis Crick Institute: advanced sequencing, light microscopy, the human embryo and stem cell unit, and research illustration and graphics. We thank Lyn Healy for advice and support with stem cells. We thank James Turner, Luis Miguel Monteiro, Rifdat Aoidi, Godfrey Smith, and Jose Sanchez Alonso-Mardones for advice. We thank James Turner and Alla Aharonov for critical manuscript reading. We thank the Francis Crick Institute translation team, especially Ranmali Nawaratne and Veronique Birault, for support and advice on the translation value of the work. This work was supported by grants from the British Heart Foundation (BHF-FS/12/37/29516 and RM/17/1/33377), the Wellcome Trust (210987/Z/18/Z), the MRC (MR/X50287X/1 and MR/R017050/01), and LifeArc (LifeArc-Crick Translation Fund). We were also funded by the Francis Crick Institute, which receives its core funding from Cancer Research UK (FC001157), the UK Medical Research Council (FC001157), and the Wellcome Trust (FC001157).

AUTHOR CONTRIBUTIONS

Conceptualization, A.S.B.; methodology, A.S.B. and N.D.; investigation, A.S.B., N.D., M.-V.C., T.J.O., L.I.T., E.F., A.J.F., S.T., C.B., A.W., and E.E.; formal analysis, A.S.B., R.M., N.D., M.-V.C., T.J.O., L.I.T., E.F., and A.J.F.; validation, A.S.B., N.D., M.-V.C., T.J.O., L.I.T., E.F., A.J.F., and S.T.; visualization, A.S.B., R.M., L.I.T., and N.D.; data curation, R.M.; software, R.M.; project administration, A.S.B.; writing – original draft, A.S.B.; writing – review & editing, A.S.B., N.D., M.-V.C., L.I.T., A.J.F., L.C., N.A.-G., E.E., K.T.M., R.M., S.E.H., and J.C.S.; funding acquisition, A.S.B. and J.C.S.; resources, A.S.B., N.A.-G., S.E.H., and J.C.S.; supervision, A.S.B., L.C., P.E.M., K.T.M., S.E.H., and J.C.S.

DECLARATION OF INTERESTS

N.A.-G. is the vice president of research and development at AnaBios, San Diego. P.E.M. is a founding partner and the chief corporate development officer of AnaBios, San Diego. The Francis Crick Institute has filed a patent application related to this work (WO 2020/245612), and A.S.B. is listed as an inventor. The Francis Crick Institute has granted an exclusive license to Axol Bioscience to commercialize the protocol for the generation and sale of cardiomyocytes for R&D and the provision of contract research services. N.D., C.B., J.C.S., and A.S.B. may benefit from this license.

Received: July 25, 2022
Revised: January 23, 2023
Accepted: March 25, 2023
Published: April 24, 2023

REFERENCES

1. Takasuna, K., Asakura, K., Araki, S., Ando, H., Kazusa, K., Kitaguchi, T., Kunimatsu, T., Suzuki, S., and Miyamoto, N. (2017). Comprehensive in vitro cardiac safety assessment using human stem cell technology: overview of CSAHi HEART initiative. J. Pharmacol. Toxicol. Methods *83*, 42–54. https://doi.org/10.1016/j.vascn.2016.09.004.
2. Yang, X., and Papoian, T. (2018). Moving beyond the comprehensive in vitro proarrhythmia assay: use of human-induced pluripotent stem cell-derived cardiomyocytes to assess contractile effects associated with drug-induced structural cardiotoxicity. J. Appl. Toxicol. *38*, 1166–1176. https://doi.org/10.1002/jat.3611.
3. Soma, Y., Morita, Y., Kishino, Y., Kanazawa, H., Fukuda, K., and Tohyama, S. (2021). The present state and future perspectives of cardiac regenerative therapy using human pluripotent stem cells. Front. Cardiovasc. Med. *8*, 774389. https://doi.org/10.3389/fcvm.2021.774389.
4. Burridge, P.W., Matsa, E., Shukla, P., Lin, Z.C., Churko, J.M., Ebert, A.D., Lan, F., Diecke, S., Huber, B., Mordwinkin, N.M., et al. (2014). Chemically defined generation of human cardiomyocytes. Nat. Methods *11*, 855–860. https://doi.org/10.1038/nmeth.2999.
5. Lian, X., Zhang, J., Azarin, S.M., Zhu, K., Hazeltine, L.B., Bao, X., Hsiao, C., Kamp, T.J., and Palecek, S.P. (2013). Directed cardiomyocyte differentiation from human pluripotent stem cells by modulating Wnt/beta-catenin signaling under fully defined conditions. Nat. Protoc. *8*, 162–175. https://doi.org/10.1038/nprot.2012.150.
6. Cyganek, L., Tiburcy, M., Sekeres, K., Gerstenberg, K., Bohnenberger, H., Lenz, C., Henze, S., Stauske, M., Salinas, G., Zimmermann, W.H., et al. (2018). Deep phenotyping of human induced pluripotent stem cell-derived atrial and ventricular cardiomyocytes. JCI Insight *3*, e99941. https://doi.org/10.1172/jci.insight.99941.
7. Yonemizu, S., Masuda, K., Kurata, Y., Notsu, T., Higashi, Y., Fukumura, K., Li, P., Ninomiya, H., Miake, J., Tsuneto, M., et al. (2019). Inhibitory effects of class I antiarrhythmic agents on Na(+) and Ca(2+) currents of human iPS cell-derived cardiomyocytes. Regen. Ther. *10*, 104–111. https://doi.org/10.1016/j.reth.2018.12.002.
8. Lee, J.H., Protze, S.I., Laksman, Z., Backx, P.H., and Keller, G.M. (2017). Human pluripotent stem cell-derived atrial and ventricular cardiomyocytes develop from distinct mesoderm populations. Cell Stem Cell *21*, 179–194.e4. https://doi.org/10.1016/j.stem.2017.07.003.
9. Feric, N.T., and Radisic, M. (2016). Maturing human pluripotent stem cell-derived cardiomyocytes in human engineered cardiac tissues. Adv. Drug Deliv. Rev. *96*, 110–134. https://doi.org/10.1016/j.addr.2015.04.019.
10. Jiang, Y., Park, P., Hong, S.M., and Ban, K. (2018). Maturation of cardiomyocytes derived from human pluripotent stem cells: current strategies and limitations. Mol. Cells *41*, 613–621. https://doi.org/10.14348/molcells.2018.0143.
11. Brade, T., Pane, L.S., Moretti, A., Chien, K.R., and Laugwitz, K.L. (2013). Embryonic heart progenitors and cardiogenesis. Cold Spring Harb. Perspect. Med. *3*, a013847. https://doi.org/10.1101/cshperspect.a013847.
12. Lescroart, F., Chabab, S., Lin, X., Rulands, S., Paulissen, C., Rodolosse, A., Auer, H., Achouri, Y., Dubois, C., Bondue, A., et al. (2014). Early lineage restriction in temporally distinct populations of Mesp1 progenitors during mammalian heart development. Nat. Cell Biol. *16*, 829–840. https://doi.org/10.1038/ncb3024.
13. Wei, Y., and Mikawa, T. (2000). Fate diversity of primitive streak cells during heart field formation in ovo. Dev. Dynam. *219*, 505–513. https://doi.org/10.1002/1097-0177(2000)9999:9999<::AID-DVDY1076>3.0.CO;2-6.
14. Bardot, E., Calderon, D., Santoriello, F., Han, S., Cheung, K., Jadhav, B., Burtscher, I., Artap, S., Jain, R., Epstein, J., et al. (2017). Foxa2 identifies a cardiac progenitor population with ventricular differentiation potential. Nat. Commun. *8*, 14428. https://doi.org/10.1038/ncomms14428.
15. Ivanovitch, K., Soro-Barrio, P., Chakravarty, P., Jones, R.A., Bell, D.M., Mousavy Gharavy, S.N., Stamataki, D., Delile, J., Smith, J.C., and Briscoe, J. (2021). Ventricular, atrial, and outflow tract heart progenitors arise from spatially and molecularly distinct regions of the primitive streak. PLoS Biol. *19*, e3001200. https://doi.org/10.1371/journal.pbio.3001200.
16. Rosenthal, N., and Xavier-Neto, J. (2000). From the bottom of the heart: anteroposterior decisions in cardiac muscle differentiation. Curr. Opin. Cell Biol. *12*, 742–746. https://doi.org/10.1016/s0955-0674(00)00162-9.
17. Moss, J.B., Xavier-Neto, J., Shapiro, M.D., Nayeem, S.M., McCaffery, P., Dräger, U.C., and Rosenthal, N. (1998). Dynamic patterns of retinoic acid synthesis and response in the developing mammalian heart. Dev. Biol. *199*, 55–71. https://doi.org/10.1006/dbio.1998.8911.
18. Hochgreb, T., Linhares, V.L., Menezes, D.C., Sampaio, A.C., Yan, C.Y.I., Cardoso, W.V., Rosenthal, N., and Xavier-Neto, J. (2003). A caudorostral wave of RALDH2 conveys anteroposterior information to the cardiac field. Development *130*, 5363–5374. https://doi.org/10.1242/dev.00750.
19. Xavier-Neto, J., Neville, C.M., Shapiro, M.D., Houghton, L., Wang, G.F., Nikovits, W., Jr., Stockdale, F.E., and Rosenthal, N. (1999). A retinoic acid-inducible transgenic marker of sino-atrial development in the mouse heart. Development *126*, 2677–2687. https://doi.org/10.1242/dev.126.12.2677.
20. Molina, C.E., Heijman, J., and Dobrev, D. (2016). Differences in left versus right ventricular electrophysiological properties in cardiac dysfunction

and arrhythmogenesis. Arrhythmia Electrophysiol. Rev. *5*, 14–19. https://doi.org/10.15420/aer.2016.8.2.

21. Adrogue, J.V., Sharma, S., Ngumbela, K., Essop, M.F., and Taegtmeyer, H. (2005). Acclimatization to chronic hypobaric hypoxia is associated with a differential transcriptional profile between the right and left ventricle. Mol. Cell. Biochem. *278*, 71–78. https://doi.org/10.1007/s11010-005-6629-5.

22. Später, D., Abramczuk, M.K., Buac, K., Zangi, L., Stachel, M.W., Clarke, J., Sahara, M., Ludwig, A., and Chien, K.R. (2013). A HCN4+ cardiomyogenic progenitor derived from the first heart field and human pluripotent stem cells. Nat. Cell Biol. *15*, 1098–1106. https://doi.org/10.1038/ncb2824.

23. DeLaughter, D.M., Bick, A.G., Wakimoto, H., McKean, D., Gorham, J.M., Kathiriya, I.S., Hinson, J.T., Homsy, J., Gray, J., Pu, W., et al. (2016). Single-cell resolution of temporal gene expression during heart development. Dev. Cell *39*, 480–490. https://doi.org/10.1016/j.devcel.2016.10.001.

24. Bernardo, A.S., Faial, T., Gardner, L., Niakan, K.K., Ortmann, D., Senner, C.E., Callery, E.M., Trotter, M.W., Hemberger, M., Smith, J.C., et al. (2011). BRACHYURY and CDX2 mediate BMP-induced differentiation of human and mouse pluripotent stem cells into embryonic and extraembryonic lineages. Cell Stem Cell *9*, 144–155. https://doi.org/10.1016/j.stem.2011.06.015.

25. Marom, K., Fainsod, A., and Steinbeisser, H. (1999). Patterning of the mesoderm involves several threshold responses to BMP-4 and Xwnt-8. Mech. Dev. *87*, 33–44. https://doi.org/10.1016/s0925-4773(99)00137-9.

26. Hoppler, S., and Moon, R.T. (1998). BMP-2/-4 and Wnt-8 cooperatively pattern the Xenopus mesoderm. Mech. Dev. *71*, 119–129. https://doi.org/10.1016/s0925-4773(98)00004-5.

27. Chhabra, S., Liu, L., Goh, R., Kong, X., and Warmflash, A. (2019). Dissecting the dynamics of signaling events in the BMP, WNT, and NODAL cascade during self-organized fate patterning in human gastruloids. PLoS Biol. *17*, e3000498. https://doi.org/10.1371/journal.pbio.3000498.

28. Cui, Y., Zheng, Y., Liu, X., Yan, L., Fan, X., Yong, J., Hu, Y., Dong, J., Li, Q., Wu, X., et al. (2019). Single-cell transcriptome analysis maps the developmental track of the human heart. Cell Rep. *26*, 1934–1950.e5. https://doi.org/10.1016/j.celrep.2019.01.079.

29. McFadden, D.G., Barbosa, A.C., Richardson, J.A., Schneider, M.D., Srivastava, D., and Olson, E.N. (2005). The Hand1 and Hand2 transcription factors regulate expansion of the embryonic cardiac ventricles in a gene dosage-dependent manner. Development *132*, 189–201. https://doi.org/10.1242/dev.01562.

30. Sheikh, F., Lyon, R.C., and Chen, J. (2015). Functions of myosin light chain-2 (MYL2) in cardiac muscle and disease. Gene *569*, 14–20. https://doi.org/10.1016/j.gene.2015.06.027.

31. Wu, S.P., Cheng, C.M., Lanz, R.B., Wang, T., Respress, J.L., Ather, S., Chen, W., Tsai, S.J., Wehrens, X.H.T., Tsai, M.J., and Tsai, S.Y. (2013). Atrial identity is determined by a COUP-TFII regulatory network. Dev. Cell *25*, 417–426. https://doi.org/10.1016/j.devcel.2013.04.017.

32. Kuppusamy, K.T., Jones, D.C., Sperber, H., Madan, A., Fischer, K.A., Rodriguez, M.L., Pabon, L., Zhu, W.Z., Tulloch, N.L., Yang, X., et al. (2015). Let-7 family of microRNA is required for maturation and adult-like metabolism in stem cell-derived cardiomyocytes. Proc. Natl. Acad. Sci. USA *112*, E2785–E2794. https://doi.org/10.1073/pnas.1424042112.

33. Giacomelli, E., Meraviglia, V., Campostrini, G., Cochrane, A., Cao, X., van Helden, R.W.J., Krotenberg Garcia, A., Mircea, M., Kostidis, S., Davis, R.P., et al. (2020). Human-iPSC-Derived cardiac stromal cells enhance maturation in 3D cardiac microtissues and reveal non-cardiomyocyte contributions to heart disease. Cell Stem Cell *26*, 862–879.e11. https://doi.org/10.1016/j.stem.2020.05.004.

34. Branco, M.A., Cotovio, J.P., Rodrigues, C.A.V., Vaz, S.H., Fernandes, T.G., Moreira, L.M., Cabral, J.M.S., and Diogo, M.M. (2019). Transcriptomic analysis of 3D cardiac differentiation of human induced pluripotent stem cells reveals faster cardiomyocyte maturation compared to 2D culture. Sci. Rep. *9*, 9229. https://doi.org/10.1038/s41598-019-45047-9.

35. Tucker, N.R., Chaffin, M., Fleming, S.J., Hall, A.W., Parsons, V.A., Bedi, K.C., Jr., Akkad, A.D., Herndon, C.N., Arduini, A., Papangeli, I., et al. (2020). Transcriptional and cellular diversity of the human heart. Circulation *142*, 466–482. https://doi.org/10.1161/CIRCULATIONAHA.119.045401.

36. Xin, M., Small, E.M., van Rooij, E., Qi, X., Richardson, J.A., Srivastava, D., Nakagawa, O., and Olson, E.N. (2007). Essential roles of the bHLH transcription factor Hrt2 in repression of atrial gene expression and maintenance of postnatal cardiac function. Proc. Natl. Acad. Sci. USA *104*, 7975–7980. https://doi.org/10.1073/pnas.0702447104.

37. Bao, Z.Z., Bruneau, B.G., Seidman, J.G., Seidman, C.E., and Cepko, C.L. (1999). Regulation of chamber-specific gene expression in the developing heart by Irx4. Science *283*, 1161–1164. https://doi.org/10.1126/science.283.5405.1161.

38. Bruneau, B.G., Logan, M., Davis, N., Levi, T., Tabin, C.J., Seidman, J.G., and Seidman, C.E. (1999). Chamber-specific cardiac expression of Tbx5 and heart defects in Holt-Oram syndrome. Dev. Biol. *211*, 100–108. https://doi.org/10.1006/dbio.1999.9298.

39. Mahmoud, A.I., Kocabas, F., Muralidhar, S.A., Kimura, W., Koura, A.S., Thet, S., Porrello, E.R., and Sadek, H.A. (2013). Meis1 regulates postnatal cardiomyocyte cell cycle arrest. Nature *497*, 249–253. https://doi.org/10.1038/nature12054.

40. Crocini, C., and Gotthardt, M. (2021). Cardiac sarcomere mechanics in health and disease. Biophys. Rev. *13*, 637–652. https://doi.org/10.1007/s12551-021-00840-7.

41. Valle, G., Faulkner, G., De Antoni, A., Pacchioni, B., Pallavicini, A., Pandolfo, D., Tiso, N., Toppo, S., Trevisan, S., and Lanfranchi, G. (1997). Telethonin, a novel sarcomeric protein of heart and skeletal muscle. FEBS Lett. *415*, 163–168. https://doi.org/10.1016/s0014-5793(97)01108-3.

42. Givvimani, S., Kundu, S., Narayanan, N., Armaghan, F., Qipshidze, N., Pushpakumar, S., Vacek, T.P., and Tyagi, S.C. (2013). TIMP-2 mutant decreases MMP-2 activity and augments pressure overload induced LV dysfunction and heart failure. Arch. Physiol. Biochem. *119*, 65–74. https://doi.org/10.3109/13813455.2012.755548.

43. Puthanveetil, P., Wan, A., and Rodrigues, B. (2013). FoxO1 is crucial for sustaining cardiomyocyte metabolism and cell survival. Cardiovasc. Res. *97*, 393–403. https://doi.org/10.1093/cvr/cvs426.

44. Ferreira, J.C.B., Mori, M.A., and Gross, E.R. (2019). Mitochondrial bioenergetics and quality control mechanisms in health and disease. Oxid. Med. Cell. Longev. *2019*, 5406751. https://doi.org/10.1155/2019/5406751.

45. Cagalinec, M., Zahradníková, A., Zahradníková, A., Jr., Kováčová, D., Paulis, L., Kureková, S., Hot'ka, M., Pavelková, J., Plaas, M., Novotová, M., and Zahradník, I. (2019). Calcium signaling and contractility in cardiac myocyte of wolframin deficient rats. Front. Physiol. *10*, 172. https://doi.org/10.3389/fphys.2019.00172.

46. Rao, J., Pfeiffer, M.J., Frank, S., Adachi, K., Piccini, I., Quaranta, R., Araúzo-Bravo, M., Schwarz, J., Schade, D., Leidel, S., et al. (2016). Stepwise clearance of repressive roadblocks drives cardiac induction in human ESCs. Cell Stem Cell *18*, 341–353. https://doi.org/10.1016/j.stem.2015.11.019.

47. Zhang, Q., Carlin, D., Zhu, F., Cattaneo, P., Ideker, T., Evans, S.M., Bloomekatz, J., and Chi, N.C. (2021). Unveiling complexity and multipotentiality of early heart fields. Circ. Res. *129*, 474–487. https://doi.org/10.1161/CIRCRESAHA.121.318943.

48. Zhang, J.Z., Termglinchan, V., Shao, N.Y., Itzhaki, I., Liu, C., Ma, N., Tian, L., Wang, V.Y., Chang, A.C.Y., Guo, H., et al. (2019). A human iPSC double-reporter system enables purification of cardiac lineage subpopulations with distinct function and drug response profiles. Cell Stem Cell *24*, 802–811.e5. https://doi.org/10.1016/j.stem.2019.02.015.

CellPress
OPEN ACCESS

49. Ahmed, R.E., Anzai, T., Chanthra, N., and Uosaki, H. (2020). A brief review of current maturation methods for human induced pluripotent stem cells-derived cardiomyocytes. Front. Cell Dev. Biol. *8*, 178. https://doi.org/10.3389/fcell.2020.00178.

50. Garbern, J.C., and Lee, R.T. (2021). Mitochondria and metabolic transitions in cardiomyocytes: lessons from development for stem cell-derived cardiomyocytes. Stem Cell Res. Ther. *12*, 177. https://doi.org/10.1186/s13287-021-02252-6.

51. Eisner, V., Cupo, R.R., Gao, E., Csordás, G., Slovinsky, W.S., Paillard, M., Cheng, L., Ibetti, J., Chen, S.R.W., Chuprun, J.K., et al. (2017). Mitochondrial fusion dynamics is robust in the heart and depends on calcium oscillations and contractile activity. Proc. Natl. Acad. Sci. USA *114*, E859–E868. https://doi.org/10.1073/pnas.1617288114.

52. Ehler, E. (2016). Cardiac cytoarchitecture - why the "hardware" is important for heart function. Biochim. Biophys. Acta *1863*, 1857–1863. https://doi.org/10.1016/j.bbamcr.2015.11.006.

53. Frank, D., and Frey, N. (2011). Cardiac Z-disc signaling network. J. Biol. Chem. *286*, 9897–9904. https://doi.org/10.1074/jbc.R110.174268.

54. Zhao, M.T., Ye, S., Su, J., and Garg, V. (2020). Cardiomyocyte proliferation and maturation: two sides of the same coin for heart regeneration. Front. Cell Dev. Biol. *8*, 594226. https://doi.org/10.3389/fcell.2020.594226.

55. Satin, J., Kehat, I., Caspi, O., Huber, I., Arbel, G., Itzhaki, I., Magyar, J., Schroder, E.A., Perlman, I., and Gepstein, L. (2004). Mechanism of spontaneous excitability in human embryonic stem cell derived cardiomyocytes. J. Physiol. *559*, 479–496. https://doi.org/10.1113/jphysiol.2004.068213.

56. Baumgartner, S., Halbach, M., Krausgrill, B., Maass, M., Srinivasan, S.P., Sahito, R.G.A., Peinkofer, G., Nguemo, F., Müller-Ehmsen, J., and Hescheler, J. (2015). Electrophysiological and morphological maturation of murine fetal cardiomyocytes during electrical stimulation in vitro. J. Cardiovasc. Pharmacol. Therapeut. *20*, 104–112. https://doi.org/10.1177/1074248414536273.

57. Kane, C., and Terracciano, C.M.N. (2017). Concise review: criteria for chamber-specific categorization of human cardiac myocytes derived from pluripotent stem cells. Stem Cell. *35*, 1881–1897. https://doi.org/10.1002/stem.2649.

58. Lanner, J.T., Georgiou, D.K., Joshi, A.D., and Hamilton, S.L. (2010). Ryanodine receptors: structure, expression, molecular details, and function in calcium release. Cold Spring Harbor Perspect. Biol. *2*, a003996. https://doi.org/10.1101/cshperspect.a003996.

59. Nostro, M.C., Cheng, X., Keller, G.M., and Gadue, P. (2008). Wnt, activin, and BMP signaling regulate distinct stages in the developmental pathway from embryonic stem cells to blood. Cell Stem Cell *2*, 60–71. https://doi.org/10.1016/j.stem.2007.10.011.

60. Devalla, H.D., Schwach, V., Ford, J.W., Milnes, J.T., El-Haou, S., Jackson, C., Gkatzis, K., Elliott, D.A., Chuva de Sousa Lopes, S.M., Mummery, C.L., et al. (2015). Atrial-like cardiomyocytes from human pluripotent stem cells are a robust preclinical model for assessing atrial-selective pharmacology. EMBO Mol. Med. *7*, 394–410. https://doi.org/10.15252/emmm.201404757.

61. Zhang, Q., Jiang, J., Han, P., Yuan, Q., Zhang, J., Zhang, X., Xu, Y., Cao, H., Meng, Q., Chen, L., et al. (2011). Direct differentiation of atrial and ventricular myocytes from human embryonic stem cells by alternating retinoid signals. Cell Res. *21*, 579–587. https://doi.org/10.1038/cr.2010.163.

62. Zhang, X., Cao, H., Bai, S., Huo, W., and Ma, Y. (2017). Differentiation and characterization of rhesus monkey atrial and ventricular cardiomyocytes from induced pluripotent stem cells. Stem Cell Res. *20*, 21–29. https://doi.org/10.1016/j.scr.2017.02.002.

63. Yang, D., Gomez-Garcia, J., Funakoshi, S., Tran, T., Fernandes, I., Bader, G.D., Laflamme, M.A., and Keller, G.M. (2022). Modeling human multi-lineage heart field development with pluripotent stem cells. Cell Stem Cell *29*, 1382–1401.e8. https://doi.org/10.1016/j.stem.2022.08.007.

64. Wiesinger, A., Boink, G.J.J., Christoffels, V.M., and Devalla, H.D. (2021). Retinoic acid signaling in heart development: application in the differentiation of cardiovascular lineages from human pluripotent stem cells. Stem Cell Rep. *16*, 2589–2606. https://doi.org/10.1016/j.stemcr.2021.09.010.

65. Cai, C.L., Liang, X., Shi, Y., Chu, P.H., Pfaff, S.L., Chen, J., and Evans, S. (2003). Isl1 identifies a cardiac progenitor population that proliferates prior to differentiation and contributes a majority of cells to the heart. Dev. Cell *5*, 877–889. https://doi.org/10.1016/s1534-5807(03)00363-0.

66. Tyser, R.C.V., Mahammadov, E., Nakanoh, S., Vallier, L., Scialdone, A., and Srinivas, S. (2021). Single-cell transcriptomic characterization of a gastrulating human embryo. Nature *600*, 285–289. https://doi.org/10.1038/s41586-021-04158-y.

67. Feyen, D.A.M., McKeithan, W.L., Bruyneel, A.A.N., Spiering, S., Hörmann, L., Ulmer, B., Zhang, H., Briganti, F., Schweizer, M., Hegyi, B., et al. (2020). Metabolic maturation media improve physiological function of human iPSC-derived cardiomyocytes. Cell Rep. *32*, 107925. https://doi.org/10.1016/j.celrep.2020.107925.

68. Yang, X., Rodriguez, M.L., Leonard, A., Sun, L., Fischer, K.A., Wang, Y., Ritterhoff, J., Zhao, L., Kolwicz, S.C., Jr., Pabon, L., et al. (2019). Fatty acids enhance the maturation of cardiomyocytes derived from human pluripotent stem cells. Stem Cell Rep. *13*, 657–668. https://doi.org/10.1016/j.stemcr.2019.08.013.

69. Lundy, S.D., Zhu, W.Z., Regnier, M., and Laflamme, M.A. (2013). Structural and functional maturation of cardiomyocytes derived from human pluripotent stem cells. Stem Cell. Dev. *22*, 1991–2002. https://doi.org/10.1089/scd.2012.0490.

70. Ackers-Johnson, M., Li, P.Y., Holmes, A.P., O'Brien, S.M., Pavlovic, D., and Foo, R.S. (2016). A simplified, langendorff-free method for concomitant isolation of viable cardiac myocytes and nonmyocytes from the adult mouse heart. Circ. Res. *119*, 909–920. https://doi.org/10.1161/CIRCRESAHA.116.309202.

71. Kabaeva, Z., Zhao, M., and Michele, D.E. (2008). Blebbistatin extends culture life of adult mouse cardiac myocytes and allows efficient and stable transgene expression. Am. J. Physiol. Heart Circ. Physiol. *294*, H1667–H1674. https://doi.org/10.1152/ajpheart.01144.2007.

72. Banyasz, T., Lozinskiy, I., Payne, C.E., Edelmann, S., Norton, B., Chen, B., Chen-Izu, Y., Izu, L.T., and Balke, C.W. (2008). Transformation of adult rat cardiac myocytes in primary culture. Exp. Physiol. *93*, 370–382. https://doi.org/10.1113/expphysiol.2007.040659.

73. Mannhardt, I., Breckwoldt, K., Letuffe-Brenière, D., Schaaf, S., Schulz, H., Neuber, C., Benzin, A., Werner, T., Eder, A., Schulze, T., et al. (2016). Human engineered heart tissue: analysis of contractile force. Stem Cell Rep. *7*, 29–42. https://doi.org/10.1016/j.stemcr.2016.04.011.

74. Abilez, O.J., Tzatzalos, E., Yang, H., Zhao, M.T., Jung, G., Zöllner, A.M., Tiburcy, M., Riegler, J., Matsa, E., Shukla, P., et al. (2018). Passive stretch induces structural and functional maturation of engineered heart muscle as predicted by computational modeling. Stem Cell. *36*, 265–277. https://doi.org/10.1002/stem.2732.

75. Nunes, S.S., Miklas, J.W., Liu, J., Aschar-Sobbi, R., Xiao, Y., Zhang, B., Jiang, J., Massé, S., Gagliardi, M., Hsieh, A., et al. (2013). Biowire: a platform for maturation of human pluripotent stem cell-derived cardiomyocytes. Nat. Methods *10*, 781–787. https://doi.org/10.1038/nmeth.2524.

76. Ronaldson-Bouchard, K., Ma, S.P., Yeager, K., Chen, T., Song, L., Sirabella, D., Morikawa, K., Teles, D., Yazawa, M., and Vunjak-Novakovic, G. (2018). Advanced maturation of human cardiac tissue grown from pluripotent stem cells. Nature *556*, 239–243. https://doi.org/10.1038/s41586-018-0016-3.

77. Lemoine, M.D., Mannhardt, I., Breckwoldt, K., Prondzynski, M., Flenner, F., Ulmer, B., Hirt, M.N., Neuber, C., Horváth, A., Kloth, B., et al. (2017). Human iPSC-derived cardiomyocytes cultured in 3D engineered heart

tissue show physiological upstroke velocity and sodium current density. Sci. Rep. *7*, 5464. https://doi.org/10.1038/s41598-017-05600-w.

78. Horváth, A., Lemoine, M.D., Löser, A., Mannhardt, I., Flenner, F., Uzun, A.U., Neuber, C., Breckwoldt, K., Hansen, A., Girdauskas, E., et al. (2018). Low resting membrane potential and low inward rectifier potassium currents are not inherent features of hiPSC-derived cardiomyocytes. Stem Cell Rep. *10*, 822–833. https://doi.org/10.1016/j.stemcr.2018.01.012.

79. Ulmer, B.M., Stoehr, A., Schulze, M.L., Patel, S., Gucek, M., Mannhardt, I., Funcke, S., Murphy, E., Eschenhagen, T., and Hansen, A. (2018). Contractile work contributes to maturation of energy metabolism in hiPSC-derived cardiomyocytes. Stem Cell Rep. *10*, 834–847. https://doi.org/10.1016/j.stemcr.2018.01.039.

80. Goversen, B., van der Heyden, M.A.G., van Veen, T.A.B., and de Boer, T.P. (2018). The immature electrophysiological phenotype of iPSC-CMs still hampers in vitro drug screening: special focus on Ik1. Pharmacol. Ther. *183*, 127–136. https://doi.org/10.1016/j.pharmthera.2017.10.001.

81. Jonsson, M.K.B., Vos, M.A., Mirams, G.R., Duker, G., Sartipy, P., de Boer, T.P., and van Veen, T.A.B. (2012). Application of human stem cell-derived cardiomyocytes in safety pharmacology requires caution beyond hERG. J. Mol. Cell. Cardiol. *52*, 998–1008. https://doi.org/10.1016/j.yjmcc.2012.02.002.

82. Chong, J.J.H., Yang, X., Don, C.W., Minami, E., Liu, Y.W., Weyers, J.J., Mahoney, W.M., Van Biber, B., Cook, S.M., Palpant, N.J., et al. (2014). Human embryonic-stem-cell-derived cardiomyocytes regenerate non-human primate hearts. Nature *510*, 273–277. https://doi.org/10.1038/nature13233.

83. Shiba, Y., Gomibuchi, T., Seto, T., Wada, Y., Ichimura, H., Tanaka, Y., Ogasawara, T., Okada, K., Shiba, N., Sakamoto, K., et al. (2016). Allogeneic transplantation of iPS cell-derived cardiomyocytes regenerates primate hearts. Nature *538*, 388–391. https://doi.org/10.1038/nature19815.

84. Liu, Y.W., Chen, B., Yang, X., Fugate, J.A., Kalucki, F.A., Futakuchi-Tsuchida, A., Couture, L., Vogel, K.W., Astley, C.A., Baldessari, A., et al. (2018). Human embryonic stem cell-derived cardiomyocytes restore function in infarcted hearts of non-human primates. Nat. Biotechnol. *36*, 597–605. https://doi.org/10.1038/nbt.4162.

85. Romagnuolo, R., Masoudpour, H., Porta-Sánchez, A., Qiang, B., Barry, J., Laskary, A., Qi, X., Massé, S., Magtibay, K., Kawajiri, H., et al. (2019). Human embryonic stem cell-derived cardiomyocytes regenerate the infarcted pig heart but induce ventricular tachyarrhythmias. Stem Cell Rep. *12*, 967–981. https://doi.org/10.1016/j.stemcr.2019.04.005.

86. Groenewegen, A., Rutten, F.H., Mosterd, A., and Hoes, A.W. (2020). Epidemiology of heart failure. Eur. J. Heart Fail. *22*, 1342–1356. https://doi.org/10.1002/ejhf.1858.

87. Gajjela, H., Kela, I., Kakarala, C.L., Hassan, M., Belavadi, R., Gudigopuram, S.V.R., Raguthu, C.C., Modi, S., and Sange, I. (2021). Milestones in heart failure: how far we have come and how far we have left to Go. Cureus *13*, e20359. https://doi.org/10.7759/cureus.20359.

88. Yang, K.C., Yamada, K.A., Patel, A.Y., Topkara, V.K., George, I., Cheema, F.H., Ewald, G.A., Mann, D.L., and Nerbonne, J.M. (2014). Deep RNA sequencing reveals dynamic regulation of myocardial noncoding RNAs in failing human heart and remodeling with mechanical circulatory support. Circulation *129*, 1009–1021. https://doi.org/10.1161/CIRCULATIONAHA.113.003863.

89. Schindelin, J., Arganda-Carreras, I., Frise, E., Kaynig, V., Longair, M., Pietzsch, T., Preibisch, S., Rueden, C., Saalfeld, S., Schmid, B., et al. (2012). Fiji: an open-source platform for biological-image analysis. Nat Methods *9*, 676–682. https://doi.org/10.1038/nmeth.2019.

90. Sala, L., van Meer, B.J., Tertoolen, L.G.J., Bakkers, J., Bellin, M., Davis, R.P., Denning, C., Dieben, M.A.E., Eschenhagen, T., Giacomelli, E., et al. (2018). MUSCLEMOTION: a versatile open software tool to quantify cardiomyocyte and cardiac muscle contraction in vitro and in vivo. Circ. Res. *122*, e5–e16. https://doi.org/10.1161/CIRCRESAHA.117.312067.

91. Ewels, P.A., Peltzer, A., Fillinger, S., Patel, H., Alneberg, J., Wilm, A., Garcia, M.U., Di Tommaso, P., and Nahnsen, S. (2020). The nf-core framework for community-curated bioinformatics pipelines. Nat. Biotechnol. *38*, 276–278. https://doi.org/10.1038/s41587-020-0439-x.

92. Dobin, A., Davis, C.A., Schlesinger, F., Drenkow, J., Zaleski, C., Jha, S., Batut, P., Chaisson, M., and Gingeras, T.R. (2013). STAR: ultrafast universal RNA-seq aligner. Bioinformatics *29*, 15–21. https://doi.org/10.1093/bioinformatics/bts635.

93. Li, B., and Dewey, C.N. (2011). RSEM: accurate transcript quantification from RNA-Seq data with or without a reference genome. BMC Bioinf. *12*, 323. https://doi.org/10.1186/1471-2105-12-323.

94. Love, M.I., Huber, W., and Anders, S. (2014). Moderated estimation of fold change and dispersion for RNA-seq data with DESeq2. Genome Biol. *15*, 550. https://doi.org/10.1186/s13059-014-0550-8.

95. Soneson, C., Love, M.I., and Robinson, M.D. (2015). Differential analyses for RNA-seq: transcript-level estimates improve gene-level inferences. F1000Res. *4*, 1521. https://doi.org/10.12688/f1000research.7563.2.

96. Blighe, K., and Lun, A. (2020). PCAtools: PCAtools: everything. Principal components analysis. R package version 2.0.0. https://github.com/kevinblighe/PCAtools.

97. Witten, D. (2019). PoiClaClu: classification and clustering of sequencing data based on a Poisson model. R package version 1.0.2.1. https://CRAN.R-project.org/package=PoiClaClu.

98. Ignatiadis, N., Klaus, B., Zaugg, J.B., and Huber, W. (2016). Data-driven hypothesis weighting increases detection power in genome-scale multiple testing. Nat. Methods *13*, 577–580. https://doi.org/10.1038/nmeth.3885.

99. Pantano, L. (2020). DEGreport: report of DEG analysis. R package version 1.24.0. http://lpantano.github.io/DEGreport/.

100. Yu, G., Wang, L.G., Han, Y., and He, Q.Y. (2012). clusterProfiler: an R package for comparing biological themes among gene clusters. OMICS *16*, 284–287. https://doi.org/10.1089/omi.2011.0118.

101. Page, G., Ratchada, P., Miron, Y., Steiner, G., Ghetti, A., Miller, P.E., Reynolds, J.A., Wang, K., Greiter-Wilke, A., Polonchuk, L., et al. (2016). Human ex-vivo action potential model for pro-arrhythmia risk assessment. J. Pharmacol. Toxicol. Methods *81*, 183–195. https://doi.org/10.1016/j.vascn.2016.05.016.

102. Hansen, A., Eder, A., Bönstrup, M., Flato, M., Mewe, M., Schaaf, S., Aksehirlioglu, B., Schwoerer, A.P., Uebeler, J., and Eschenhagen, T. (2010). Development of a drug screening platform based on engineered heart tissue. Circ. Res. *107*, 35–44. https://doi.org/10.1161/CIRCRESAHA.109.211458.

103. Abi-Gerges, N., Indersmitten, T., Truong, K., Nguyen, W., Ratchada, P., Nguyen, N., Page, G., Miller, P.E., and Ghetti, A. (2020). Multiparametric mechanistic profiling of inotropic drugs in adult human primary cardiomyocytes. Sci. Rep. *10*, 7692. https://doi.org/10.1038/s41598-020-64657-2.

104. Nguyen, N., Nguyen, W., Nguyenton, B., Ratchada, P., Page, G., Miller, P.E., Ghetti, A., and Abi-Gerges, N. (2017). Adult human primary cardiomyocyte-based model for the simultaneous prediction of drug-induced inotropic and pro-arrhythmia risk. Front. Physiol. *8*, 1073. https://doi.org/10.3389/fphys.2017.01073.

105. Ton, A.T., Nguyen, W., Sweat, K., Miron, Y., Hernandez, E., Wong, T., Geft, V., Macias, A., Espinoza, A., Truong, K., et al. (2021). Arrhythmogenic and antiarrhythmic actions of late sustained sodium current in the adult human heart. Sci. Rep. *11*, 12014. https://doi.org/10.1038/s41598-021-91528-1.

106. Messerli, J.M., Eppenberger-Eberhardt, M.E., Rutishauser, B.M., Schwarb, P., von Arx, P., Koch-Schneidemann, S., Eppenberger, H.M., and Perriard, J.C. (1993). Remodelling of cardiomyocyte cytoarchitecture visualized by three-dimensional (3D) confocal microscopy. Histochemistry *100*, 193–202. https://doi.org/10.1007/BF00269092.

107. Grove, B.K., Kurer, V., Lehner, C., Doetschman, T.C., Perriard, J.C., and Eppenberger, H.M. (1984). A new 185,000-dalton skeletal muscle protein detected by monoclonal antibodies. J. Cell Biol. *98*, 518–524. https://doi.org/10.1083/jcb.98.2.518.

108. Obermann, W.M., Gautel, M., Steiner, F., van der Ven, P.F., Weber, K., and Fürst, D.O. (1996). The structure of the sarcomeric M band: localization of defined domains of myomesin, M-protein, and the 250-kD carboxy-terminal region of titin by immunoelectron microscopy. J. Cell Biol. *134*, 1441–1453. https://doi.org/10.1083/jcb.134.6.1441.

109. Hortigon-Vinagre, M.P., Zamora, V., Burton, F.L., Green, J., Gintant, G.A., and Smith, G.L. (2016). The use of ratiometric fluorescence measurements of the voltage sensitive dye di-4-ANEPPS to examine action potential characteristics and drug effects on human induced pluripotent stem cell-derived cardiomyocytes. Toxicol. Sci. *154*, 320–331. https://doi.org/10.1093/toxsci/kfw171.

STAR★METHODS

KEY RESOURCES TABLE

REAGENT or RESOURCE	SOURCE	IDENTIFIER
Antibodies		
Antibodies for western blot, flow cytometry and immunofluorescence antibodies	Various	See antibody table (STAR Methods)
**rowheadBiological samples**		
Human adult RV tissue	AnaBios	200317HHA, 180828HHB, 181008HHA
Human adult LV tissue	AnaBios	200317HHA, 180828HHB, 181008HHA
Human adult RA tissue	AnaBios	200317HHA, 180828HHB, 181008HHA
Human adult LA tissue	AnaBios	200317HHA, 180828HHB, 181008HHA
Human adult RV-CMs	AnaBios	200814HHA, 201005HHA, 201030HHB
Human adult LV-CMs	AnaBios	200814HHA, 201005HHA, 201030HHB
Chemicals, peptides, and recombinant proteins		
Y-27632	Tocris	1254
CHIR99021	Sellek Chemicals	S2924
IWR-1	Sigma Aldrich	I0161-5MG
Lactate	Sigma Aldrich	L7022
Activin A	R&D	338-AC-010
FGF2	R&D	233-FB-025
BMP4	R&D	314-BP-010
L-ascorbic acid	Sigma Aldrich	A8960
AGN193109	Santa Cruz	sc-210768
Fibronectin	Sigma Aldrich	F1141-5MG
PFA	Thermo Fisher Scientific	28908
Agarose	Sigma Aldrich	A9539
Triton X-100	Sigma Aldrich	T8787
Donkey serum	Sigma Aldrich	D9663
Goat serum	Sigma Aldrich	G9023
BSA	Thermo Fisher Scientific	B4287
FBS	Thermo Fisher Scientific	16000044
TBS	Sigma Aldrich	T6664
DPBS	Thermo Fisher Scientific	14190–144
DAPI	Thermo Fisher Scientific	D1306
Hoechst	Thermo Fisher Scientific	33342
N-propylgallate	Sigma Aldrich	P3130
Glycerol	Thermo Fisher Scientific	A16205
Perm/Wash buffer	BD Biosciences	554723
Cell Lytic M buffer	Sigma Aldrich	C2978
SDS LB buffer	BIO-RAD	161–0416
Pre-cast Bis-Tris 4-20% gels	Genscript	M00657
Tri-Glycine 4–20% gel	NuSep	NG12-420
Tween 20	Sigma Aldrich	P1379
Odissey blocking solution	LI-COR	927–60001
Dimethyl sulfoxide	Sigma Aldrich	D2650
Na phosphate monobasic (PB)	EMS	21190
Glutaraldehyde	Sigma Aldrich	340855
CyGEL™	BioStatus	CY10500

(Continued on next page)

Continued		
REAGENT or RESOURCE	SOURCE	IDENTIFIER
Potassium ferricyanide	Sigma Aldrich	702587
Thiocarbohydrazide	Sigma Aldrich	223220
Osmium tetroxide	TAAB	O002
Uranyl acetate	AGAR Scientific	R1260A
Propylene oxide	AGAR Scientific	AGR1080
Durcupan resin single components A-D	Sigma Aldrich	44602, 44591, 44608, 44612
di-4-ANNEPS	Thermo Fisher Scientific	D1199
Fura-4F	Thermo Fisher Scientific	F14175
D-Galactose	Sigma Aldrich	G0750
Thapsigargin	Sigma Aldrich	T9033
Ryanodine	Sigma Aldrich	559276-500UG
JTV 519 fumarate	Tocris	4564
TrypLE Express	ThermoFisher Scientific	12604013
Versene	ThermoFisher Scientific	15040066
mTeSR1	STEMCELL Technologies	100–0276
RPMI 1640	STEMCELL Technologies	118750
B27-insulin	ThermoFisher Scientific	A1895601
B27	ThermoFisher Scientific	17504044
RPMI 1640 - glucose	STEMCELL Technologies	11875
B27 -vitamin A	ThermoFisher Scientific	12587010
GFR Matrigel	Corning	354230
Geltrex	Thermo Fisher Scientific	A1413301
Collagenase type II solution	Worthington	LS004176
Ca^{2+} free HBSS	Thermo Fisher Scientific	14175–053
HEPES	Thermo Fisher Scientific	15630080
N-benzyl-p-toluene sulfonamide	TCI	B3082
Ca^{2+} containing DMEM	Sigma Aldrich	F0415
DNase	Sigma Aldrich	D8764
Penicillin/streptomycin	Thermo Fisher Scientific	15070063
Fibrinogen	Sigma Aldrich	F4753
Thrombine	Biopur	BP11101104
Aprotinin	Sigma Aldrich	A1153
Horse serum	Thermo Fisher Scientific	26050
DMEM	Sigma Aldrich	D5671
Phenol-free/serum-free DMEM	Thermo Fisher Scientific	14430–01
10x DMEM	Thermo Fisher Scientific	52100–021
Sodium Pyruvate	Thermo Fisher Scientific	11360–070
Glutamax	Thermo Fisher Scientific	35050–038
Insulin	Sigma Aldrich	19278-5ML
Critical commercial assays		
Stemflow Human and Mouse Pluripotent Stem Cell Analysis Kit	BD Biosciences	560477
Aldefluor Kit	STEMCELL Technologies	01700
Cardiomyocyte dissociation kit	STEMCELL Technologies	05025
RNeasy mini kit	Qiagen	74104
Maxima First Strand cDNA Synthesis kit	Thermo Fisher Scientific	K1641
LightCycler 480 SYBR Green I Master mix	Roche Holding AG	04887352001
Seahorse XF flux pack	Agilent	102416–100
Seahorse XF DMEM assay medium pack	Agilent	103680–100
Cell Mito Stress kit	Agilent	103010–100

(*Continued on next page*)

Continued

REAGENT or RESOURCE	SOURCE	IDENTIFIER
Deposited data		
Bulk RNA-seq data	This study. Illumina HiSeq4000.	GEO: GSE203375
Day 90 hPSC-CMs generated in monolayers using the standard WNT-ON/WNT-OFF protocol (CHIR 99021/IWP2), Cyganek-V	Cyganek et al., 2018[6]	N/A
Day 90 hPSC-CMs generated in monolayers using the standard WNT-ON/WNT-OFF protocol (CHIR99021/IWP2) +RA, Cyganek-A	Cyganek et al., 2018[6]	N/A
Day 20 hPSC-CMs generated in monolayers in the presence of BMP4, Activin A and CHIR99021, followed by XAV939	Kuppusamy et al., 2015[32]	N/A
Day 20 hPSC-CMs generated in monolayers in the presence of BMP4, Activin A and CHIR99021 followed by XAV939	Giacomelli et al., 2020[33]	N/A
Day 20 hPSC-CMs generated in monolayers using the standard WNT-ON/WNT-OFF protocol (CHIR99021/IWP4)	Branco et al., 2019[34]	N/A
Day 6 and D8 hPSC-CMs generated using the standard WNT-ON/WNT-OFF (CHIR99021/IWR1) protocol.	Zhang et al., 2019[48]	N/A
Human adult ventricular tissues.	Yang et al., 2014[88]	N/A
Human fetal ventricular tissues	Kuppusamy et al., 2015[32]	N/A
Experimental models: Cell lines		
WA09 hESC	WiCell	WA09
WA01 hESC	WiCell	WA01
MYL2-GFP hiPSC	Allen Institute	AICS-0060-027
Oligonucleotides		
Primers used in this study	This paper	See primer table (STAR Methods)
Software and algorithms		
Fiji-ImageJ	Shindelin et al., 2012[89]	https://imagej.net/Fiji/Downloads
GraphPad Prism 8.2.0	GraphPad	N/A
MUSCLEMOTION	Sala et al., 2018[90]	N/A
Clampex 10.0	Molecular Devices Axon Instruments	N/A
nf-core/rnaseq v3.0	Ewels et al., 2020[91]	https://nf-co.re/rnaseq
STAR version	Dobin et al., 2013[92]	https://github.com/alexdobin/STAR
RSEM	Li and Dewey, 2011[93]	https://github.com/deweylab/RSEM
R v4.0.2	R Core Team, 2021	https://www.r-project.org/
DESeq2	Love et al., 2014[94]	https://bioconductor.org/packages/release/bioc/html/DESeq2.html
tximport	Soneson et al., 2015[95]	https://bioconductor.org/packages/release/bioc/html/tximport.html
PCAtools	Blighe and Lun, 2020[96]	https://bioconductor.org/packages/release/bioc/html/PCAtools.html
PoiClaClu	Witten, 2019[97]	https://cran.rstudio.com/web/packages/PoiClaClu/index.html
IHW	Ignatiadis et al., 2016[98]	https://bioconductor.org/packages/release/bioc/html/IHW.html
DE-Greport	Pantano, 2020[99]	https://www.bioconductor.org/packages/release/bioc/html/DEGreport.html
clusterProfiler	Yu et al., 2012[100]	https://bioconductor.org/packages/release/bioc/html/clusterProfiler.html

(*Continued on next page*)

Continued		
REAGENT or RESOURCE	SOURCE	IDENTIFIER
Other		
6 well plates	Corning	3506
12 well plates	Corning	3512
24 well plates	Nunc	122475
8 well Ibidi slides	Ibidi	80806
Nuncleon 96 well plates	Sigma Aldrich	P8366-50EA
35 mm MatTek dishes (14 mm)	MatTek Life Sciences	P35G-1.5-14-C
35 mm MatTek dishes (7 mm)	MatTek Life Sciences	P35G-1.5-7-C
Flow cytometry tubes	Thermo Fisher Scientific	10585801
40 μM cell strainer	VWR	734–0002
Test tube +35 μM strainer cap	Thermo Fisher Scientific	10585801

RESOURCE AVAILABILITY

Lead contact

Further information and requests for resources and reagents should be directed to and will be fulfilled by the lead contact, Andreia Sofia Bernardo (andreia.bernardo@crick.ac.uk, a.bernardo@imperial.ac.uk).

Materials availability

This study did not generate new unique reagents.

Data and code availability

- Bulk RNA-seq data have been deposited at GEO and are publicly available as of the date of publication. Accession numbers are listed in the key resources table. This paper analyses existing, publicly available data. The accession numbers for these datasets are listed in the key resources table.
- This paper does not report original code. All code is available from the lead contact upon request.
- Any additional information required to reanalyse the data reported in this paper is available from the lead contact upon request.

EXPERIMENTAL MODEL AND SUBJECT DETAILS

Human pluripotent stem cell lines' origin, characterization and maintenance

Human embryonic stem cell lines H9 (WA09, karyotype: 46, XX) and H1 (WA01, karyotype: 46, XY) were purchased from WiCell Research Institute. The induced pluripotent stem cell line MYL2-GFP (AICS0060-027, karyotype: 46, XY) was purchased from the Allen Institute. The hPSC lines were maintained in feeder-free culture conditions on Corning growth factor reduced Matrigel membrane matrix (GFR Matrigel, Corning) or geltrex (Thermo Fisher) in mTeSR1 maintenance medium (STEMCELL Technologies). Cells were passaged every 4–5 days as aggregates using Gibco Versene solution (Thermo Fisher Scientific) and cell scrapers, at a split ratio of 1:8-1:10. Prior to cryopreservation, hPSCs were assessed for genetic stability by KaryoStat and indicators of pluripotency were assessed by PluriTest (Thermo Fisher Scientific). Human stem cells were subject to routine pluripotency testing using BD Stemflow Human and Mouse Pluripotent Stem Cell Analysis Kit (BD Biosciences) as recommended by the manufacturers, or by immunostaining against OCT3/4, SOX2 and NANOG using the standard immunostaining protocol as detailed below. In house routine low-pass sequencing was also performed to confirm karyotype stability. All experiments with hESCs were approved by the UK Stem Cell Bank steering committee.

Donor heart procurement

All methods were carried out in accordance with relevant guidelines and regulations. All human hearts used for this study were non-transplantable and ethically obtained by informed legal consent (first person or next-of-kin) from cadaveric organ donors in the United States (US). Our recovery protocols and *in vitro* experimentation were pre-approved by IRBs (Institutional Review Boards) at transplant centers within the US OPTN (Organ Procurement Transplant Network). Furthermore, all transfers of the donor hearts are fully traceable and periodically reviewed by US Federal authorities. Donor characteristics, heart number, and donor identifier are shown in the key resource table and exclusion criteria were previously described.[101]

METHOD DETAILS

Directed differentiation of human pluripotent stem cell lines toward cardiomyocytes

All cell lines were dissociated using TrypLE Express (Thermo Fisher Scientific) and seed as single cells in mTeSR1 (STEMCELL Technologies) supplemented with 10 μM Y-27632 (Tocris) and grown until they reached 70–80% confluency. Cell seeding density was determined using the NC200 cell counter (Chemometec, Denmark). Seeding density was optimized for each cell line; accuracy in seeding density is essential for reproducibility. Standard cardiomyocyte differentiation was performed as described previously.[4,5] In brief, on the first day of differentiation cells were moved to 'heart 1 medium' consisting of RPMI 1640 (Life Technologies) supplemented with B27-insulin (Thermo Fisher Scientific) and 6 μM CHIR99021 (Sellek Chemicals). On day 2, medium was changed to 'heart 1 media' supplemented with 1 μM IWR-1 (Sigma). On day 4 and day 6 cells were fed with 'heart 1 media' alone. From day 8, cells were grown in media containing insulin (i.e. where insulin is presence in the B27 supplement – Thermo Fisher Scientific), and cells were kept in this media thereafter. Metabolic selection was performed from days 10–12 in RPMI 1640 devoid of glucose (Life Technologies) and supplemented with 4 mM lactate (Sigma). To optimise the differentiation of cells into left ventricular cardiomyocytes, cells were cultured in 'heart media 1' supplemented with Activin A (5 ng/mL – R&D), FGF2 (5 ng/mL - R&D) and varying amounts of BMP4 (0–7 ng/mL - R&D) and CHIR99021 (0-8 μM - Sellek Chemicals) for 24h. Next, cells were cultured in media alone for 24h following by 2 days of culture in 'heart media 1' supplemented with 1 μM IWR-1 (Sigma) and 65 μg/mL L-ascorbic acid (Sigma Aldrich). At days 4 and 6 cells were fed with 'heart media 1' supplemented with 65 μg/mL L-ascorbic acid. From day 8, cells were moved to media containing insulin (i.e. where insulin is presence in the B27 supplement) and supplemented with 65 μg/mL L-ascorbic acid, and cells were kept in this media thereafter. Throughout the protocol cells were grown either in the presence or absence of the pan RA inhibitor AGN193109 (Santa Cruz) for the first 8 days of culture and/or the presence or absence of vitamin A from day 8 and onwards (i.e. B27 was purchased with or without vitamin A – Thermo Fisher Scientific); presence of AGN193109 and use of B27 without vitamin A was needed for the obtention of more homogeneous LV-cardiomyocyte cultures. Metabolic selection was performed from days 10–12 in RPMI 1640 devoid of glucose and supplemented with 4 mM lactate.

Cardiomyocyte replating

Cardiomyocytes were re-plated between days 14–16 as standard using the cardiomyocyte dissociation medium from STEMCELL Technologies and following manufacturer's guidelines (STEMCELL Technologies). Cells were plated onto new dishes coated with GFR Matrigel (Corning), Fibronectin (Sigma) or Geltrex (Thermo Fisher Scientific) at a seeding density of $3x10^5$ cells/cm^2, or as indicated, in cardiomyocyte support medium (STEMCELL Technologies) supplemented with 10 μM Y-27632 (Tocris). After 24 h, the media was changed to the appropriate insulin containing media. Cells were re-plated on other days for specific assays following the same protocol.

Engineered heart tissues

Differentiated cardiomyocytes (day 40) were used to generate EHTs as previously described.[102] In brief, cells were dissociated into single cells with collagenase type II solution consisting of collagenase type II (Worthington, LS004176) diluted in 200 U/ml Ca^{2+}-free HBSS (Thermo Fisher Scientific) and supplemented with 1 mM HEPES [pH 7.4] (Thermo Fisher Scientific), 10 mM Y-27632 (Tocris), and 30 mM N-benzyl-p-toluene sulfonamide (TCI); cells were incubated for 2–3 h at 37°C. Dissociated cells were washed with a washing solution consisting of Ca^{2+} containing DMEM (Sigma) and DNase (12 mg/mL; Sigma-Aldrich), and centrifuged at 100 g for 10 min. Cells were resuspended in a DMEM solution containing Ca^{2+} and 1% penicillin/streptomycin (Thermo Fisher Scientific). Cells' concentration was adjusted to $1x10^6$ cells per EHT. EHTs were generated in agarose casting molds (2% agarose in PBS) made with custom-made Teflon spacers in 24-well plates (Nunc). The EHT mastermix was made of cells mixed with: non-cardiomyocyte media (DMEM (Sigma), 10% FCS (Thermo Fisher Scientific) and 1x glutamax (Thermo Fisher Scientific)); 5 mg/mL bovine fibrinogen (Sigma) diluted in NaCl 0.9%; 0.5 mg/mg aprotinin (Sigma), and 2x DMEM (Thermo Fisher Scientific). 100 μl of mastermix and 3 μL thrombin (100 U/ml (Biopur)) per EHT were pipetted into the agarose molds where silicone posts were placed, followed by incubation at 37°C. After fibrin polymerization (2 h), the silicone posts with successfully attached EHT were transferred into a new 24 well plate with culture medium made of DMEM (Sigma), 1% penicillin/streptomycin (Thermo Fisher Scientific), 10% horse serum (Thermo Fisher Scientific), 10 mg/mL insulin (Sigma), and 33 mg/mL aprotinin (Sigma). EHTs were fed every three days. Analyses of contractile force by video-optical recording were performed as previously described by Hansen et al., 2010 and functional analyses of spontaneous beating or with electrical pacing were performed as previously described using the ImageJ macro MuscleMotion.[73,90]

Human adult heart tissue and isolated cardiomyocytes

Upon arrival at the laboratory, hearts were re-perfused with ice-cold proprietary cardioplegic solution (AnaBios). Tissues were then dissected from the left and right ventricles and atria using micro-scissors and tweezers. Additionally, adult human primary ventricular myocytes were isolated enzymatically from the left and right ventricles.[103–105] Tissues and cardiomyocytes were immediately submerged in liquid nitrogen for approximately 30 s and then removed carefully with liquid nitrogen tongs and placed on dry ice. Next, the samples were quickly transferred and stored in a −80°C freezer.

Immunohistochemistry

hPSC-CMs were dissociated using the Stem Cell Technologies cardiac dissociation kit and seeded on Matrigel (Corning, 354230) or Geltrex (Thermo Fisher Scientific) coated ibidi slides (Ibidi) at a density of 3x10^5 cells/cm^2. At days 20, 40 or 60 the cultures were washed with PBS (Thermo Fisher Scientific), fixed with 4% paraformaldehyde/PBS (Thermo Fisher Scientific) for 10 min at room temperature, permeabilized for 10 min in a PBS solution containing with 0.2% Triton X-100 (Sigma), and blocked in a 5% donkey serum/TBS solution (Sigma) or 5% normal (pre-immune) goat serum in 1% BSA/TBS (Sigma) for 30 min at room temperature. The cultures were then incubated with primary antibodies (Antibody Table) diluted in 5% donkey serum/TBS (Sigma) or 1% BSA/TBS (Thermo Fisher Scientific) and incubated overnight at 4°C in a humid chamber. After 3 × 5 min washings with PBS, cells were incubated with secondary antibodies Alexa 488, 594, or 647 (Antibody Table) mixed in TBS for 1 h at room temperature. After a final 3 × 5 min wash, the cultures were stained with DAPI (Thermo Fisher Scientific) and were after embedded in Lisbeth's mounting medium (Tris-buffered glycerol containing n-propylgallate[106]) for confocal microscopy. The images were recorded on a Leica SP5 Confocal microscope (Leica Biosystems) or a Zeiss confocal system (Zeiss) and acquired with Leica LAS X Life Science or Zeiss Zen software, respectively. For high throughput analysis we used the Nikon Eclipse Ti2 inverted microscope.

Antibody table

Antibodies (primaries)	Species	Concentration	Source	Identifier
POU5F1 (ICC)	Mouse	1:200	Santa Cruz	Cat# SC-5279; RRID:AB_628051
NANOG (ICC)	Goat	1:200	R&D	Cat# AF1997; RRID:AB_355097
SOX2 (ICC)	Rabbit	1:200	Millipore	Cat# AB5603; RRID:AB_2286686
POU5F1-PecCP5.5 (FC)<br>Isotype-PerCP5.5	Mouse	20 µL per 1x10^6 cells	BD-Biosciences	Cat# 560794; RRID:AB_1937313<br>Cat# 552834; RRID:AB_394484
NANOG-647 (FC)<br>Isotype-647 (FC)	Mouse	5 µL per 1x10^6 cells	BD-Biosciences	Cat# 561300; RRID:AB_10611718<br>Cat# 557714; RRID:AB_396823
SOX2-488 (FC)<br>Isotype-488 (FC)	Mouse	2.5 µL per 1x10^6 cells	BD-Biosciences	Cat# 561593; RRID:AB_10894382<br>Cat# 557721; RRID:AB_396830
SSEA1-PE (FC)<br>Isotype-PE (FC)	Mouse	20 µL per 1x10^6 cells	BD-Biosciences	Cat# 560886; RRID:AB_10584320<br>Cat# 555584; RRID:AB_395960
SSEA4-647 (FC)<br>Isotype-647 (FC)	Mouse	5 µL per 1x10^6 cells	BD-Biosciences	Cat# 563119; RRID:AB_2738015<br>Cat# 563274; RRID: AB_2869481
TNNT2 (ICC)	Mouse	1:200	Invitrogen	Cat# MA5-12960; RRID:AB_11000742
TNNT2-PE (FC)<br>Isotype-PE (FC)	Mouse	5 µL per 1x10^6 cells	BD	Cat# 564767; RRID:AB_2738939<br>Cat# 554680; RRID:AB_395506
MYL2 (ICC/FC)	Rabbit	1:100/1:100 for 5x10^5 cells	Proteintech	Cat# 10906-1-AP; RRID:AB_2147453
HAND1 (ICC/FC)	Goat	1:100/1:100 for 5x10^5 cells	R&D	Cat# AF3168; RRID:AB_2115853
NR2F2 (ICC)	Mouse	1:150	R&D	Cat# PP-H7147-00; RRID:AB_2155627
TNNI3 (ICC)	Goat	1:200	Abcam	Cat# Ab56357; RRID:AB_880622
NKX2.5 (ICC)	Rabbit	1:150	Santa Cruz	Cat# SC-14033; RRID:AB_650281
NKX2.5 (ICC)	Goat	1:100	Santa Cruz	Cat# SC-8697; RRID:AB_650280
GATA4 (ICC)	Rabbit	1:150	Cell signaling	Cat# 36966; RRID:AB_2799108
MSX1 (ICC)	Goat	1:200	R&D	Cat# AF5045; RRID:AB_2148804
TBXT, BRA (ICC)	Goat	1:150	R&D	Cat# AF2085; RRID:AB_2200235
EOMES (ICC)	Rabbit	1:200	Abcam	Cat# Ab23345; RRID:AB_778267
ISL1 (ICC)	Goat	1:200	R&D	Cat# AF1837; RRID:AB_2126324
ISL1 (ICC)	Rabbit	1:50	Abcam	Cat# Ab20670; RRID:AB_881306
TBX5 (ICC)	Rabbit	1:50	Thermo Fisher Scientific	Cat# 42–6500; RRID:AB_2533533
FOXA2 (ICC)	Rabbit	1:150	Cell Signaling	Cat# 8186; RRID:AB_10891055
CTNNB1 (ICC/WB)	Rabbit	1:200/1:1000	Sigma	Cat# C2206; RRID:AB_476831
ACTA2 (ICC)	Mouse	1:200	cline 1A4; Sigma	Cat# A2547; RRID:AB_476701
TCAP (ICC)	Mouse	1:20	Santa Cruz	Cat# SC-25327; RRID:AB_628340
MYOM1 (ICC)	Mouse	1:100	clone B4;[107]	N/A
MYOM2 (ICC)	Mouse	1:5	[108]	N/A

(Continued on next page)

Continued				
Antibodies (primaries)	Species	Concentration	Source	Identifier
DES (ICC)	Mouse	1:200	Dako	Cat# M0760; RRID:AB_2335684
ACTN2 (ICC)	Rabbit	1:500	Abcam	Cat# Ab68167, RRID:AB_11157538
Ki67 (ICC)	Rabbit	1:200	Abcam	Cat# Ab15580; RRID:AB_443209
RYR2 (ICC)	Mouse	1:200	Abcam	Cat# Ab2868; RRID:AB_2183051
ACTN2 (ICC)	Mouse	1:400	Sigma Aldrich	Cat# A7811; RRID:AB_476766
GFP (ICC)	Rabbit	1:1000	Abcam	Cat# Ab6556; RRID:AB_305564
IK1(WB)	Mouse	1:100	Santa Cruz	Cat# SC-365265; RRID:AB_10841432
PLN (WB)	Rabbit	1:250	Abcam	Cat# Ab92697, RRID:AB_10585584
ATPA2 (WB)	Mouse	1:1000	Santa Cruz	Cat# SC-376235; RRID:AB_10989947
CTNNB1 (WB)	Rabbit	1:500	Cell Signaling	Cat# 9562S; RRID:AB_331149
TUBA1A/B (WB)	Mouse	1:2000	Sigma	Cat# T5168; RRID:AB_477579
Oxphos kit (WB)	Mouse	1:2000	Abcam	Cat# ab110411; RRID:AB_2756818
Antibodies (secondaries)	Species	Concentration	Source	Identifier
Anti-Rabbit-Alexa 488	Donkey	1:500	Thermo Fisher Scientific	Cat# A-21206; RRID:AB_2535792
Anti-Goat-Alexa 647	Donkey	1:500	Thermo Fisher Scientific	Cat# A-21447; RRID:AB_141844
Anti-Mouse-Alexa 594	Donkey	1:500	Thermo Fisher Scientific	Cat# A-21203; RRID:AB_141633
Anti-Rabbit-Alexa 594	Donkey	1:500	Thermo Fisher Scientific	Cat# A-21207; RRID:AB_141637
Anti-Goat-Alexa 594	Donkey	1:500	Thermo Fisher Scientific	Cat# A-11058; RRID:AB_142540
Anti-Mouse-Alexa 488	Donkey	1:500	Thermo Fisher Scientific	Cat# A-21202; RRID:AB_141607
Anti-Mouse-Alexa 647	Donkey	1:500	Thermo Fisher Scientific	Cat# A-31571; RRID:AB_162542
Anti-Goat-Alexa 488	Donkey	1:500	Thermo Fisher Scientific	Cat# A-11055; RRID:AB_2534102
Anti-Mouse-HRP	Goat	1:2000	Bio-Rad	Cat# 1706516; RRID:AB_2921252
Anti-Rabbit-HRP	Goat	1:2000	Bio-Rad	Cat# 1706515; RRID:AB_2617112
Anti-Mouse-IRDye 800CW	Goat	1:10000	LI-COR	Cat# 926–32210; RRID:AB_621842
Anti-Rabbit-IRDye 689LT	Goat	1:10000	LI-COR	Cat# 926–68021; RRID:AB_10706309

Image analysis

ImageJ software was used for a variety of image analyses. Images are presented as a maximal projection unless indicated otherwise. Quantification of cardiomyocytes was performed by determining the number ACTN2$^+$ cells in the field of view as a proportion of the total number of cells (DAPI). The number of LV cardiomyocytes were determined by counting the MYL2$^+$/HAND1$^+$ double-positive cells within ACTN2$^+$ cells. Quantification of ACTN2 and TNNI3 intensities relied on pixel intensity quantification and was normalised to DAPI expression. MYL2 intensity was measured as gray mean intensity values within selected MYL2$^+$ areas. Sarcomere length quantification was based on ACTN2 expression and was consistently measured from the middle of the sarcomere. Ki67 expressing cells were determined as a percentage of the total amount of cardiomyocytes as per ACTN2 and DAPI staining. Mitochondrial area, perimeter and circularity were determined following hand drawing a line around the mitochondria, i.e. a line drawn around the outer-most border of the mitochondria.

Aldefluor assay

Day 4 culture cells were dissociated using TrypLE Express (ThermoFisher Scientific), centrifuged at 100 g for 5 min and resuspended in PBS at a concentration of 1x10^6 cells in the aldefluor assay buffer. Aldehyde dehydrogenase activity was measured as per the manufacturer's guidelines using the ALDEFLUOR Kit (STEMCELL Technologies).

Flow cytometry

Cardiomyocytes were detached from cell culture plates using a Cardiomyocyte Dissociation Kit (Stem Cell Technologies) following the manufacturer's instructions. Detached cells were washed twice in Phosphate Buffered Saline (PBS) and pelleted, before fixing in 4% Paraformaldehyde (PFA) for 15 min at room temperature. After fixation, the cells were washed three times with PBS and a final wash with 1x Perm/Wash Buffer (BD Biosciences). The samples were then blocked in Perm/Wash Buffer containing 5% donkey serum for 15 min at room temperature, followed by a further wash with Perm/Wash buffer. Next cells were passed through a 35 μM cell strainer into flow cytometry tubes (Thermo Fisher Scientific) to ensure there were no clumps of cells. Unconjugated primary antibodies (see Antibody Table) diluted in BD Perm buffer and 5% donkey serum were incubated with the cells for 1 h at room temperature followed by three PBS washing steps. After, cells were incubated with Donkey AlexaFluor conjugated secondary antibodies

diluted at 1:200 in BD Perm buffer and 5% donkey serum for 1 h at room temperature. They were then washed two times in PBS. If conjugated antibodies (see Antibody Table) were used these were diluted in BD Perm buffer and 5% donkey serum and incubated with the cells for 30 min at room temperature followed by three PBS washing steps. Post staining cells were resuspended in 400 μL PBS for analysis. Fluorescence was measured on an LSR II cytometer (BD Biosciences) and analyzed using FlowJo software (FlowJo LLC).

Western blot

The differentiated cardiomyocytes (day 20, day 40 or day 60) were washed with PBS and lysed with Cell Lytic M Buffer (Sigma, C2978). Samples were diluted 4 times in SDS LB buffer (BIO-RAD) and boiled at 95°C for 10 min 15 μL of samples were loaded in each lane on a Tri-Glycine 4–20% gel (GenScript) and ran for ~40–75 min at 100–150 V. Gels were either dry transferred onto a membrane using the IBlot system (iBlot2, IB21001) for 4 min at 20 V or wet transferred for 40–60 min at 100 V in transfer buffer containing methanol. Membranes were blocked in Odyssey blocking solution (LI-COR) for 1 h at room temperature. After blocking, the membranes were incubated with primary antibodies (see Antibody Table) overnight while shaking at 4°C. After washing in TBS-T (0.1% tween, Sigma), membranes were incubated with secondary antibodies (see Antibody Table). Blots were visualized using the LI-COR system (LI-COR). All bands of interest were normalised against a loading control (see Antibody Table).

RNA extraction, cDNA synthesis and RT-qPCR

RNA was extracted using RNeasy mini kit (Qiagen, 74104) following the manufacturer's instructions and stored at −80°C until all timepoints were collected. cDNA was prepared using the Maxima First Strand cDNA Synthesis kit (Thermo Fisher Scientific, K1641), following the manufacturer's instructions, and including a dsDNase treatment. The cDNA was diluted (1:20) in RNase free water before running the RT-qPCR analysis on a Roche Lightcycler 480 II (Roche Holding AG) using LightCycler 480 SYBR Green I Master mix (Roche Holding AG, 04887352001). The primer sequences used in the qPCR analysis are detailed in Primer Table and had an efficiency of 2 ± 10%. Relative gene expression was calculated using the ΔΔCt method, normalising each gene to the housekeeping gene PBGD.

Primer table

Gene	Forward	Reverse
HAND1	CAGCTACATCGCCTACCTGA	AATCCTCTTCTCGACTGGGC
NR2F2	GCCTGTGGTCTCTCTGATGT	GAATCTCGTCGGCTGGTTG
MYL2	AGGCTGATTACGTTCGGGAA	TCTCTTCTCCGTGGGTGATG
HOXB4	GCAAAGAGCCCGTCGTCTAC	GTCAGGTAGCGGTTGTAGTGAAAT
NANOG	CATGAGTGTGGATCCAGCTTG	CCTGAATAAGCAGATCCATGG
SOX2	TGGACAGTTACGCGCACAT	CGAGTAGGACATGCTGTAGGT
POU5F1	AGTGAGAGGCAACCTGGAGA	ACACTCGGACCACATCCTTC
IRX4	GGGCTATGGCAACTACGTGA	CGAACCATCCTTGGAATCAA
HEY2	ATTTCATCCCCGATCCCTCC	ATTTCATCCCCGATCCCTCC
TBX5	GCTGGAAGGCGGATGTTT	GCTGGAAGGCGGATGTTT
HCN4	GGCCAGCACGTCACTCTG	ATGGCAGTTTGGAGCGCA
NKX2.5	CGGCCAAGTGTGCGTCTG	TTTCGGCTCTAGGGTCCTTG
TNNT2	TCCAGAAGACAGAGCGGAAA	CTTCATTCAGGTGGTCAATGG
MYH7	AAGCCATCCTGAGTGCCTTC	AACTTGTCTGCCTGGGTCAG
RYR2	ACACCAGATATGAAATGTGACGA	ATTTCATCCCCGATCCCTCC
ATP2A2	CAGCCTTTGTAGAACCTTTTGT	AATCCGCTGCACACTCTTTC
CACNA1C	GGACTCCTCTTTCACCCCAA	AGCGCCTTCACATCAAATCC
TNNI1	TGGTGGATGAGGAGCGATAC	GTCCATCACCTTCAGCTTCAG
TNNI3	CGTGTGGACAAGGTGGATGA	CCGCTTAAACTTGCCTCGAA
KCNJ2	TGTGTTTGATGTGGCGAGTG	CTGGATTTGAGGAGCTGTGC

Transmission electron microscopy

hPSC-CMs grown on 35 mm MatTek glass bottom dishes were fixed with 2.5% glutaraldehyde and 4% formaldehyde in 0.1M phosphate buffer (PB, EMS) for 30 min at room temperature. After fixation, cells were kept in 1% formaldehyde (Sigma) in 0.1 M PB at 4°C

prior to further processing. Samples were incubated with 2% reduced osmium (Osmium tetroxide, TAAB, plus potassium ferricyanide, Sigma) for 1 h at 4°C, washed 3 × 5 min in ddH_20, incubated with 1% thiocarbohydrazide (Sigma) for 20 min at RT, washed 3 × 5 min in ddH_20, incubated in 2% aqueous osmium tetroxide (TAAB) for 30 min at RT, washed 3 × 5 min in ddH_20, and incubated in 1% uranyl acetate (AGAR Scientific) at 4°C overnight. Samples were washed 3 × 5 min in ddH_20, incubated in lead aspartate for 30 min at 60°C and washed 3 × 5 min in ddH_20. The glass coverslip was removed from the dish using a razor blade and placed in a Petri dish. Samples were dehydrated with a graded ethanol series (70%, 90%, 100%). Coverslips were transferred to foil dishes and infiltrated with 50:50 propylene oxide: Durcupan (Sigma) for 1h at RT, then with 100% Durcupan twice for 2 h at RT. The samples were transferred to fresh 100% Durcupan and polymerised at 60°C for 48 h. 70 nm sections were cut with a diamond knife on a Leica UC6 ultramicrotome, and sections imaged using a Tecnai G2 Spirit Biotwin transmission electron microscope (ThermoFisher Scientific) with an Orius CCD camera (Gatan).

MicroCT/X-ray microscope imaging

EHTs were removed from posts and mounted with pins into wax prior to being fixed in 2.5% glutaraldehyde (Sigma) and 4% formaldehyde (Sigma) in 0.1M phosphate buffer (PB, (EMS)) at room temperature. Next, these were washed and post fixed in 2% reduced osmium (Osmium tetroxide, TAAB, plus potassium ferricyanide, Sigma) for 1h at 4°C, washed 3 × 5 min in ddH_20, incubated with 1% thiocarbohydrazide (Sigma) for 20 min at RT, washed 3 × 5 min in ddH_20, incubated in 2% aqueous osmium tetroxide (TAAB) for 30 min at RT, washed 3 × 5 min in ddH_20, and incubated in 1% uranyl acetate (AGAR Scientific) at 4°C overnight. Samples were washed 3 × 5 min in ddH_20, incubated in lead aspartate for 30 min at 60°C and washed 3 × 5 min in ddH_20. Samples were dehydrated with a graded ethanol series (30%, 50%, 70%, 90%, 100%) on a rotator. Next, samples were infiltrated with 50:50 propylene oxide: Durcupan (Sigma) overnight on a rotator, then with fresh 100% Durcupan on a rotator for 5h at RT. The samples were transferred to fresh 100% Durcupan and polymerised at 60°C for 48h. Excess resin was trimmed away from the EHTs with a razor blade. Scans were performed to produce microCT data at 40kV/3W, 1601 projections with LE2 filter for Std-EHTs and LE3 filter for LV-EHTs, and a pixel size of 0.954 μm for Std-EHTs and 0.959 μm for LV-EHTs using an Xradia 510 Versa X-ray microscope (Zeiss). The data was automatically reconstructed using Scout-and-Scan Control System Reconstructor software (Zeiss) and viewed in Dragonfly software (Object Research Systems).

Mitochondrial respiration

hPSC-CMs were seeded overnight onto Geltrex (Thermo Fisher Scientific) coated microplates (part of the Seahorse XF flux pack) at a density of 30,000 per well of a 96 well plate. One hour prior to measurement, medium was replaced with Seahorse XF DMEM medium supplemented with Seahorse XF Glucose (10 mM), Pyruvate (1 mM) and L-Glutamine (2 mM) (Agilent) and cells were incubated at 37°C in an incubator without CO_2 for 1 h. The Cell Mito Stress kit (Agilent) was used to assess mitochondrial function. In brief, oxygen consumption rate was measured at baseline, upon oligomycin (2.5 μM), FCCP (2. μM) and rotenone/antimycin (1 μM) injections (Agilent, 103015-100) using the Seahorse XFe96 Analyzer. Data were normalized to the number of cells, as determined by counting DAPI (Thermo Fisher Scientific) stained nucleus. Maximal respiration was calculated as the difference between FCCP and Rot/AA measurements and spare respiratory capacity was calculated as the difference between maximal and basal respiration.

Optical mapping

hPSC-CMs were seeded on Nuncleon 96 well plates (Sigma) at a density of $3x10^5$ cell/cm^2. On the day of analysis cells were loaded either with the voltage dye di-4-ANNEPS (4 μM, Thermo Fisher Scientific) for 1 min or the calcium dye Fura-4F (4 μM, Thermo Fischer Scentific) for 30 min. Dyes were loaded in phenol-free/serum-free media consisting of: DMEM (Thermo Fisher Scientific), 0.2 mM D-Galactose (Thermo Fisher Scientific), 1 mM Sodium Pyruvate (Thermo Fisher Scientific), 1x Glutamax (Thermo Fisher Scientific). After loading, cells were placed back in the incubator for 20–30 min prior to being used for analysis. Images were acquired from a minimum of 4 cell locations in each experiment and CellOPTIQ[109] was used during acquisition. The sampling rate used was 10,000 Hz and recordings of 20 s with a 2 s pause in between acquisitions were acquired. For calcium drug tests, baseline measurements were acquired first and, after drug exposure, the same locations were re-analysed. Thapsigargin (Sigma) or Ryanodine (Sigma) were added at the following concentrations: 0.1, 1, 5, 10 and 20 μM and samples were analyzed after 5, 15 or 30 min of exposure. All recordings were analyzed with cellOPTIQ[109] and data was curated using Excel. Transient rate was calculated based on the number of full cycles of AP or Calcium recorded and adjusted to the time it took from the beginning of the first full transient to the end of the last full transient.

Intracellular (sharp electrode) electrophysiology

hPSC-CMs were seeded on MatTek glass bottom dishes (MatTek Life Sciences) at a density of 10,000 cells per 14 mm glass inlet 3–7 days prior to analysis. Electrophysiological recordings were acquired using micro-electrode pipettes (30–40 MΩ) connected via an Axon HS-2A x0.1LU headstage to an Axoclamp2B switch-clamp (Molecular Devices, San Jose, CA, USA). The signals were processed through a Digidata 1440A and ClampEx 10.7 software (Molecular Devices). The pipettes were filled with high K^+ internal solution containing (in mM): KCl 2000, EGTA 0.1, HEPES 5, pH 7.2). Cells were visualised using an inverted Nikon microscope (40x) and maintained at 37°C in extracellular modified Tyrode's solution containing (in mM): NaCl 140, KCl 4.5, glucose 10, HEPES 10, $MgCl_2$ 1, $CaCl_2$ 1.8, pH 7.4). Action potentials were generated using 0.5–1 nA depolarizing current pulses applied for up to 3 ms

at 0.5 Hz. Once a stable baseline was achieved, 25 action potential sweeps were recorded. Clampfit 10.7 (Molecular Devices) was used for analyses and the action potential parameters averaged (4 out of the 25 sweeps) for each hPSC-LV-CM.

QUANTIFICATION AND STATISTICAL ANALYSIS

Non-RNA-seq data

All data presented is representative of an $n \geq 3$ experiments. Sample sizes (n) represent biological replicates, i.e. independent cell culture replicates from cells grown from different passages or individual tissue samples. All graphs are represented as mean ± standard error of mean (SEM). Where more than 3 points are shown in graphs, the number of recordings indicated represent the total number cells analyzed from ≥ three independent experiments. No statistical method was used to predetermine the samples size. Randomization was performed throughout the study for imaging and was specifically employed also for mitochondrial size assessment, sarcomere length measurement and for cellOPTIQ acquisition. Due to the nature of the study, the investigators were fully blinded for most of the experiments' data acquisition and for all analysis. Imaging of EHTs was partially blinded and imaging of monolayer cultures was not blinded, except when high throughput screening was used. Where possible the analysis was automatised. Statistical significance was determined by using Student's *t* test (unpaired, two-tailed) or One-way ANOVA in GraphPad Prism 9 software. Results were considered significant at $p < 0.05$ and p values are indicated in the figures. Statistical significance of transcript abundance was determined using a Wald test. Results are presented as adjusted p values from the DESeq2 analysis and were considered significant at $p < 0.05$.

RNA-seq read alignment and quantification

Reads were processed using the publicly available nf-core rnseq pipeline v3.0[91] with the STAR/RSEM[92,93] option against human genome assembly GRCh38 and Ensembl release 95 transcript annotations to obtain gene-level abundance estimates.

Data exploration

Gene-level RSEM estimated counts and average transcripts lengths were imported into R 4.0.2 using the tximport function from the tximport package.[95] These were used to create a DESeqDataSet object for further analysis using the Bioconductor package DESeq2.[94] Data were normalised for differing library size using DESeq2's default method, leveraging the average transcript length information. Principal Components Analysis (PCA) was used to assess the relationship between gene expression across samples. Data were first variance stabilised using DESeq2's "vst" function to deal with the sampling variability of low counts, before submission to the PCAtools package's "pca" function.[96] Data were centered, but not scaled and all available genes were used. Sample similarity was assessed using a Poisson dissimilarity matrix constructed from the un-corrected normalised counts of all samples using the "PoissonDistance" function from the PoiClaClu package.[97]

Differential expression

Differential gene expression analysis was assessed between all pairs of consecutive time-points using DESeq2's Wald test, taking heart specific effects into consideration (~heart+day). Independent Hypothesis Weighting (IHW)[98] was used to control for the false-discovery rate. Genes were called significant if they passed a combined filter of i) FDR<0.01, ii) fold change > +/−2 and iii) basemean >30 from the Wald test results. Significant genes from each comparison were combined for visualisation as a heatmap. Genes differentially expressed across time-points were identified using DESeq2's Likelihood Ratio Test (LRT), taking into consideration heart-specific effects as part of the reduced design formula. (full = ~heart+day, reduced = ~heart). The top 2000 most significantly changing genes were further clustered into groups of genes sharing similar expression patterns via the degPatterns function from the DE-Greport Bioconductor package.[99] Briefly, each gene's score across replicate time groups was reduced to a mean before calculation of a pairwise gene-expression distance matrix based on a kendall correlation statistic. Divisive hierarchical clustering (diana) of the distance matrix was used to identify gene clusters such that the variability between clusters was larger than within clusters. Clusters containing fewer than contain fewer than 50 genes were not considered.

GO enrichment analysis

Gene lists were assessed for enrichment for Gene Ontology (GO) Biological Process (BP) terms using the Bioconductor package clusterProfiler,[100] using Entrez gene mappings against a background of the human genome. p values were adjusted for multiple corrected using the Benjamini-Hochberg method and thresholded at $q < 0.05$. For the purposes of visualisation, the top 4 most enriched GO:BP categories from each gene list are presented.

Gene expression heatmaps

Heatmaps were generated using the variance stabilised data. Data were additionally scaled per gene using a *Z* score to aid visualisation. Columns (samples) and rows (genes) were each hierarchically clustered using a "complete" clustering method on a set of Euclidean distances.

Cell Reports Methods, Volume *3*

Supplemental information

Generation of left ventricle-like cardiomyocytes with improved structural, functional, and metabolic maturity from human pluripotent stem cells

Nicola Dark, Marie-Victoire Cosson, Lorenza I. Tsansizi, Thomas J. Owen, Elisa Ferraro, Alice J. Francis, Selina Tsai, Camille Bouissou, Anne Weston, Lucy Collinson, Najah Abi-Gerges, Paul E. Miller, Kenneth T. MacLeod, Elisabeth Ehler, Richard Mitter, Sian E. Harding, James C. Smith, and Andreia S. Bernardo

Supplemental Figures and Legends

Figure S1

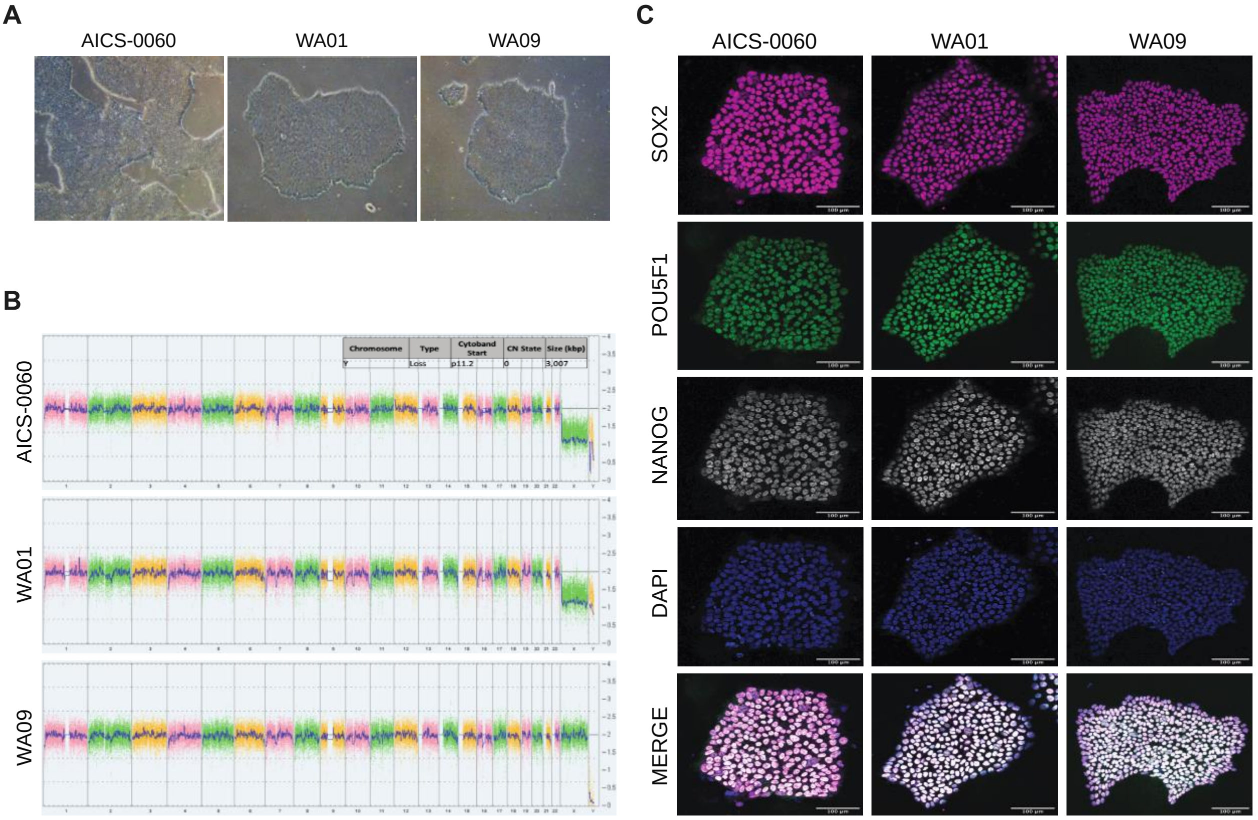

Figure S1. Stem cell lines were pluripotent and karyotypically normal. A Bright field micrographs showing examples of hPSC cultures. The cell lines are indicated. **B** KaryoStat results for the 3 hPSC lines. Note the AICS line is missing a small portion of the Y chromosome. **C** Immunostaining micrographs of pluripotent cultures (cell lines are indicated at the top) stained for the main stem cell transcription factors and the nuclear dye DAPI. Scale bar: 100 µm. (See also Figure 1)

Figure S2

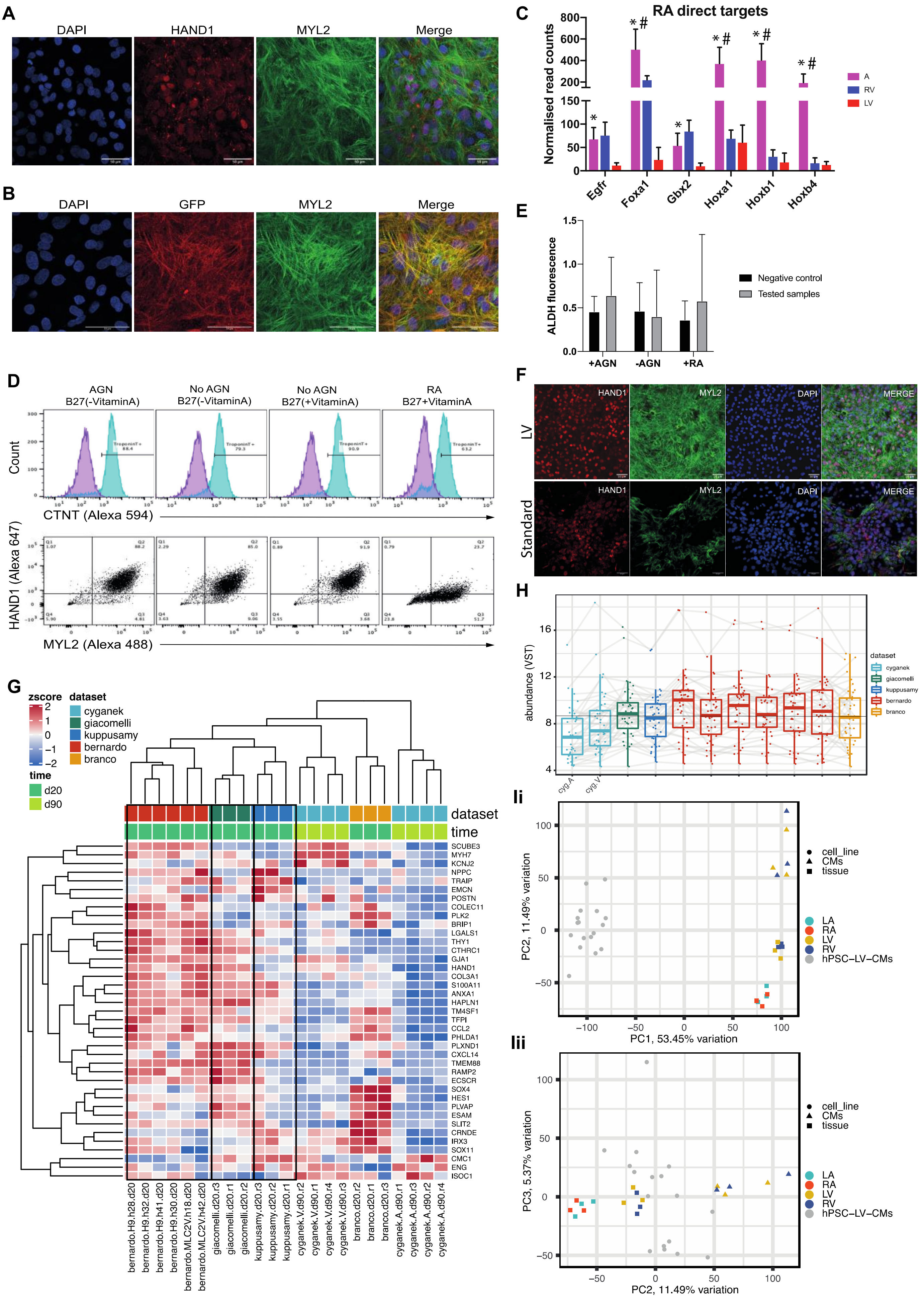
A
DAPI
HAND1
MYL2
Merge
B
DAPI
GFP
MYL2
Merge
C
RA direct targets
Normalised read counts
A
RV
LV
Egfr
Foxa1
Gbx2
Hoxa1
Hoxb1
Hoxb4
D
AGN B27(-VitaminA)
No AGN B27(-VitaminA)
No AGN B27(+VitaminA)
RA B27+VitaminA
Count
CTNT (Alexa 594)
HAND1 (Alexa 647)
MYL2 (Alexa 488)
E
ALDH fluorescence
Negative control
Tested samples
+AGN
-AGN
+RA
F
LV
Standard
HAND1
MYL2
DAPI
MERGE
G
zscore
dataset
cyganek
giacomelli
kuppusamy
bernardo
branco
time
d20
d90
H
abundance (VST)
dataset
cyganek
giacomelli
kuppusamy
bernardo
branco
Ii
PC1, 53.45% variation
PC2, 11.49% variation
cell_line
CMs
tissue
LA
RA
LV
RV
hPSC−LV−CMs
Iii
PC2, 11.49% variation
PC3, 5.37% variation
cell_line
CMs
tissue
LA
RA
LV
RV
hPSC−LV−CMs

Figure S2. Generation of hPSC-LV-CMs using a two-step approach. A Immunostaining micrographs of day 20 hPSC-LV-CMs generated from the WA01 hESC line. The LV-differentiation protocol was optimised for this line as in Fig. 1B (data not shown). Optimal conditions relied on 3 μM CHIR and 3 ng/ml BMP4. Cells were stained for MYL2, HAND1 and DAPI. Scale bar: 50 μm. **B** Immunostaining micrographs of day 20 hPSC-LV-CMs generated from the MYL2-GFP hiPSC line from the Allen Institute. The LV-differentiation protocol was optimised for this line as in Fig. 1B (data not shown). Optimal conditions relied on 3 μM CHIR and 3 ng/ml BMP4. Cells were stained for GFP, MYL2 and DAPI. Scale bar: 50 μm. **C** Graph showing the normalised read counts for a panel of retinoic acid (RA) direct target genes within mouse E8.5 atrial (A), right ventricle (RV) and left ventricle (LV) regions. **D** Flow cytometry analysis of hPSC-CMs grown in the presence or absence of retinoic acid (+/- AGN, +/-VitA). AGN (AGN193109) is a pan-retinoic acid inhibitor. Top panel shows expression of TNNT2, and bottom panel shows expression of HAND1 and MYL2. **E** Flow cytometric analyses of day 4 ALDH (Aldehyde dehydrogenase) activity within samples grown in the presence or absence of the retinoic acid inhibitor AGN. **F** Immunostaining micrographs of day 20 hPSC-CMs generated using the Standard (WNT-ON/WNT-OFF) or LV differentiation protocols. Scale bar: 50 μm. **G** Heat map showing the relative-expression levels of LV- associated genes, as described in DeLaughter et al.[1], within hPSC-CMs generated using the LV-differentiation protocol (Bernardo dataset) or alternative protocols described in the literature[2-5]. **H** Box plot distribution of the expression level of the various LV-associated genes[1]. Expression is based on transcripts per million (TPM). The black line is a reference point. **I** Principal component analysis (PCA) of hPSC-LV-CMs (days 20, 40 and 60) and human adult cardiomyocyte tissues or isolated cardiomyocytes. LA stands for left atria, RA for right atria, LV for left ventricle and RV for right ventricle. **Ii** represents PCA1 and PCA2. **Iii** Represents PCA2 and PCA3. (See also Figure 1)

Figure S3

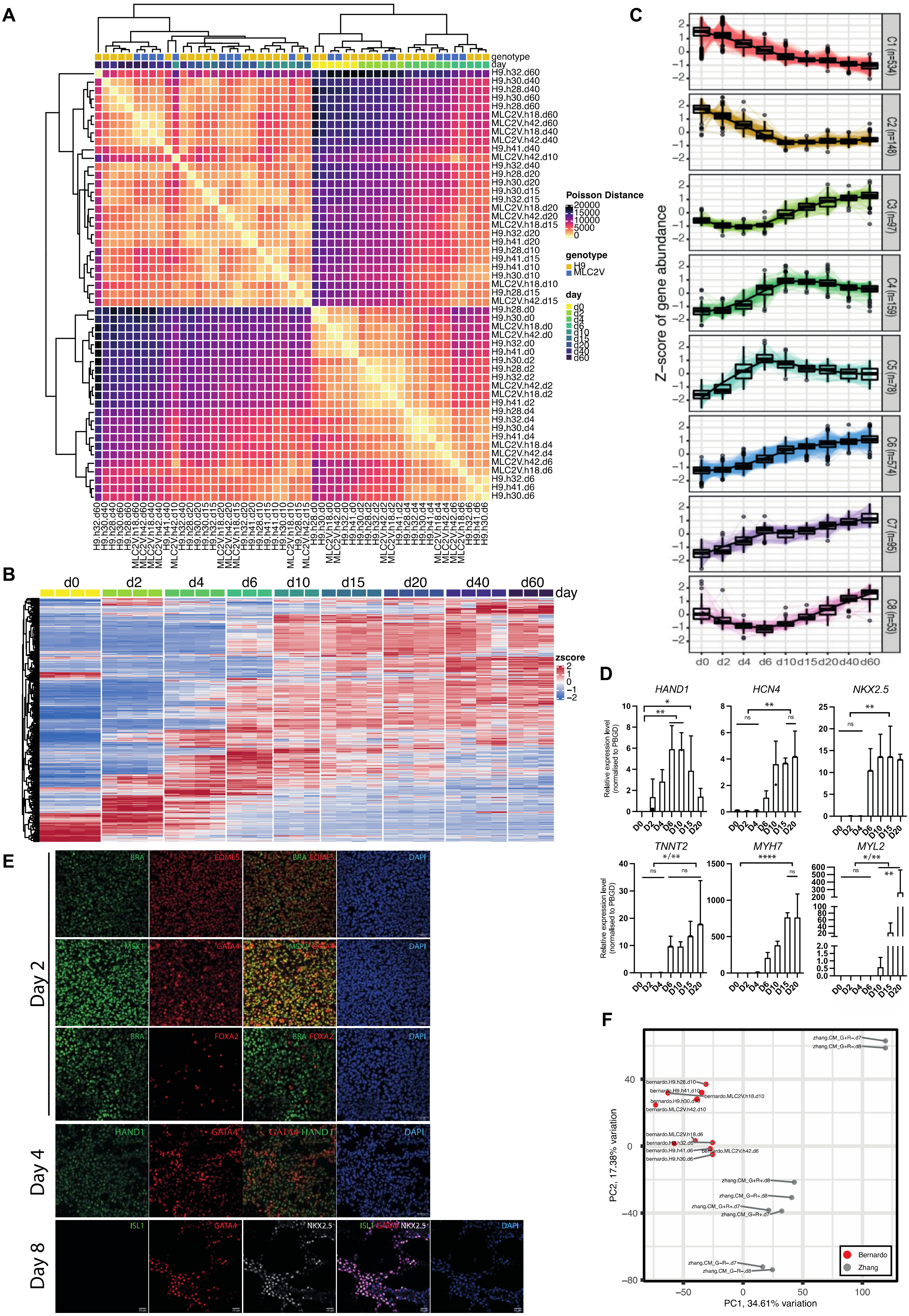
A
B
C
D
E
F
Poisson Distance
genotype
H9
MLC2V
day
zscore
Z-score of gene abundance
HAND1
HCN4
NKX2.5
TNNT2
MYH7
MYL2
Relative expression level (normalised to PBGD)
Day 2
Day 4
Day 8
PC1, 34.61% variation
PC2, 17.38% variation
Bernardo
Zhang

Figure S3. hPSC-LV-CMs arise from first heart field progenitors. A Poisson distance analysis of hPSC-LV-CMs at different time points. Two different cell lines are indicated (H9, WA09 hESC, and MLC2V, MYL2-GFP hiPCS). **B** Heatmap based on multiple pairwise comparisons using a Wald test. **C** Graphs depicting the expression changes associated with each individual cluster (C). Clustering analysis was based on hierarchical clustering, and we used a likelihood ratio test (LTR) based on the top 2000 most significant genes to perform this cluster analysis. **D** Graphs showing qRT-PCR expression data for cardiomyocyte markers. Data was normalised to PBGD. ** $p \leq 0.01$ *** $p \leq 0.001$, **** $p \leq 0.0001$. **E** Immunostaining micrographs of day 2, day 4 and day 8 cultures generated using the hPSC-LV-CM protocol. Cells were stained for the mesoderm markers (BRA, EOMES, MSX1), the mesendoderm cardiac specific marker FOXA2 and cardiomyocyte progenitor markers (GATA4, HAND1, ISL1, NKX2.5). Scale bar: 50 μm (top 4 panels) or 33 μm (bottom panel).
F Principal component analysis (PCA) of hPSC-LV-CMs (days 6 and 10) and day 7/8 hPSC-CMs generated via the Standard WNT-ON/WNT-OFF protocol and expressing TBX5 (G^+) and/or NKX2.5 (R^+) or none (G^-R^-), data extracted from Zhang et al.[6]. (See also Figure 2)

Figure S4

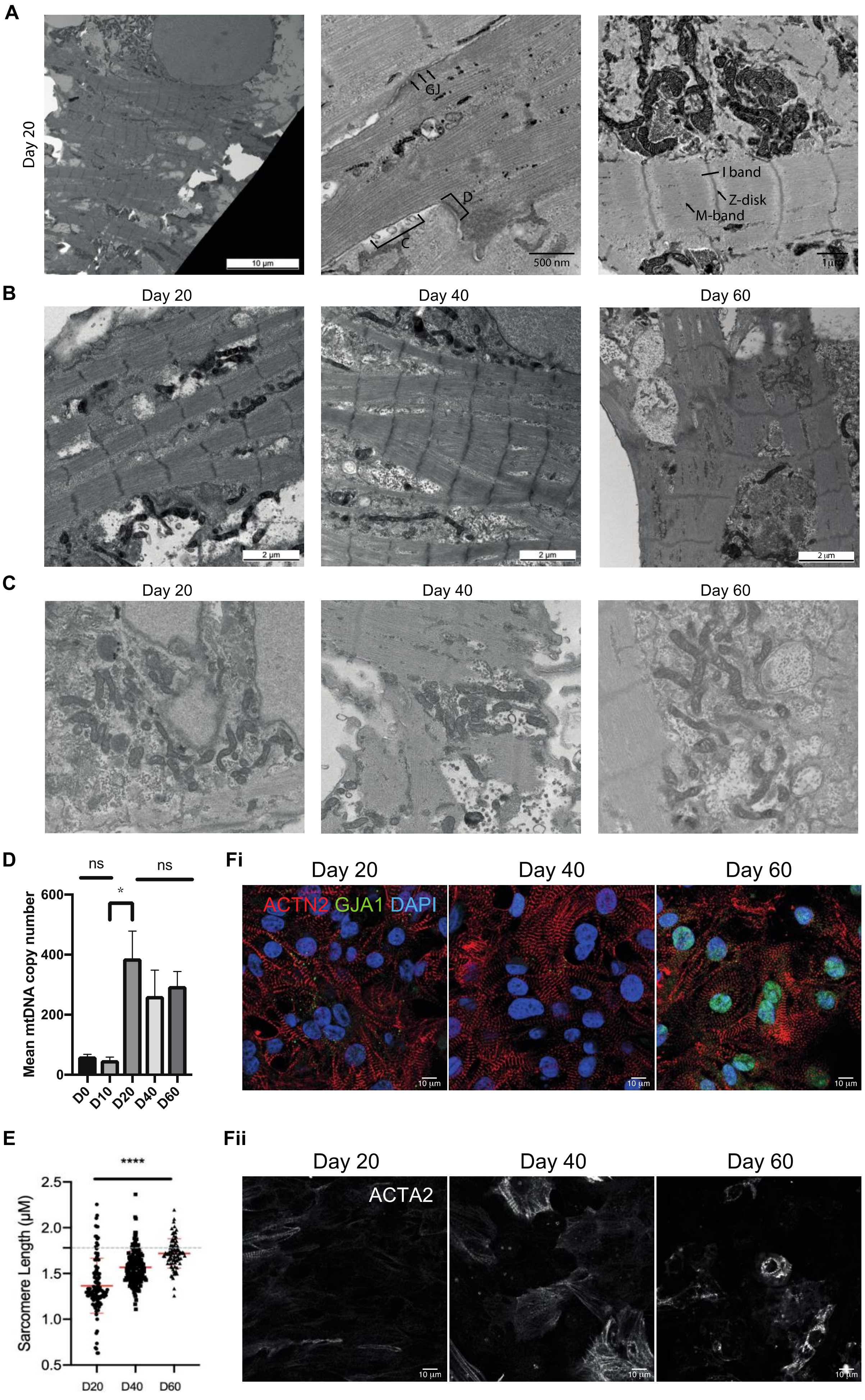
A
Day 20
GJ
D
C
10 µm
500 nm
I band
Z-disk
M-band
1 µm
B
Day 20
Day 40
Day 60
2 µm
C
Day 20
Day 40
Day 60
D
ns
ns
*
Mean mtDNA copy number
600
400
200
0
D0
D10
D20
D40
D60
Fi
Day 20
Day 40
Day 60
ACTN2 GJA1 DAPI
10 µm
E
****
Sarcomere Length (µM)
2.5
2.0
1.5
1.0
0.5
D20
D40
D60
Fii
Day 20
Day 40
Day 60
ACTA2
10 µm

Figure S4. hPSC-LV-CMs mature fast *in vitro*. A Transmission electron micrographs of day 20 hPSC-LV-CMs. On the left, a zoomed-out view of a CM exhibiting organised sarcomeres can be seen. Scale bar: 10 μm. In the middle, is a zoomed-in view showing three adjacent cells. Within these, a possible desmosome (D), can be seen. A gap junction (GP) and some possible caveolae (C) can also be seen. Scale bar: 500 nm. On the right, clear sarcomere structures can be observed including the Z-disk, I-band, and M-line. Scale bar: 1 μm. **B** Transmission electron micrographs of day 20 (left), day 40 (middle) and day 60 (right) hPSC-LV-CMs. Images focused on sarcomeres. Scale bar: 2 μm. **C** Transmission electron micrographs of day 20 (left), day 40 (middle) and day 60 (right) hPSC-LV-CMs. Images focused on mitochondria. **D** Graph showing the mean mitochondrial DNA copy number of hPSC-LV-CMs at different times points (days, D) as determined using qPCR. * $p \leq 0.05$. **E** Graph showing the sarcomere length of hPSC-LV-CMs at days (D) 20, 40 and 60, as determine using transmission electron micrographs and Fiji. **F** Immunostaining micrographs of hPSC-LV-CMs at days 20, 40 and 60 stained for: ACTA2; the Gap-junction marker GJA1; DAPI (**Fi**) and the stress marker ACTA2 (**Fii**). Scale bar: 10 μm. (See also Figure 3)

Figure S5

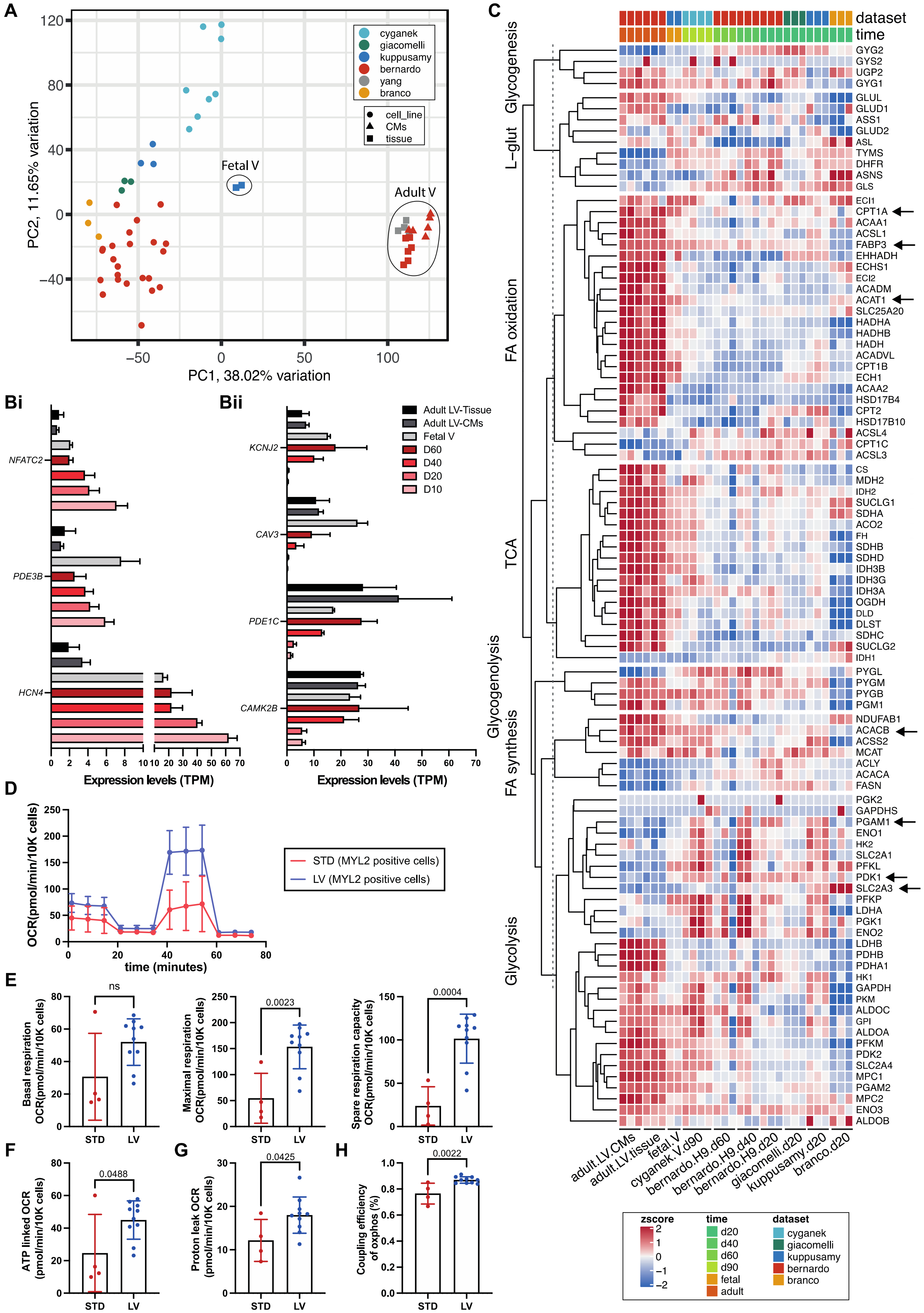
A
cyganek
giacomelli
kuppusamy
bernardo
yang
branco
cell_line
CMs
tissue
Fetal V
Adult V
PC2, 11.65% variation
PC1, 38.02% variation
Bi
Bii
NFATC2
PDE3B
HCN4
KCNJ2
CAV3
PDE1C
CAMK2B
Adult LV-Tissue
Adult LV-CMs
Fetal V
D60
D40
D20
D10
Expression levels (TPM)
C
dataset
time
Glycogenesis
L−glut
FA oxidation
TCA
Glycogenolysis
FA synthesis
Glycolysis
adult.LV.CMs
adult.LV.tissue
fetal.V
cyganek.V.d90
bernardo.H9.d60
bernardo.H9.d40
bernardo.H9.d20
giacomelli.d20
kuppusamy.d20
branco.d20
zscore
d20
d40
d60
d90
fetal
adult
D
OCR(pmol/min/10K cells)
time (minutes)
STD (MYL2 positive cells)
LV (MYL2 positive cells)
E
Basal respiration OCR(pmol/min/10K cells)
Maximal respiration OCR(pmol/min/10K cells)
Spare respiration capacity OCR(pmol/min/10K cells)
ns
0.0023
0.0004
STD
LV
F
G
H
ATP linked OCR (pmol/min/10K cells)
Proton leak OCR (pmol/min/10K cells)
Coupling efficiency of oxphos (%)
0.0488
0.0425
0.0022

Figure S5. hPSC-LV-CMs are more mature than time-matched cardiomyocytes generated using alternative protocols. A Principal component analysis (PC) of human adult tissues, human adult isolated CMs and hPSC-CMs generated by our lab (Bernardo) or by the indicated authors[2-5,7]. Ex-vivo samples were from the right or left ventricles and are indicated as Foetal V or Adult V. **B** Graphs showing the expression levels of the indicated genes in transcripts per million (TPM). **C** Heatmap showing the relative-expression levels of metabolism associated genes. The genes are organised in groups of metabolic associated activity (indicated on the left). Arrows point to genes highlighted in the main text. **D** Oxygen consumption rate (OCR) graph for day 20 ventricular hPSC-CMs isolated by flow cytometry (MYL2-GFP$^+$) from both LV and Std cultures.
E Respiration analysis of ventricular (MYL2-GFP$^+$) hPSC-CMs based on OCR. **F-H** Graphs showing the ATP production (**F**), proton leak (**G**) and coupling efficiency (**H**) of ventricular hPSC-CMs based on OCR. (See also Figure 4)

Figure S6

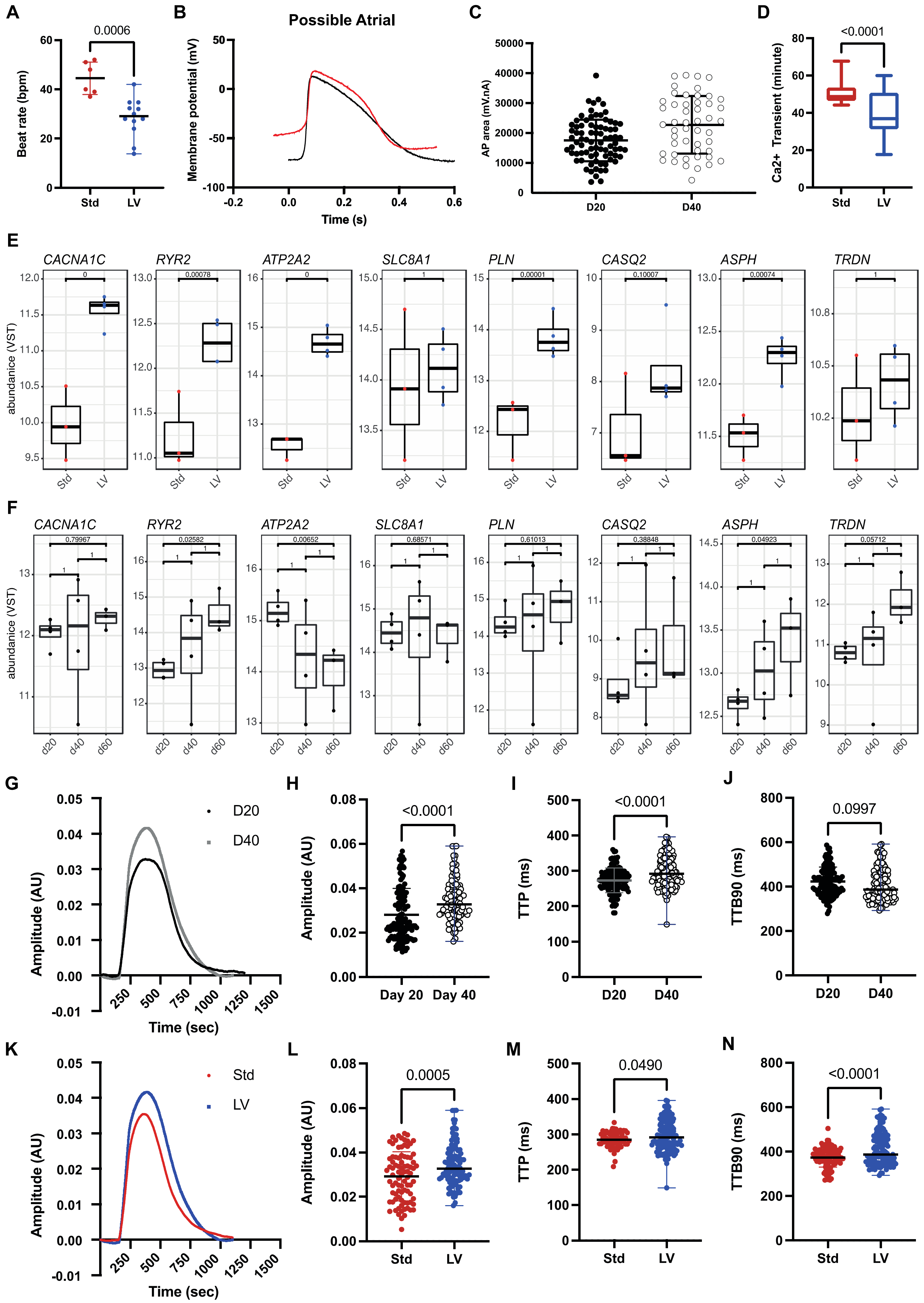
A
0.0006
Beat rate (bpm)
Std
LV
B
Possible Atrial
Membrane potential (mV)
Time (s)
C
AP area (mV.nA)
D20
D40
D
<0.0001
Ca2+ Transient (minute)
Std
LV
E
CACNA1C
RYR2
ATP2A2
SLC8A1
PLN
CASQ2
ASPH
TRDN
abundanice (VST)
F
CACNA1C
RYR2
ATP2A2
SLC8A1
PLN
CASQ2
ASPH
TRDN
abundanice (VST)
G
Amplitude (AU)
D20
D40
Time (sec)
H
<0.0001
Amplitude (AU)
Day 20
Day 40
I
<0.0001
TTP (ms)
D20
D40
J
0.0997
TTB90 (ms)
D20
D40
K
Amplitude (AU)
Std
LV
Time (sec)
L
0.0005
Amplitude (AU)
Std
LV
M
0.0490
TTP (ms)
Std
LV
N
<0.0001
TTB90 (ms)
Std
LV

Figure S6. hPSC-LV-CMs display hallmarks of electrophysiology and calcium handling maturity.

A Graph showing the beat rate of hPSC-CMs as calculated manually at day 20. **B** Graph showing the action potential (AP) morphology of a day (D) 20 and a day 40 hPSC-LV-CM cell with an atrial-like AP shape. Note these cells still exhibit a plateau but which is less marked than that of sibling cells thus making it unclear if they are atrial or more immature ventricular AP shapes. These are the types of cells counted as possible atrial within the morphological AP assessment of the data. **C** Graph showing the AP area of day 20 and day 40 hPSC-LV-CMs. **D** Graph showing the calcium transient rate of hPSC-CMs as calculated using Fluo4AM and CellOptic at day 20. **E-F** Graphs showing the expression data for calcium related genes between Standard (Std) and LV cultures (**E**) or within hPSC-LV-CMs of different ages, i.e. grown for a different number of days (d) (**F**). Indicated are the adjusted p-values from the DESeq2 analysis. **G-J** Calcium function characterization of day (D) 20/40 hPSC-LV-CMs. Data was analysed using optical mapping. **G** Example of calcium transients (CaT).
H Graph showing the CaT amplitude. **I** Graph showing the CaT time to peak (TTP). **J** Graph showing the CaT time to baseline 90 (TTB90). **K-N** Calcium function characterization of day (D) 40 hPSC-CMs differentiated using the Standard (Std) or LV differentiation protocols. **K** Example of calcium transients (CaT). **L** Graph showing the CaT amplitude. **M** Graph showing the CaT time to peak (TTP). **N** Graph showing the CaT time to baseline 90 (TTB90). (See also Figures 5 and 6)

Figure S7

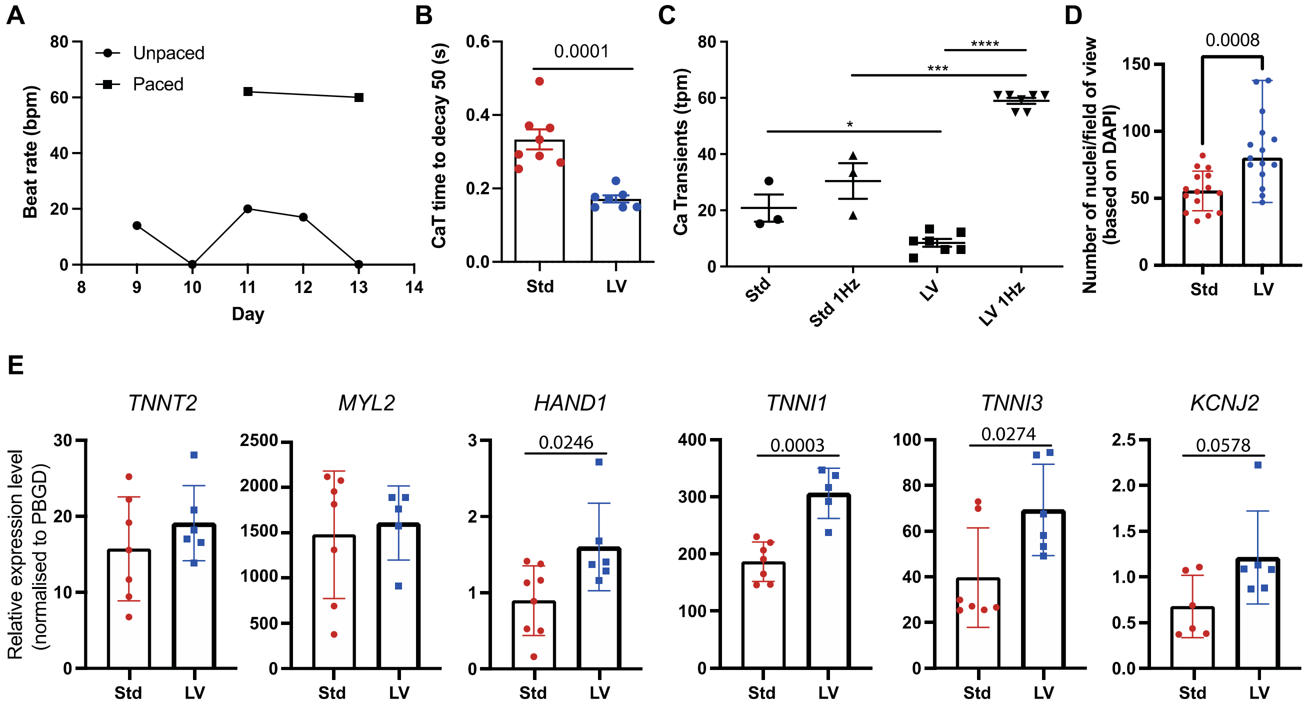

Figure S7. hPSC-LV-CMs generate more functional EHTs. Characterization of EHTs generated from day 40 hPSC-CMs differentiated using the Standard (Std) or LV differentiation protocols. **A** Graph showing an example of a LV-EHT with low to no spontaneous beating over time in culture and the respective beat rate it achieves when paced at 1 Hz. **B** Graph showing the calcium transient (CaT) analysis of EHTs as determined using confocal microscopy; CaT to decay 50 is indicated. **C** Graph showing the calcium transient (CaT), expressed as transient per minute (tpm), of EHTs beating spontaneously or paced at 1 Hz. **D** Graph showing the number of nuclei found per field of view within Std- and LV-EHTs. **E** Graphs showing the qRT-PCR expression data for various cardiac genes. Data was normalised to PBGD. (See also Figure 7)

Supplemental References

1. DeLaughter, D.M., Bick, A.G., Wakimoto, H., McKean, D., Gorham, J.M., Kathiriya, I.S., Hinson, J.T., Homsy, J., Gray, J., Pu, W., et al. (2016). Single-Cell Resolution of Temporal Gene Expression during Heart Development. Dev Cell *39*, 480-490. 10.1016/j.devcel.2016.10.001.
2. Cyganek, L., Tiburcy, M., Sekeres, K., Gerstenberg, K., Bohnenberger, H., Lenz, C., Henze, S., Stauske, M., Salinas, G., Zimmermann, W.H., et al. (2018). Deep phenotyping of human induced pluripotent stem cell-derived atrial and ventricular cardiomyocytes. JCI Insight *3*. 10.1172/jci.insight.99941.
3. Kuppusamy, K.T., Jones, D.C., Sperber, H., Madan, A., Fischer, K.A., Rodriguez, M.L., Pabon, L., Zhu, W.Z., Tulloch, N.L., Yang, X., et al. (2015). Let-7 family of microRNA is required for maturation and adult-like metabolism in stem cell-derived cardiomyocytes. Proc Natl Acad Sci U S A *112*, E2785-2794. 10.1073/pnas.1424042112.
4. Giacomelli, E., Meraviglia, V., Campostrini, G., Cochrane, A., Cao, X., van Helden, R.W.J., Krotenberg Garcia, A., Mircea, M., Kostidis, S., Davis, R.P., et al. (2020). Human-iPSC-Derived Cardiac Stromal Cells Enhance Maturation in 3D Cardiac Microtissues and Reveal Non-cardiomyocyte Contributions to Heart Disease. Cell Stem Cell *26*, 862-879 e811. 10.1016/j.stem.2020.05.004.
5. Branco, M.A., Cotovio, J.P., Rodrigues, C.A.V., Vaz, S.H., Fernandes, T.G., Moreira, L.M., Cabral, J.M.S., and Diogo, M.M. (2019). Transcriptomic analysis of 3D Cardiac Differentiation of Human Induced Pluripotent Stem Cells Reveals Faster Cardiomyocyte Maturation Compared to 2D Culture. Sci Rep *9*, 9229. 10.1038/s41598-019-45047-9.
6. Zhang, J.Z., Termglinchan, V., Shao, N.Y., Itzhaki, I., Liu, C., Ma, N., Tian, L., Wang, V.Y., Chang, A.C.Y., Guo, H., et al. (2019). A Human iPSC Double-Reporter System Enables Purification of Cardiac Lineage Subpopulations with Distinct Function and Drug Response Profiles. Cell Stem Cell *24*, 802-811 e805. 10.1016/j.stem.2019.02.015.
7. Yang, K.C., Yamada, K.A., Patel, A.Y., Topkara, V.K., George, I., Cheema, F.H., Ewald, G.A., Mann, D.L., and Nerbonne, J.M. (2014). Deep RNA sequencing reveals dynamic regulation of myocardial noncoding RNAs in failing human heart and remodeling with mechanical circulatory support. Circulation *129*, 1009-1021. 10.1161/CIRCULATIONAHA.113.003863.
