# Supplementary material for: Generation of left ventricle-like cardiomyocytes with improved structural, functional, and metabolic maturity from human pluripotent stem cells
Source: Cell Rep Methods. 2023 Apr 24;3(4):100456. doi: 10.1016/j.crmeth.2023.100456 (PMC10163040; doi:10.1016/j.crmeth.2023.100456)
Supplement: Document S1. Figures S1–S7 [file mmc1.pdf]

Cell Reports Methods, Volume *3*

Supplemental information

Generation of left ventricle-like cardiomyocytes with improved structural, functional, and metabolic maturity from human pluripotent stem cells

Nicola Dark, Marie-Victoire Cosson, Lorenza I. Tsansizi, Thomas J. Owen, Elisa Ferraro, Alice J. Francis, Selina Tsai, Camille Bouissou, Anne Weston, Lucy Collinson, Najah Abi-Gerges, Paul E. Miller, Kenneth T. MacLeod, Elisabeth Ehler, Richard Mitter, Sian E. Harding, James C. Smith, and Andreia S. Bernardo

Supplemental Figures and Legends

Figure S1

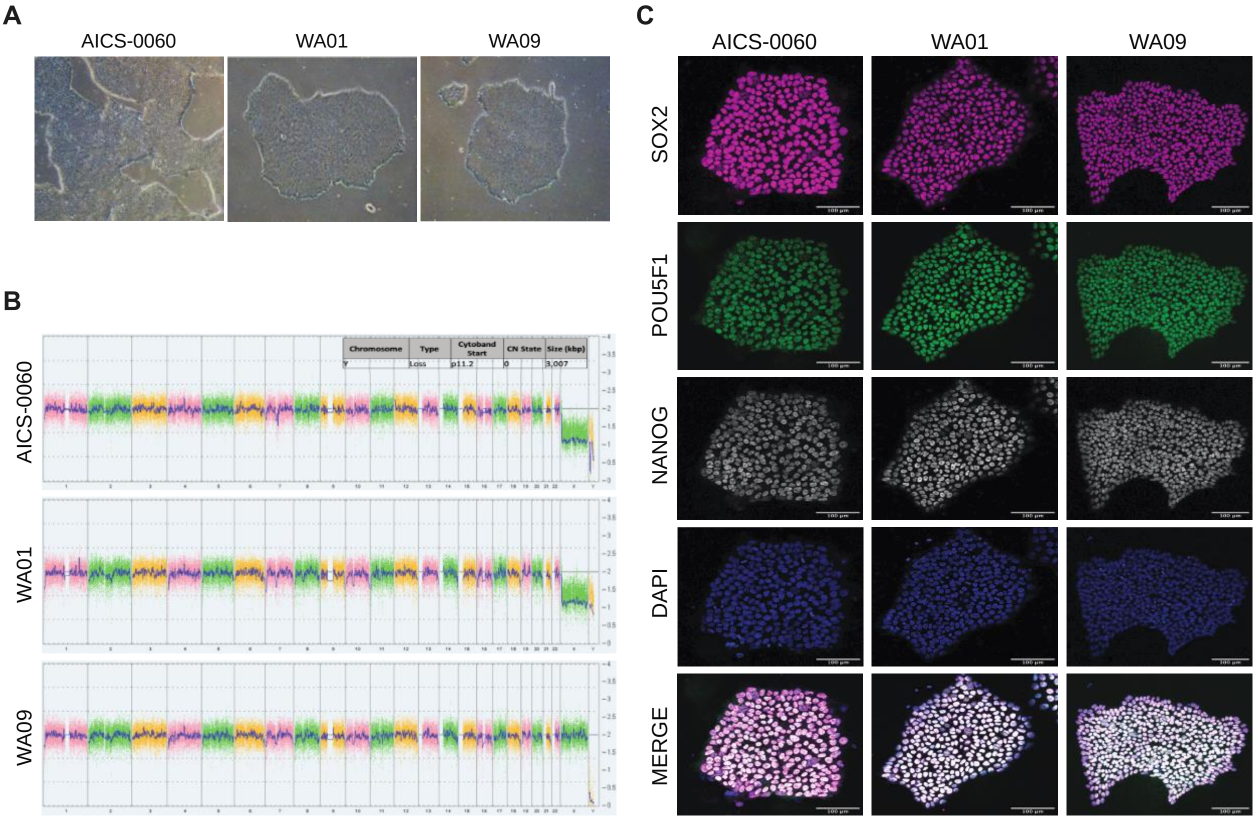

Figure S1. Stem cell lines were pluripotent and karyotypically normal. A Bright field micrographs showing examples of hPSC cultures. The cell lines are indicated. **B** KaryoStat results for the 3 hPSC lines. Note the AICS line is missing a small portion of the Y chromosome. **C** Immunostaining micrographs of pluripotent cultures (cell lines are indicated at the top) stained for the main stem cell transcription factors and the nuclear dye DAPI. Scale bar: 100 µm. (See also Figure 1)

Figure S2

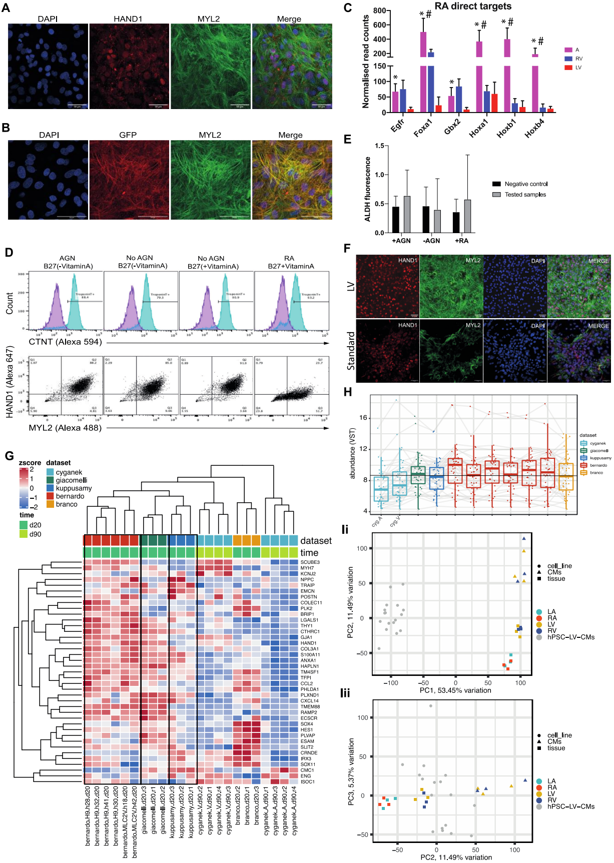

A
DAPI
HAND1
MYL2
Merge
B
DAPI
GFP
MYL2
Merge
C
RA direct targets
Normalised read counts
Egfr
Foxa1
Gbx2
Hoxa1
Hoxb1
Hoxb4
A
RV
LV
D
AGN B27(-VitaminA)
No AGN B27(-VitaminA)
No AGN B27(+VitaminA)
RA B27+VitaminA
Count
CTNT (Alexa 594)
HAND1 (Alexa 647)
MYL2 (Alexa 488)
E
ALDH fluorescence
Negative control
Tested samples
+AGN
-AGN
+RA
F
LV
Standard
HAND1
MYL2
DAPI
MERGE
G
zscore
dataset
cyganek
giacomelli
kuppusamy
bernardo
branco
time
d20
d90
H
abundance (VST)
Ii
PC2, 11.49% variation
PC1, 53.45% variation
cell_line
CMs
tissue
LA
RA
LV
RV
hPSC−LV−CMs
Iii
PC3, 5.37% variation
PC2, 11.49% variation

2

Figure S2. Generation of hPSC-LV-CMs using a two-step approach. A Immunostaining micrographs of day 20 hPSC-LV-CMs generated from the WA01 hESC line. The LV-differentiation protocol was optimised for this line as in Fig. 1B (data not shown). Optimal conditions relied on 3 μM CHIR and 3 ng/ml BMP4. Cells were stained for MYL2, HAND1 and DAPI. Scale bar: 50 μm. **B** Immunostaining micrographs of day 20 hPSC-LV-CMs generated from the MYL2-GFP hiPSC line from the Allen Institute. The LV-differentiation protocol was optimised for this line as in Fig. 1B (data not shown). Optimal conditions relied on 3 μM CHIR and 3 ng/ml BMP4. Cells were stained for GFP, MYL2 and DAPI. Scale bar: 50 μm. **C** Graph showing the normalised read counts for a panel of retinoic acid (RA) direct target genes within mouse E8.5 atrial (A), right ventricle (RV) and left ventricle (LV) regions. **D** Flow cytometry analysis of hPSC-CMs grown in the presence or absence of retinoic acid (+/- AGN, +/-VitA). AGN (AGN193109) is a pan-retinoic acid inhibitor. Top panel shows expression of TNNT2, and bottom panel shows expression of HAND1 and MYL2. **E** Flow cytometric analyses of day 4 ALDH (Aldehyde dehydrogenase) activity within samples grown in the presence or absence of the retinoic acid inhibitor AGN. **F** Immunostaining micrographs of day 20 hPSC-CMs generated using the Standard (WNT-ON/WNT-OFF) or LV differentiation protocols. Scale bar: 50 μm. **G** Heat map showing the relative-expression levels of LV- associated genes, as described in DeLaughter et al.[1], within hPSC-CMs generated using the LV-differentiation protocol (Bernardo dataset) or alternative protocols described in the literature[2-5]. **H** Box plot distribution of the expression level of the various LV-associated genes[1]. Expression is based on transcripts per million (TPM). The black line is a reference point. **I** Principal component analysis (PCA) of hPSC-LV-CMs (days 20, 40 and 60) and human adult cardiomyocyte tissues or isolated cardiomyocytes. LA stands for left atria, RA for right atria, LV for left ventricle and RV for right ventricle. **Ii** represents PCA1 and PCA2. **Iii** Represents PCA2 and PCA3. (See also Figure 1)

Figure S3

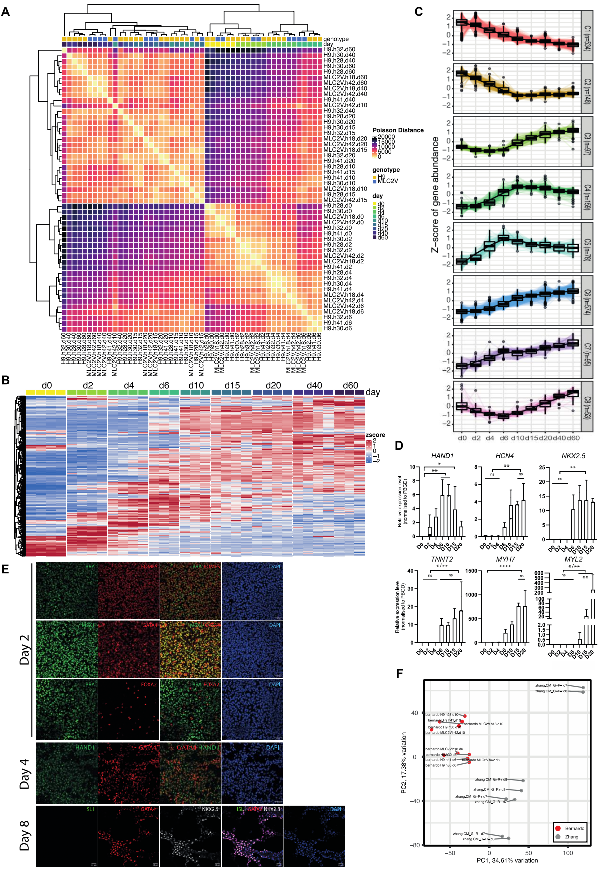

A
B
C
D
E
F
Poisson Distance
genotype
H9
MLC2V
day
zscore
Z-score of gene abundance
C1 (n=534)
C2 (n=148)
C3 (n=97)
C4 (n=159)
C5 (n=78)
C6 (n=574)
C7 (n=95)
C8 (n=53)
HAND1
HCN4
NKX2.5
TNNT2
MYH7
MYL2
Relative expression level (normalised to PBGD)
Day 2
Day 4
Day 8
BRA
EOMES
DAPI
MSX1
GATA4
FOXA2
HAND1
ISL1
NKX2.5
PC1, 34.61% variation
PC2, 17.38% variation
Bernardo
Zhang

4

Figure S3. hPSC-LV-CMs arise from first heart field progenitors. A Poisson distance analysis of hPSC-LV-CMs at different time points. Two different cell lines are indicated (H9, WA09 hESC, and MLC2V, MYL2-GFP hiPCS). **B** Heatmap based on multiple pairwise comparisons using a Wald test. **C** Graphs depicting the expression changes associated with each individual cluster (C). Clustering analysis was based on hierarchical clustering, and we used a likelihood ratio test (LTR) based on the top 2000 most significant genes to perform this cluster analysis. **D** Graphs showing qRT-PCR expression data for cardiomyocyte markers. Data was normalised to PBGD. ** $p \leq 0.01$ *** $p \leq 0.001$, **** $p \leq 0.0001$. **E** Immunostaining micrographs of day 2, day 4 and day 8 cultures generated using the hPSC-LV-CM protocol. Cells were stained for the mesoderm markers (BRA, EOMES, MSX1), the mesendoderm cardiac specific marker FOXA2 and cardiomyocyte progenitor markers (GATA4, HAND1, ISL1, NKX2.5). Scale bar: 50 μm (top 4 panels) or 33 μm (bottom panel).
F Principal component analysis (PCA) of hPSC-LV-CMs (days 6 and 10) and day 7/8 hPSC-CMs generated via the Standard WNT-ON/WNT-OFF protocol and expressing TBX5 (G^+) and/or NKX2.5 (R^+) or none (G^-R^-), data extracted from Zhang et al.[6]. (See also Figure 2)

Figure S4

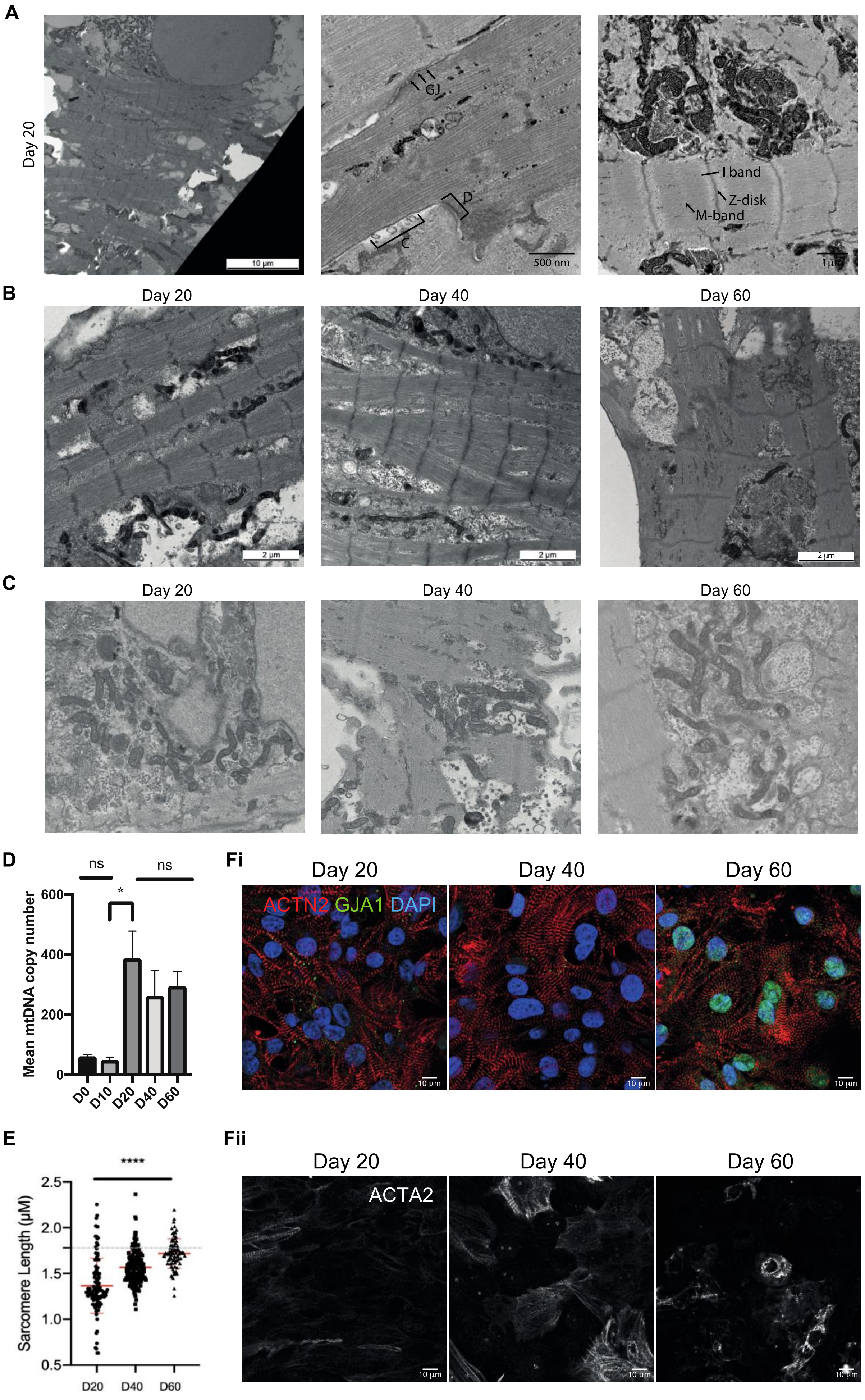

A
Day 20
10 µm
GJ
D
C
500 nm
I band
Z-disk
M-band
1 µm
B
Day 20
Day 40
Day 60
2 µm
C
Day 20
Day 40
Day 60
D
ns
ns
*
Mean mtDNA copy number
600
400
200
0
D0
D10
D20
D40
D60
Fi
Day 20
Day 40
Day 60
ACTN2 GJA1 DAPI
10 µm
E
****
Sarcomere Length (µM)
2.5
2.0
1.5
1.0
0.5
D20
D40
D60
Fii
Day 20
Day 40
Day 60
ACTA2
10 µm

Figure S4. hPSC-LV-CMs mature fast *in vitro*. A Transmission electron micrographs of day 20 hPSC-LV-CMs. On the left, a zoomed-out view of a CM exhibiting organised sarcomeres can be seen. Scale bar: 10 μm. In the middle, is a zoomed-in view showing three adjacent cells. Within these, a possible desmosome (D), can be seen. A gap junction (GP) and some possible caveolae (C) can also be seen. Scale bar: 500 nm. On the right, clear sarcomere structures can be observed including the Z-disk, I-band, and M-line. Scale bar: 1 μm. **B** Transmission electron micrographs of day 20 (left), day 40 (middle) and day 60 (right) hPSC-LV-CMs. Images focused on sarcomeres. Scale bar: 2 μm. **C** Transmission electron micrographs of day 20 (left), day 40 (middle) and day 60 (right) hPSC-LV-CMs. Images focused on mitochondria. **D** Graph showing the mean mitochondrial DNA copy number of hPSC-LV-CMs at different times points (days, D) as determined using qPCR. * $p \leq 0.05$. **E** Graph showing the sarcomere length of hPSC-LV-CMs at days (D) 20, 40 and 60, as determine using transmission electron micrographs and Fiji. **F** Immunostaining micrographs of hPSC-LV-CMs at days 20, 40 and 60 stained for: ACTA2; the Gap-junction marker GJA1; DAPI (**Fi**) and the stress marker ACTA2 (**Fii**). Scale bar: 10 μm. (See also Figure 3)

Figure S5

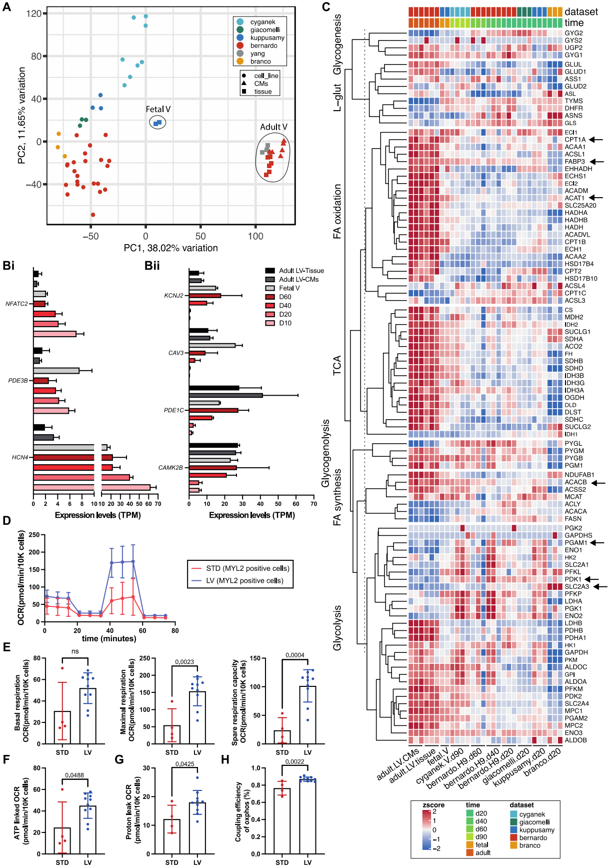
A
PC1, 38.02% variation
PC2, 11.65% variation
cyganek
giacomelli
kuppusamy
bernardo
yang
branco
cell_line
CMs
tissue
Fetal V
Adult V
Bi
Bii
NFATC2
PDE3B
HCN4
KCNJ2
CAV3
PDE1C
CAMK2B
Adult LV-Tissue
Adult LV-CMs
Fetal V
D60
D40
D20
D10
Expression levels (TPM)
C
dataset
time
Glycogenesis
L-glut
FA oxidation
TCA
Glycogenolysis
FA synthesis
Glycolysis
GYG2
GYS2
UGP2
GYG1
GLUL
GLUD1
ASS1
GLUD2
ASL
TYMS
DHFR
ASNS
GLS
ECI1
CPT1A
ACAA1
ACSL1
FABP3
EHHADH
ECHS1
ECI2
ACADM
ACAT1
SLC25A20
HADHA
HADHB
HADH
ACADVL
CPT1B
ECH1
ACAA2
HSD17B4
CPT2
HSD17B10
ACSL4
CPT1C
ACSL3
CS
MDH2
IDH2
SUCLG1
SDHA
ACO2
FH
SDHB
SDHD
IDH3B
IDH3G
IDH3A
OGDH
DLD
DLST
SDHC
SUCLG2
IDH1
PYGL
PYGM
PYGB
PGM1
NDUFAB1
ACACB
ACSS2
MCAT
ACLY
ACACA
FASN
PGK2
GAPDHS
PGAM1
ENO1
HK2
SLC2A1
PFKL
PDK1
SLC2A3
PFKP
LDHA
PGK1
ENO2
LDHB
PDHB
PDHA1
HK1
GAPDH
PKM
ALDOC
GPI
ALDOA
PFKM
PDK2
SLC2A4
MPC1
PGAM2
MPC2
ENO3
ALDOB
adult.LV.CMs
adult.LV.tissue
fetal.V
cyganek.V.d90
bernardo.H9.d60
bernardo.H9.d40
bernardo.H9.d20
giacomelli.d20
kuppusamy.d20
branco.d20
zscore
time
d20
d40
d60
d90
fetal
adult
dataset
cyganek
giacomelli
kuppusamy
bernardo
branco
D
OCR(pmol/min/10K cells)
time (minutes)
STD (MYL2 positive cells)
LV (MYL2 positive cells)
E
Basal respiration OCR(pmol/min/10K cells)
Maximal respiration OCR(pmol/min/10K cells)
Spare respiration capacity OCR(pmol/min/10K cells)
ns
0.0023
0.0004
STD
LV
F
ATP linked OCR (pmol/min/10K cells)
0.0488
G
Proton leak OCR (pmol/min/10K cells)
0.0425
H
Coupling efficiency of oxphos (%)
0.0022

8

Figure S5. hPSC-LV-CMs are more mature than time-matched cardiomyocytes generated using alternative protocols. A Principal component analysis (PC) of human adult tissues, human adult isolated CMs and hPSC-CMs generated by our lab (Bernardo) or by the indicated authors[2-5,7]. Ex-vivo samples were from the right or left ventricles and are indicated as Foetal V or Adult V. **B** Graphs showing the expression levels of the indicated genes in transcripts per million (TPM). **C** Heatmap showing the relative-expression levels of metabolism associated genes. The genes are organised in groups of metabolic associated activity (indicated on the left). Arrows point to genes highlighted in the main text. **D** Oxygen consumption rate (OCR) graph for day 20 ventricular hPSC-CMs isolated by flow cytometry (MYL2-GFP$^+$) from both LV and Std cultures.

E Respiration analysis of ventricular (MYL2-GFP$^+$) hPSC-CMs based on OCR. **F-H** Graphs showing the ATP production (**F**), proton leak (**G**) and coupling efficiency (**H**) of ventricular hPSC-CMs based on OCR. (See also Figure 4)

Figure S6

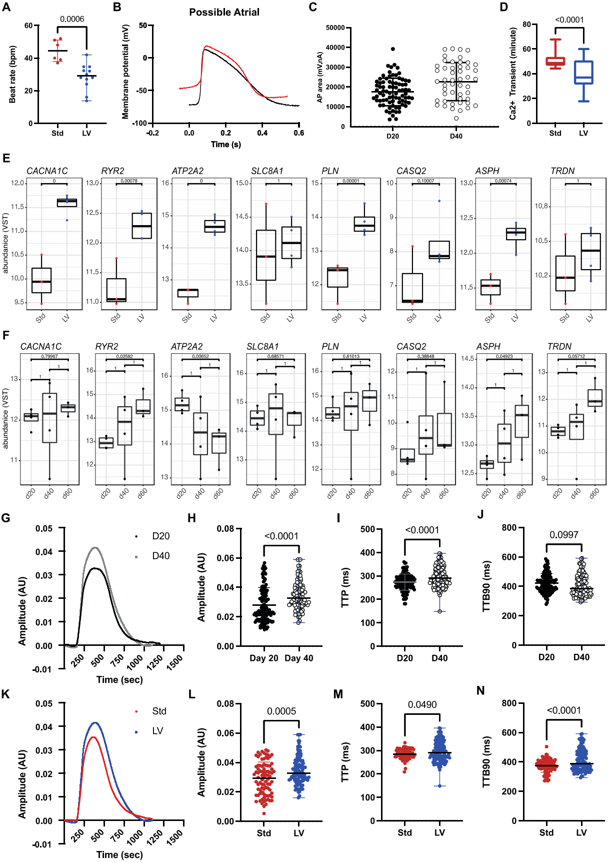
A
Beat rate (bpm)
0.0006
Std
LV
B
Possible Atrial
Membrane potential (mV)
Time (s)
C
AP area (mV.nA)
D20
D40
D
Ca2+ Transient (minute)
<0.0001
Std
LV
E
CACNA1C
RYR2
ATP2A2
SLC8A1
PLN
CASQ2
ASPH
TRDN
abundanice (VST)
F
CACNA1C
RYR2
ATP2A2
SLC8A1
PLN
CASQ2
ASPH
TRDN
abundanice (VST)
G
Amplitude (AU)
D20
D40
Time (sec)
H
Amplitude (AU)
<0.0001
Day 20
Day 40
I
TTP (ms)
<0.0001
D20
D40
J
TTB90 (ms)
0.0997
D20
D40
K
Amplitude (AU)
Std
LV
Time (sec)
L
Amplitude (AU)
0.0005
Std
LV
M
TTP (ms)
0.0490
Std
LV
N
TTB90 (ms)
<0.0001
Std
LV

10

Figure S6. hPSC-LV-CMs display hallmarks of electrophysiology and calcium handling maturity.

A Graph showing the beat rate of hPSC-CMs as calculated manually at day 20. **B** Graph showing the action potential (AP) morphology of a day (D) 20 and a day 40 hPSC-LV-CM cell with an atrial-like AP shape. Note these cells still exhibit a plateau but which is less marked than that of sibling cells thus making it unclear if they are atrial or more immature ventricular AP shapes. These are the types of cells counted as possible atrial within the morphological AP assessment of the data. **C** Graph showing the AP area of day 20 and day 40 hPSC-LV-CMs. **D** Graph showing the calcium transient rate of hPSC-CMs as calculated using Fluo4AM and CellOptic at day 20. **E-F** Graphs showing the expression data for calcium related genes between Standard (Std) and LV cultures (**E**) or within hPSC-LV-CMs of different ages, i.e. grown for a different number of days (d) (**F**). Indicated are the adjusted p-values from the DESeq2 analysis. **G-J** Calcium function characterization of day (D) 20/40 hPSC-LV-CMs. Data was analysed using optical mapping. **G** Example of calcium transients (CaT). **H** Graph showing the CaT amplitude. **I** Graph showing the CaT time to peak (TTP). **J** Graph showing the CaT time to baseline 90 (TTB90). **K-N** Calcium function characterization of day (D) 40 hPSC-CMs differentiated using the Standard (Std) or LV differentiation protocols. **K** Example of calcium transients (CaT). **L** Graph showing the CaT amplitude. **M** Graph showing the CaT time to peak (TTP). **N** Graph showing the CaT time to baseline 90 (TTB90). (See also Figures 5 and 6)

Figure S7

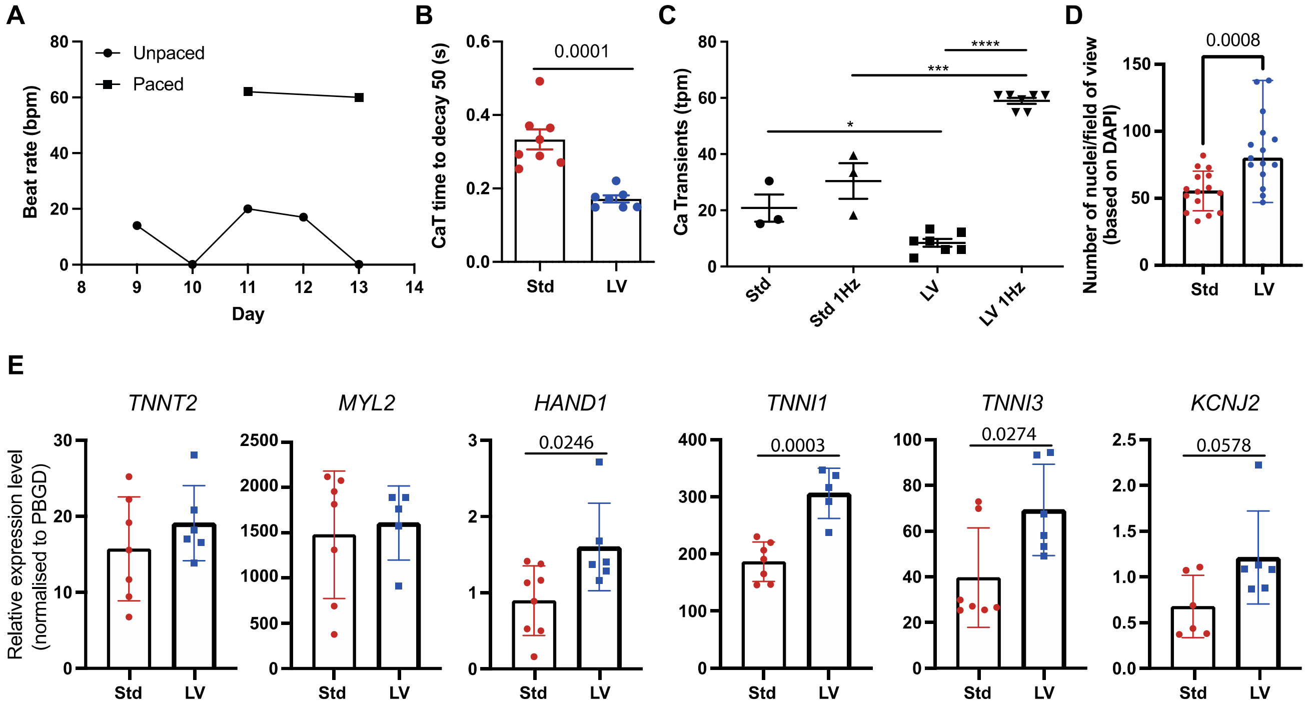

Figure S7. hPSC-LV-CMs generate more functional EHTs. Characterization of EHTs generated from day 40 hPSC-CMs differentiated using the Standard (Std) or LV differentiation protocols. **A** Graph showing an example of a LV-EHT with low to no spontaneous beating over time in culture and the respective beat rate it achieves when paced at 1 Hz. **B** Graph showing the calcium transient (CaT) analysis of EHTs as determined using confocal microscopy; CaT to decay 50 is indicated. **C** Graph showing the calcium transient (CaT), expressed as transient per minute (tpm), of EHTs beating spontaneously or paced at 1 Hz. **D** Graph showing the number of nuclei found per field of view within Std- and LV-EHTs. **E** Graphs showing the qRT-PCR expression data for various cardiac genes. Data was normalised to PBGD. (See also Figure 7)

Supplemental References

1. DeLaughter, D.M., Bick, A.G., Wakimoto, H., McKean, D., Gorham, J.M., Kathiriya, I.S., Hinson, J.T., Homsy, J., Gray, J., Pu, W., et al. (2016). Single-Cell Resolution of Temporal Gene Expression during Heart Development. Dev Cell *39*, 480-490. 10.1016/j.devcel.2016.10.001.
2. Cyganek, L., Tiburcy, M., Sekeres, K., Gerstenberg, K., Bohnenberger, H., Lenz, C., Henze, S., Stauske, M., Salinas, G., Zimmermann, W.H., et al. (2018). Deep phenotyping of human induced pluripotent stem cell-derived atrial and ventricular cardiomyocytes. JCI Insight *3*. 10.1172/jci.insight.99941.
3. Kuppusamy, K.T., Jones, D.C., Sperber, H., Madan, A., Fischer, K.A., Rodriguez, M.L., Pabon, L., Zhu, W.Z., Tulloch, N.L., Yang, X., et al. (2015). Let-7 family of microRNA is required for maturation and adult-like metabolism in stem cell-derived cardiomyocytes. Proc Natl Acad Sci U S A *112*, E2785-2794. 10.1073/pnas.1424042112.
4. Giacomelli, E., Meraviglia, V., Campostrini, G., Cochrane, A., Cao, X., van Helden, R.W.J., Krotenberg Garcia, A., Mircea, M., Kostidis, S., Davis, R.P., et al. (2020). Human-iPSC-Derived Cardiac Stromal Cells Enhance Maturation in 3D Cardiac Microtissues and Reveal Non-cardiomyocyte Contributions to Heart Disease. Cell Stem Cell *26*, 862-879 e811. 10.1016/j.stem.2020.05.004.
5. Branco, M.A., Cotovio, J.P., Rodrigues, C.A.V., Vaz, S.H., Fernandes, T.G., Moreira, L.M., Cabral, J.M.S., and Diogo, M.M. (2019). Transcriptomic analysis of 3D Cardiac Differentiation of Human Induced Pluripotent Stem Cells Reveals Faster Cardiomyocyte Maturation Compared to 2D Culture. Sci Rep *9*, 9229. 10.1038/s41598-019-45047-9.
6. Zhang, J.Z., Termglinchan, V., Shao, N.Y., Itzhaki, I., Liu, C., Ma, N., Tian, L., Wang, V.Y., Chang, A.C.Y., Guo, H., et al. (2019). A Human iPSC Double-Reporter System Enables Purification of Cardiac Lineage Subpopulations with Distinct Function and Drug Response Profiles. Cell Stem Cell *24*, 802-811 e805. 10.1016/j.stem.2019.02.015.
7. Yang, K.C., Yamada, K.A., Patel, A.Y., Topkara, V.K., George, I., Cheema, F.H., Ewald, G.A., Mann, D.L., and Nerbonne, J.M. (2014). Deep RNA sequencing reveals dynamic regulation of myocardial noncoding RNAs in failing human heart and remodeling with mechanical circulatory support. Circulation *129*, 1009-1021. 10.1161/CIRCULATIONAHA.113.003863.
